# The development of emergency medical services benefit score: a European Delphi study

**DOI:** 10.1186/s13049-021-00966-3

**Published:** 2021-10-16

**Authors:** Anssi Heino, Lasse Raatiniemi, Timo Iirola, Merja Meriläinen, Janne Liisanantti, Miretta Tommila, Collaboration Group, Andreas Krüger, Fabrice Dami, Didier Moens, Espen Fevang, Heini Harve-Rytsälä, Helena Jäntti, Jouni Nurmi, Kristin Tønsager, Leif Rognås, Marius Rehn, Patrick Schober, Per P. Bredmose, Peter Martin Hansen, Peter Temesvari, Søren Mikkelsen, Thomas W. Lindner, Troels Martin Hansen, Anna Nikula, Anne-Mari Kantanen, Antti E. Lindgren, Heli Salmi, Karri Kirjasuo, Marjut Varpula, Matti Reinikainen, Nanneli Paalasmaa, Outi Peltoniemi, Teemu Luoto, Vesa Lund, Ville Jalkanen

**Affiliations:** 1 Department of Perioperative Services, Intensive Care Medicine and Pain Management, Turku University Hospital, Turku, Finland; 2 University of Turku, Turku, Finland; 3 Centre for Prehospital Emergency Care, Oulu University Hospital, Oulu, Finland; 4 Emergency Medical Services, Turku University Hospital, Turku, Finland; 5 Medical Research Centre, Oulu University Hospital, Oulu, Finland; 6 Department of Anaesthesiology, Medical Research Centre and Research Group of Anaesthesia and Intensive Care, University of Oulu, Oulu University Hospital, Oulu, Finland

**Keywords:** Prehospital, Emergency medical services, Delphi method, Quality control

## Abstract

**Background:**

The helicopter emergency services (HEMS) Benefit Score (HBS) is a nine-level scoring system developed to evaluate the benefits of HEMS missions. The HBS has been in clinical use for two decades in its original form. Advances in prehospital care, however, have produced demand for a revision of the HBS. Therefore, we developed the emergency medical services (EMS) Benefit Score (EBS) based on the former HBS. As reflected by its name, the aim of the EBS is to measure the benefits produced by the whole EMS systems to patients.

**Methods:**

This is a four-round, web-based, international Delphi consensus study with a consensus definition made by experts from seven countries. Participants reviewed items of the revised HBS on a 5-point Likert scale. A content validity index (CVI) was calculated, and agreement was defined as a 70% CVI. Study included experts from seven European countries. Of these, 18 were prehospital expert panellists and 11 were in-hospital commentary board members.

**Results:**

The first Delphi round resulted in 1248 intervention examples divided into ten diagnostic categories. After removing overlapping examples, 413 interventions were included in the second Delphi round, which resulted in 38 examples divided into HBS categories 3–8. In the third Delphi round, these resulted in 37 prehospital interventions, examples of which were given revised version of the score. In the fourth and final Delphi round, the expert panel was given an opportunity to accept or comment on the revised scoring system.

**Conclusions:**

The former HBS was revised by a Delphi methodology and EBS developed to represent its structural purpose better. The EBS includes 37 exemplar prehospital interventions to guide its clinical use.

*Trial registration* The study permission was requested and granted by Turku University Hospital (decision number TP2/010/18).

**Supplementary Information:**

The online version contains supplementary material available at 10.1186/s13049-021-00966-3.

## Background

Evaluating the potential benefits of emergency medical services (EMS) missions is crucial to allocate EMS resources purposefully and to focus the dispatching of advanced-level prehospital units to missions where patients are likely to benefit from their advanced skills. Due to the multifaceted nature of prehospital missions, the benefits of prehospital care are difficult to evaluate [1, 2], and the benefits of advanced prehospital care are continuously subject to debate [3]. Existing scoring systems estimate the severity of injuries or illnesses for patients, such as the National Advisory Committee for Aeronautics (NACA) severity score [4], which focuses on the severity of an incident and patient characteristics and does not consider the impact of prehospital care, limiting its use in benchmarking and benefit assessments.

The helicopter emergency medical services (HEMS) Benefit Score (HBS) is a nine-level scoring system developed to evaluate the benefits of HEMS missions in the 1990s in Finland [5]. Each category is defined by a written description along with exemplar interventions which can be used to guide the scorer's choice of category. The highest HBS score is reserved for the most advanced prehospital interventions, but the idea is to evaluate the benefit produced by the whole EMS system, not only HEMS units. The scoring system has been used in the Finnish HEMS units since 1997, originally to follow the benefit of the HEMS launched at that time, but nowadays also to compare individual national HEMS units and to collect data for administration purposes.

Despite the everyday use of the HBS in Finnish HEMS for over two decades, its validity has not been studied at all, and reliability has been studied only recently [5, 6]. According to study results, the HBS’s inter-rater reliability was noticed to vary from poor to substantial or almost perfect, and mean difference between raters and reference values were substantial [5, 6]. As the scoring is guided by exemplar interventions, it can be argued, that the reliability could be improved by more detailed and comprehensive examples. Additionally, it has been suggested, that the exemplar interventions should be updated to meet the current treatment guidelines [5].

## Methods

### Aim

The aim of this study is to develop a score to measure the benefits of prehospital interventions to a single patient. This score development is based on the HBS, but the old exemplar interventions are replaced by more relevant examples. The meaning of these updated instructions is to cover the most common prehospital mission types and make evaluating the effectiveness of prehospital treatments easier and more accurate. Because this evaluation tool is appropriate for the whole EMS system, the score is renamed the EMS Benefit Score (EBS).

### Design and setting

This is a four-round, web-based, international Delphi study using expert panel consensus. The technique involves a panel of experts who are asked to complete a series of questionnaires focusing on their opinions, predictions and judgements on a topic of interest. The Delphi technique is widely used in health research to obtain consensus in serial surveys, which are referred to as “rounds”. Key elements of the technique are (1) expert participants, (2) anonymity and individuality, and (3) a summary of results of the former round at the start of each round [7, 8]. The data collection, Delphi rounds and data analysis of the current study were performed from 3.12.2018 to 19.11.2020. A pilot study was performed prior to the actual study to evaluate the study setting. The pilot study participants consisted of Finnish and Danish prehospital physicians who did not participate in the planning of the study or in the actual study.

The work of the expert panel and the commentary board were executed in four Delphi rounds as follows:

1. Each expert panellist was asked to list both common and rare examples of prehospital treatments and interventions and to locate them based on their current knowledge and personal experience into HBS categories 3–8 as comprehensively as possible in subsections based on ten complaint-based diagnoses: “acute neurology excluding stroke”, “breathing difficulties”, “cardiac arrest”, “chest pain”, “infection”, “obstetrics including child birth”, “other”, “psychiatry including intoxication”, “stroke” and “trauma”. These diagnosis groups are recommended in prehospital reporting [9]. The answers were collected anonymously into an electronic data sheet by a data-collection officer who did not participate in the example selection but gathered suggestions in a common table. HBS categories 0–2 were excluded from the study because they are used for scoring when a prehospital intervention is deemed unnecessary or the patient was not met. A commentary board commented on the data gathered from the first Delphi round on the diagnosis groups related to their individual specialties. These comments were shown to the expert panel in the second Delphi round to help them rate the examples on a 5-point Likert scale. Identical suggestions from the first round were combined and overlapping examples removed for the second Delphi round.
2. The examples from the first Delphi round with the commentary board’s opinions were set in a table and sent back to the panellists, who were asked to rate each item on a 5-point Likert scale from 1 (*strongly disagree*) to 5 (*strongly agree*). A content validity index (CVI) was calculated for each example, and at least 70% of the experts were required to assign a suggested example a high-agreement score (4 or 5) for it to be included in the third Delphi round. Overlapping examples were then removed.
3. In the third Delphi round, the remaining examples were listed in their suggested HBS categories. The expert panellists were asked to assign each of these remaining examples one of the following labels: “Accept”, “Delete” or “Relocate to EBS category number __”. An acceptance rate of 70% or more was required to assign an example to a category. The examples with acceptance rates below 70% were deleted or relocated to category with the most “Relocate” suggestions—whichever had the higher percentage.
4. In the final Delphi round, the EBS was revealed to the prehospital expert panellists, who were offered an opportunity to comment on it or accept it in that form.

In addition to these Delphi rounds, each phase included an opportunity for free comments on the exemplar interventions and category descriptions.

### Participants

Two expert groups were formed for the study: a prehospital expert panel and a separate commentary board. Experts were recruited with open letters: the prehospital expert panel via the European Prehospital Research Alliance (EUPHOREA) and the commentary board via National Finnish specialty societies. The participants were selected based on individual clinical and scientific experiences. The prehospital expert panel ultimately included 18 prehospital physicians from Scandinavia and Northern Europe and the commentary board 11 Finnish in-hospital physicians from seven specialties. The total number of study experts was 29. Table 1 presents characteristics of the 18 prehospital expert panellists. Physicians from intensive care, traumatology, cardiology, neurology, neurosurgery, paediatrics and obstetrics were recruited for the commentary board. Members of the commentary board were recruited to give an in-hospital viewpoint, and therefore they did not have prior or current prehospital experience.

**Table 1:** Characteristics of the 18 prehospital expert panellists

Speciality	n	%	Clinical experience in prehospital critical care (years)	n	%	Number of peer reviewed publications	n	%
Anaesthesiology	6	33	5–10	1	6	Less than five	4	22
Anaesthesiology and intensive care	8	44	10–15	9	50	5–10 publications	2	11
Emergency medicine	3	17	15–20	4	22	10–20 publications	5	28
Anaesthesiology, intensive care and emergency medicine	1	6	Over 20	4	22	More than 20 publications	7	39

### Statistical methods

This study used the Delphi method and expert consensus. Data handling and collection were performed using Webropol 3.0 by the Webropol Group. A 5-point Likert scale was used on the second Delphi round, and a CVI was calculated for the collected data by Webropol 3.0. Agreement was defined as 70% of the experts rating a suggested example with a high-agreement score (4 or 5) [10].

### Ethics

By Finnish law, no ethical approval was needed for this study because no patients or personal data were involved. The study permission was requested and granted by Turku University Hospital (decision number TP2/010/18). The study subjects participated voluntarily. The Standards for Reporting Qualitative Research (SRQR) guidelines by the EQUATOR network were followed in reporting the study.

### Patient and public involvement

No patients were involved.

## Results

**Fig. 1:**
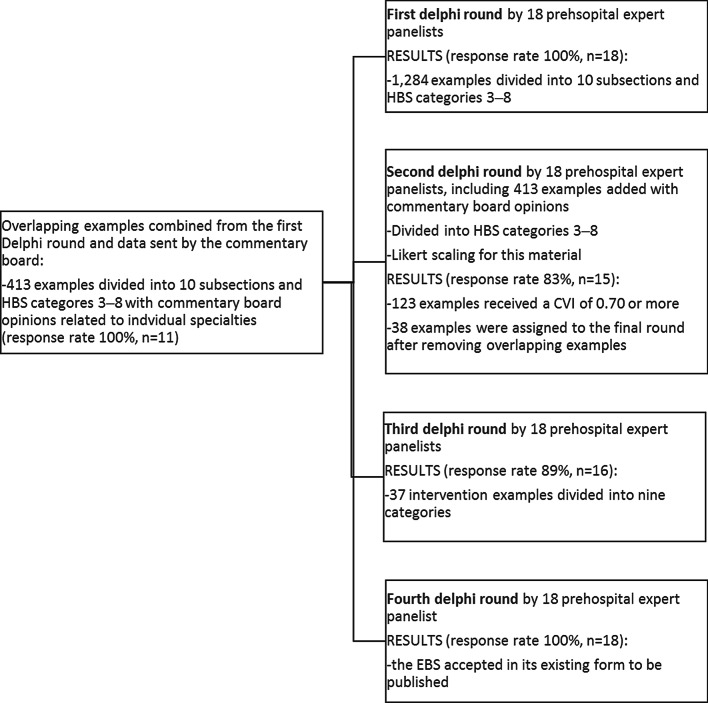
The course of the Delphi rounds in the study

The first Delphi round resulted in 1284 examples from 18 expert panellists divided into HBS categories 3–8 in ten complaint-based subsections. Seven of the responders gave free comments (each Delphi round included sections for free written comments). Figure 1 describes the course of the Delphi rounds, and Additional files 1 and 2 present the materials of the second and third Delphi rounds (Additional files 1 and 2).

Table 2 presents the final form of the scoring system, and additional materials present the expert panellists’ free comments. The definitions of the score categories were kept in their original forms, and no free comment was related to the content of these written definitions. In the fourth Delphi round, one participant suggested moving “Administration of tranexamic acid” from EBS 4 to EBS 6 based on current scientific evidence, and this manoeuvre was performed.

**Table 2:** The EBS

EBS	Description	Exemplar interventions*
0	The patient was not seen	
1	Prehospital care was not deemed necessary	
2	Prehospital care apparently had no significance from the patient’s standpoint (e.g., cannulation, no medication or fluid therapy) or, despite prehospital care, the patient died before reaching the hospital	
3	Prehospital care apparently had no significance from the standpoint of the prognosis, but the patient’s symptoms or pain was alleviated (e.g., injured patient’s analgesia)	Administration of analgesics
		Administration of antihistamines to treat an allergic reaction
		Antiemetic medication
4	Prehospital care was administered; its significance from the patient’s standpoint is unknown, difficult to assess or only assessable retrospectively (e.g., treatment of ischaemic chest pain, brief convulsions and mild breathing difficulty)	Trauma patient immobilisation (cervical collar, back board, etc.)
		Administration of inhaled bronchodilators for COPD or pneumonia
		Administration of oxygen in moderate breathing difficulty
5	Without prehospital care (administered by the first response unit or the physician-staffed unit), the patient would have died before reaching the hospital, but he/she was assessed as having a poor prognosis (e.g., serious brain damage, coma caused by spontaneous cerebral haemorrhage, primary survival from cardiac arrest after lengthy response times and terminal phase of a malignant disease)	Patient treated but due to severe symptoms and/or underlying diseases has a poor prognosis (e.g., severe trauma or traumatic cardiac arrest, severe hypoxic insult, prolonged resuscitation and cardiac arrest due to severe traumatic brain injury or subarachnoid haemorrhage)
6	The patient was given prehospital care that can be assessed to reduce mortality or otherwise improve the prognosis	Administration of physician-staffed EMS-level medication (medication not allowed in other units) followed by relief of signs and symptoms
		Administration of tranexamic acid
		Medication for circulatory support (i.v. ephedrine, i.v. noradrenaline or norepinephrine, etc.)
		Treatment of prolonged seizures by first- or second-line i.v. medication (bentsodiazepines, phosphenytoin, etc.)
		Treatment of hypoglycaemia-induced coma or seizures by i.v. glucose or s.c./i.m. glucagon
		Treatment of hypoglycaemia by i.v. glucose or s.c./i.m. glucagon when patient is disoriented but not in coma
		Reduction and stabilisation of fractures or luxations
		Triage and patient selection to dedicated centre and rapid transportation (major trauma, TBI, need of thrombectomy, need for re-implantation in traumatic amputation, etc.)
		Treatment of opioid or benzodiazepine poisoning by antidotes
		Maternal positioning in case of prolapsed umbilical cord
		Thrombolysis for STEMI in cases with long transportation times
		Rapid transportation to PCI
7	Without prehospital care (administered by the first response unit or the physician-staffed unit), the patient would have died before reaching the hospital, and he/she cannot be assessed as having a poor prognosis	Mass casualty incident leadership and triage
		Treatment and stabilising of a multi-trauma patient in shock by i.v. fluid administration and/or vasoactive medication
		Isolated severe trauma managed with simple manoeuvres (e.g., direct compression and tourniquet)
		Needle thoracocentesis followed by a relief of signs and symptoms
		Cardioversion or cardiac pacing
		Medication (adrenalin/epinephrine) in anaphylactic shock and relief of signs and symptoms
		Successful resuscitation with reasonable prognosis
		Transfer to ECMO, bypass or angiography during CPR
		Manual opening of an obstructed airway and bag-mask ventilation
		Use of a supraglottic device and bag-mask ventilation
8	Category 7 in situations where other emergency medical staff on site would not have been capable of administering the aforementioned life-saving treatment (use of physician-staffed EMS unit or advanced trained paramedic unit in systems where licenced to perform)	Thoracotomy or tamponade release with other manoeuvres
		Thoracostomy or pleural drainage followed by relief of signs and symptoms
		ECMO initiation in prehospital phase (ECPR)
		Management of complicated childbirth (shoulder dystocia, malposition, etc.)
		Prehospital Caesarean section (resuscitative hysterectomy)
		Resuscitation of a newborn by bag-mask ventilation or by more advanced procedures
		Rapid sequence intubation or surgical airway management and mechanical ventilation
		Blood product transfusions

*COPD* chronic obstructive pulmonary disease, *ECMO* extracorporeal membrane oxygenation, *ECPR* extracorporeal cardiopulmonary resuscitation, *CPR* cardiopulmonary resuscitation, *STEMI* ST-elevation myocardial infarction

*Interventions are listed as examples for each category; determination of the correct EBS is made by the prehospital clinician in charge of patient care

## Discussion

In this study, we updated the HEMS Benefit Score by using the Delphi method to meet the current needs of prehospital emergency care. The structure of nine-level numerical scoring categories, inherited from the original HBS, remained intact, but the exemplar interventions in each category were totally renovated. With this renewal, the scoring system was expanded from HEMS usage to cover all prehospital emergency care, including non-HEMS units, and to better face present-day needs. The renamed score, EBS, better represents the fundamental features of this scoring system and encourages non-HEMS units to utilise it in their practice.

The EBS focuses on interventions that are performed prehospitally and considers the impact of these manoeuvres for treated patients. By this, the EBS aims to evaluate the true benefit of EMS for single patients. In contrast, other scores and classifications used in prehospital settings, such as the American Society of Anesthesiologists Physical Status Classification System (ASA-PS) or NACA [5, 6, 9], describe patient background characteristics and acute clinical status. However, these scores do not evaluate the influence of prehospital care and were not originally built or implemented for prehospital use, so their reliability in prehospital settings is questionable [6].

The revised scoring examples are expected to improve correct benefit category selection. After each EMS mission, EMS personnel responsible for mission documenting, choose a suitable benefit category depending on the individual mission circumstances. Even though the revised examples introduce the consensus opinion of the experts and give guidelines to the benefit category selection, the scoring is ultimately based on the subjective judgement of the person doing documentation. This is because the revised examples are obviously not comprehensive, even if they are versatile. Additionally, it is justifiable to deviate from the score suggested by the exemplar interventions, if the patient has, for example, benefited from several interventions or fast air transport or, on the other hand, the interventions performed have been unnecessary or ineffectual. Despite the subjective nature of the EBS, it can serve as a valuable tool for gathering information from one aspect of prehospital missions, as the effectiveness of prehospital emergency care is a highly complex ensemble and a totally inclusive scoring system for this purpose does not exist.

During the Delphi process, the benefit category examples were renovated, but the numerical scoring categories remained intact, as it was judged unreasonable to evaluate the number of the categories during the same process. These numerical categories were originally developed based on practical experience, so there is no science behind them, and they or the number of them might be inappropriate. This issue must be taken into account in the future studies, and one must estimate the need of possible revision of the categories.

To evaluate the effectiveness of prehospital care, various quality indicators and measurement protocols have been launched [1, 11–13], but few studies have focused on their implementation or outcomes. A single scoring system does not solve the absence of process control in EMS systems, but combined with other manoeuvres, the EBS can support intrinsic quality improvement. For example, data on EMS unit-dispatch codes and criteria can be compared on EBSs and the benefit produced by EMS to prehospitally treated patients, based on interpretation of a treating clinician. Beyond accurately dispatching the proper level and number of EMS units, however, EMS system coverage and the geographic locating of units remain challenges [14, 15]. The type and number of missions historically presented in the areas under observation are important aspects in locating EMS units and bases. With the EBS, additional information on regional missions can be gathered. However, far-reaching conclusions based on the EBS are not justified until its reliability and validity have been studied in various settings.

### Strengths and limitations

The international expert panel improved the EBS’s generalisability. Despite variations in EMS systems between countries, the EBS evaluates the potential advantages for prehospital patients regardless of the level of the treating EMS unit, the only exception being the highest EBS category, which is reserved for treatments usually offered by only advanced-level units.

The Delphi technique in this study enabled a panel of 18 experienced panellists to express their opinions freely and impersonally guided by the opinions of 11 in-hospital experts from seven specialties. This method limits dominance by eminent, eloquent or highly opinionated individuals in their respective fields of expertise [7, 8], and the panel moderator is less likely to bias the work of the panel. The Delphi method gives panellists substantial time to express their ideas, reflect on their answers and make changes, P and it avoids geographical constraints. On the other hand, the Delphi method itself is vulnerable to a loose definition of an expert, and biases might influence participant selection. The method is also dependent on questionnaire design [7, 8].

A major limitation of this study is, that there is limited data on the impact of several prehospital interventions such as prehospital airway management [16, 17]. An intervention may or may not be life-saving, depending on context. However, in the absence of a thorough research-based data on the impact of different interventions, a consensus opinion of experts is meaningful. In addition, currently no evidence exists of paramedics` ability to predict mortality.

### Implications

The EBS is based on the subjective opinion of an attending prehospital clinician. To make the scoring system less dependent on individual variation, the renewed exemplar interventions in each EBS category support the selection of the appropriate category. The revised EBS can be used to benchmark different types of units, enabling quality control, which also allows the development of EMS efficiency. The given EBS scores can be compared to in-hospital interventions and patient outcome, to evaluate the adequacy of prehospital care. For example, a person unconscious due to alleged alcohol intoxication has been given EBS 2 on paramedic evaluation but needs rapid sequence intubation upon arrival in the emergency department. In this case EBS could be used to detect and study why this has happened, and this way for system quality control. Moreover, if the patients with low EBS receive intensive care or emergency procedures in hospital, this should raise the question of the quality of prehospital evaluation of the patients’ condition. Finally, this scoring system can be used to categorize prehospital interventions in clinical studies on EMS performance and to get more data where and in which type of missions, the patients are likely to benefit most. In the future EBS could optimally be linked to the care patient receive in hospital and their later level of performance. However, further reliability and validity studies are needed, before a wide-scale implementation.

## Conclusion

Using the Delphi method, the new scoring system, the EBS, was formed by a panel of experienced experts from across Northern Europe. We recommend implementing the EBS to every EMS systems as a part of a routine reporting.

## Supplementary Information

**Additional file 1.** The material of second Delphi round: specialty comments, and Likert-Scale distribution of intervention examples given by prehospital experts.

**Additional file 2.** The material of third Delphi round.

## Data Availability

The datasets analysed in this study are available from the corresponding author upon reasonable request.

## Abbreviations

EBS: EMS Benefit Score
EMS: Emergency medical service
EUPHOREA: European Prehospital Research Alliance
HBS: HEMS Benefit Score
HEMS: Helicopter emergency medical service
P-EMS: Physician-staffed emergency medical service
SRQR: Standards for Reporting Qualitative Research

## References

[1] Murphy A, Wakai A, Walsh C (2016). Development of key performance indicators for prehospital emergency care. Emerg Med J.

[2] Saviluoto A, Björkman J, Olkinuora A (2020). The first seven years of nationally organized helicopter emergency medical services in Finland—the data from quality registry. Scand J Trauma Resusc Emerg Med.

[3] McLean SA, Maio RF, Spaite DW (2009). Emergency medical services outcomes research: evaluating the effectiveness of prehospital care. Prehosp Emerg Care.

[4] Raatiniemi L, Mikkelsen K, Fredriksen K (2013). Do pre-hospital anaesthesiologists reliably predict mortality using the NACA severity score? A retrospective cohort study. Acta Anaesthesiol Scand.

[5] Raatiniemi L, Liisanantti J, Tommila M (2017). Evaluating helicopter emergency medical missions: a reliability study of the HEMS benefit and NACA scores. Acta Anesthesiol Scand.

[6] Heino A, Laukkanen-Nevala P, Raatiniemi L (2020). Reliability of prehospital patient classification in helicopter emergency medical service missions. BMC Emerg Med.

[7] Polit D, Beck C. Nursing research—principles and methods. Lippincott, Williams & Wilkins/Wolters Kluwer; 2004.

[8] Diamond I, Grant R, Feldman B (2014). Defining consensus: a systematic review recommends methodologic criteria for reporting of Delphi studies. J Clin Epidemiol.

[9] Kruger AJ, Lockey D, Kurola J (2011). A consensus-based template for documenting and reporting in physician-staffed pre-hospital services. Scand J Trauma Resusc Emerg Med.

[10] Polit D, Beck C, Owen S (2007). Focus on research methods. Is the CVI an acceptable indicator of content validity? Appraisal and recommendations. Res Nurs Health.

[11] Cairns CB, Garrison HG, Hedges JR (1998). Development of new methods to assess the outcomes of emergency care. Acad Emerg Med.

[12] Haugland H, Rehn M, Klepstad P (2017). Developing quality indicators for physician-staffed emergency medical services: a consensus process. Scand J Trauma Resusc Emerg Med.

[13] Rehn M, Krüger AJ (2014). Quality improvement in pre-hospital critical care: increased value through research and publication. Scand J Trauma Resusc Emerg Med.

[14] Pappinen J, Laukkanen-Nevala P, Mäntyselkä P (2018). Development and implementation of a geographical area categorisation method with targeted performance indicators for nationwide EMS in Finland. Scand J Trauma Resusc Emerg Med.

[15] Røislien J, Van den Berg PL, Lindner T (2018). Comparing population and incident data for optimal air ambulance base locations in Norway. Scand J Trauma Resusc Emerg Med.

[16] Fullerton JN, Roberts KJ, Wyse M (2011). Should non-anaesthetists perform pre-hospital rapid sequence induction? An observational study. Emerg Med J.

[17] Van der Velden MWA, Ringburg AN, Bergs EA (2008). Prehospital interventions: time wasted or time saved? An observational cohort study management in initial trauma care. Emerg Med J.

