# Supplementary material for: The development of emergency medical services benefit score: a European Delphi study
Source: Scand J Trauma Resusc Emerg Med. 2021 Oct 16;29:151. doi: 10.1186/s13049-021-00966-3 (PMC8520267; doi:10.1186/s13049-021-00966-3)
Supplement: Supplementary file 1 — Additional file 1. The material of second Delphi round: specialty comments, and Likert-Scale distribution of intervention examples given by prehospital experts. [file 13049_2021_966_MOESM1_ESM.pdf]

Additional file 1. The second Delphi round: specialty comments, and Likert-Scale distribution of intervention examples given by prehospital experts

Total number of responders: 15

HBS 8

Severe facial injuries and risk of obstructed airway, or already obstructed airway
Traumatologist: great benefit
Intensivist 1: Very Rarely
Intensivist 2: Need for managing airway (in severe cases requires physician) without ETT. Obstructing aspiration of blood by positioning and suction.
Pediatrician 1: in case of need to secure airway
Pediatrician 2: Continuous monitoring of oxygenation, breathing, ventilatio,readiness for difficult airway management,treatment of pain,cervical injury?
Number of responders: 14

	**5**	**4**	**3**	**2**	**1**		**Total**	**Average**	**Median**
Strongly agree	11	3	0	0	0	Strongly disagree	14	4,79	5
	78,57%	21,43%	0%	0%	0%				
Total	11	3	0	0	0		14	4,79	5

Thoracotomy or tamponade release with other maneuvers (etc. punction)
Traumatologist: great benefit
Number of responders: 14

	**5**	**4**	**3**	**2**	**1**		**Total**	**Average**	**Median**
Strongly agree	12	1	1	0	0	Strongly disagree	14	4,79	5
	85,72%	7,14%	7,14%	0%	0%				
Total	12	1	1	0	0		14	4,79	5

Thoracostomy or pleural drainage
Traumatologist: great benefit
Intensivist 1: Very rarely
Intensivist 2: If needle thoracosentesis unsuccesful and hemodynamically unstable patients and/or severe respiratory failure incl. resuscitation
Pediatrcian 1: Vital importance (most important would be to suspect and be able to diagnose correctly - in small children, any attempt to insert a chest drain in case of no pneumothorax would be very detrimental in contrast to adults)
Pediatrician 2: In cases of tension pneumothx
Pediatrcian 3: only if suspicion of tensionpneumothorax or vital indication
Number of responders: 14

	5	4	3	2	1		Total	Average	Median
Strongly agree	8	6	0	0	0	Strongly disagree	14	4,57	5
	57,14%	42,86%	0%	0%	0%				
Total	8	6	0	0	0		14	4,57	5

Rapid sequence intubation or surgical airway and mechanical ventilation
Traumatologist: minor benefit
Intensivist 1: Sometimes yes
Intensivist 2: Instable patients eg. hemodynamical, respiratory or neurological instability. Difficult airway and/or need for surgical airway.
Pediatrician 1: Very important, but most important would be to be able to mask ventilate and use LMA - intubation may not be needed in many children and infants as mask ventilation and/or LMA is almost always feasible and associated to much less complications
Pediatrician 2:in cases of life-threatening hypoxemia/hypoventilation.videolaryngoscopy is an aoption in cases of difficult airway management.subraclottic devices if intubation fails (LMA contraindicated in laryngeal airway obstruction; epiglottitis, croup, laryngospasm).Cervical spine in-line immobilization during intubation in trauma patients
Pediatrcian 3: vital indication
Number of responders: 14

	5	4	3	2	1		Total	Average	Median
Strongly agree	9	3	1	1	0	Strongly disagree	14	4,43	5
	64,29%	21,43%	7,14%	7,14%	0%				
Total	9	3	1	1	0		14	4,43	5

Blood product transfusions
Traumatologist: great benefit
Number of responders: 14

	5	4	3	2	1		Total	Average	Median
Strongly agree	6	6	2	0	0	Strongly disagree	14	4,29	4
	42,86%	42,86%	14,28%	0%	0%				
Total	6	6	2	0	0		14	4,29	4

Physician-staffed EMS level medication
Traumatologist: great benefit
Gynecologist: agree. in some situations trained staff can use according to instructions by a physician, when physician is not available shortly.
Number of responders: 14

	5	4	3	2	1		Total	Average	Median
Strongly agree	5	6	2	1	0	Strongly disagree	14	4,07	4
	35,71%	42,86%	14,29%	7,14%	0%				
Total	5	6	2	1	0		14	4,07	4

Helicopter unit needed for rapid transportation and benefit of reduced time
Traumatologist: minimal benefit
Number of responders: 14

	5	4	3		1		Total	Average	Median
Strongly agree	6	7	0	1	0	Strongly disagree	14	1,71	2
	42,86%	50%	0%	7,14%	0%				
Total	6	7	0	1	0		14	1,71	2

Pre-hospital REBOA
Traumatologist: minor benefit
Number of responders: 14

	5	4	3	2	1		Total	Average	Median
Strongly agree	5	3	3	0	3	Strongly disagree	14	3,5	4
	35,71%	21,43%	21,43%	0%	21,43%				
Total	5	3	3	0	3		14	3,5	4

Major fracture reductions
Traumatologist: great benefit
Number of responders: 14

	5	4	3	2	1		Total	Average	Median
Strongly agree	1	6	1	5	1	Strongly disagree	14	3,07	3,5
	7,14%	42,86%	7,14%	35,72%	7,14%				
Total	1	6	1	5	1		14	3,07	3,5

Ultrasound diagnostics
Traumatologist: minimal benefit
Number of responders: 14

	5	4	3	2	1		Total	Average	Median
Strongly agree	3	3	2	2	4	Strongly disagree	14	2,93	3
	21,43 %	21,43 %	14,28 %	14,29 %	28,57 %				
Total	3	3	2	2	4		14	2,93	3

Severe drowning
Traumatologist: minor benefit
Number of responders: 14

	5	4	3	2	1		Total	Average	Median
Strongly agree	3	5	2	4	0	Strongly disagree	14	3,5	4
	21,43%	35,71%	14,29%	28,57%	0%				
Total	3	5	2	4	0		14	3,5	4

Prehospital amputation
Traumatologist: minor benefit
Number of responders: 14

	5	4	3	2	1		Total	Average	Median
Strongly agree	6	0	3	3	2	Strongly disagree	14	3,36	3
	42,86%	0%	21,43%	21,43%	14,28%				
Total	6	0	3	3	2		14	3,36	3

Unconscious patient
Traumatologist: great benefit
Number of responders: 14

	5	4	3	2	1		Total	Average	Median
Strongly agree	6	3	3	2	0	Strongly disagree	14	3,93	4
	42,86%	21,43%	21,43%	14,28%	0%				
Total	6	3	3	2	0		14	3,93	4

Control maneuvers for major haemorrhage

Traumatologist: minor benefit
Number of responders: 14

	5	4	3	2	1		Total	Average	Median
Strongly agree	7	4	0	3	0	Strongly disagree	14	4,07	4,5
	50%	28,57%	0%	21,43%	0%				
Total	7	4	0	3	0		14	4,07	4,5

Tranexamic acid administration
Traumatologist: maybe benefit
Neurologist 1: In spontaneous or traumatic intracranial bleeding the role of tranexamic acid (TXA) is questionable. Scientific evidence does not show any clear improvement in outcome, however hemorrhage progression seem to be decreased in patients receiving tranexamic acid. The first findings of the CRASH-3 study (TXA in traumatic brain injury) were just published (Lancet). They show that TXA could reduce mild-moderate TBI mortality and that immediate admistration is most beneficial.
Neurologist 2: Might benefit, if strong anamnesis
Pediatrician 1: Normally not needed in children for these purposes (may be used in children for nasal bleeding etc)
Pediatrician 2: for severe bleeding
Number of responders: 14

	5	4	3	2	1		Total	Average	Median
Strongly agree	2	2	4	5	1	Strongly disagree	14	2,93	3
	14,29%	14,29%	28,57%	35,71%	7,14%				
Total	2	2	4	5	1		14	2,93	3

Severe burn patients
Traumatologist: great benefit
Number of responders: 14

	5	4	3	2	1		Total	Average	Median
Strongly agree	5	7	1	1	0	Strongly disagree	14	4,14	4
	35,72%	50%	7,14%	7,14%	0%				
Total	5	7	1	1	0		14	4,14	4

Multi-trauma with shock
Traumatologist: maybe benefit
Number of responders: 13

	5	4	3	2	1		Total	Average	Median
Strongly agree	10	1	2	0	0	Strongly disagree	13	4,62	5
	76,92%	7,69%	15,39%	0%	0%				
Total	10	1	2	0	0		13	4,62	5

Any physician-staffed EMS level maneuver
Traumatologist: maybe benefit
Number of responders: 14

	5	4	3	2	1		Total	Average	Median
Strongly agree	3	4	5	2	0	Strongly disagree	14	3,57	3,5
	21,43%	28,57%	35,71%	14,29%	0%				
Total	3	4	5	2	0		14	3,57	3,5

Severe intoxication
Intensivist 1: Very rarely
Intensivist 2: Severe hemodynamic and neurological collapse. Severe arrhytmias. Need for opening the airway and hemodynamical instability. Some antidotes. Severe acidosis eg. methanol intoxications. TdP after intoxication.
Number of responders: 14

	5	4	3	2	1		Total	Average	Median
Strongly agree	1	5	4	3	1	Strongly disagree	14	3,14	3
	7,14%	35,72%	28,57%	21,43%	7,14%				
Total	1	5	4	3	1		14	3,14	3

Transportation to ECMO or prehospital-ECMO
Intensivist 1: Very rarely
Intensivist 2: Any ECMO patients, VV- or AV. I find prehospital ECMO as a rarity and should be considered only in very populated areas like London or Manhattan.Cardiologist: Score 8. In case of refractory of recurrent VF, the transfer to hospital with ECMO capasity and ECPR protocol, and start of ECMO treatment within 60 (- 90) from cardiac arrest might be life saving. Physician is needed for decision of ECPR.
Number of responders: 14

	5	4	3	2	1		Total	Average	Median
Strongly agree	9	2	1	2	0	Strongly disagree	14	4,29	5
	64,28%	14,29%	7,14%	14,29%	0%				
Total	9	2	1	2	0		14	4,29	5

Physician-staffed EMS level drug administration and/or maneuvers (etc. pacing)
Intensivist 1: Very rarely
Intensivist 2: Overpacing, severe bradycardia
Number of responders: 14

	5	4	3	2	1		Total	Average	Median
Strongly agree	4	8	0	2	0	Strongly disagree	14	4	4
	28,57%	57,14%	0%	14,29%	0%				
Total	4	8	0	2	0		14	4	4

Admittance to involuntary care
Intensivist 1: Quite oftenIntensivist 2: Always requires a doctor to make the assessment for psychosis or mania.
Number of responders: 14

	5	4	3	2	1		Total	Average	Median
Strongly agree	3	2	2	3	4	Strongly disagree	14	2,79	2,5
	21,43 %	14,28 %	14,29 %	21,43 %	28,57 %				
Total	3	2	2	3	4		14	2,79	2,5

Rapid sequence intubation and mechanical ventilation
Intensivist 1: Very rarely
Intensivist 2: TBI/ICH/aSAH, traumapatients, patients with a high risk for regurgitation of gastric fluids, severe hypoxemia, severe obesity, difficult airway.
Number of responders: 12

	5	4	3	2	1		Total	Average	Median
Strongly agree	7	4	0	1	0	Strongly disagree	12	4,42	5
	58,34%	33,33%	0%	8,33%	0%				
Total	7	4	0	1	0		12	4,42	5

Severe convulsions
Intensivist 1: Very rarely
Intensivist 2: If first line treatments are unsuccessful and patient is in active care, eg general anesthesia and intubation.
Number of responders: 14

	5	4	3	2	1		Total	Average	Median
Strongly agree	1	8	2	3	0	Strongly disagree	14	3,5	4
	7,14%	57,14%	14,29%	21,43%	0%				
Total	1	8	2	3	0		14	3,5	4

Need for IOT
Intensivist 1: No; does not need a physician
Number of responders: 13

	5	4	3	2	1		Total	Average	Median

Strongly agree	0	0	7	3	3	Strongly disagree	13	2,31	3
	0%	0%	53,84%	23,08%	23,08%				
Total	0	0	7	3	3		13	2,31	3

Acute psychological intervention for suicidal patient
Intensivist 1: Very rarely
Intensivist 2: To recognize and to intervene.
Number of responders: 14

	**5**	**4**	**3**	**2**	**1**		**Total**	**Average**	**Median**
Strongly agree	0	2	2	5	5	Strongly disagree	14	2,07	2
	0%	14,29%	14,29%	35,71%	35,71%				
Total	0	2	2	5	5		14	2,07	2

Trauma-care
Intensivist 1: Very rarely
Intensivist 2: Severe trauma patients.
Number of responders: 14

	**5**	**4**	**3**	**2**	**1**		**Total**	**Average**	**Median**
Strongly agree	2	4	5	2	1	Strongly disagree	14	3,29	3
	14,29%	28,57%	35,71%	14,29%	7,14%				
Total	2	4	5	2	1		14	3,29	3

Complicated inter-hospital transfers
Intensivist 1: Sometimes yes
Intensivist 2: Transfers by air, long-distance transfers in the critically ill (even intercontinental). Pediatric or neonatal patients. Any critically ill patients from hospital to hospital.
Number of responders: 14

	**5**	**4**	**3**	**2**	**1**		**Total**	**Average**	**Median**
Strongly agree	8	2	1	1	2	Strongly disagree	14	3,93	5
	57,14%	14,29%	7,14%	7,14%	14,29%				
Total	8	2	1	1	2		14	3,93	5

Carbon monoxide measurement
Intensivist 1: No; does not need a physician
Intensivist 2: ABG (depends on the education for paramedics)
Number of responders: 14

	**5**	**4**	**3**	**2**	**1**		**Total**	**Average**	**Median**
Strongly agree	0	3	2	1	8	Strongly disagree	14	2	1
	0%	21,43%	14,29%	7,14%	57,14%				
Total	0	3	2	1	8		14	2	1

Procedural sedation

Intensivist 1: Sometimes yes
Intensivist 2: Propofol sedation for any procedure. Sedation for elevated ICP.
Number of responders: 14

	5	4	3	2	1		Total	Average	Median
Strongly agree	2	6	2	1	3	Strongly disagree	14	3,21	4
	14,28%	42,86%	14,29%	7,14%	21,43%				
Total	2	6	2	1	3		14	3,21	4

Cardioversion
Intensivist 1: Sometimes yes
Intensivist 2: Propofol sedatiBasically always requires a doctor. In severe hemodynamical collapse a non-doctor can perform after consultation
Number of responders: 14

	5	4	3	2	1		Total	Average	Median
Strongly agree	5	7	2	0	0	Strongly disagree	14	4,21	4
	35,71%	50%	14,29%	0%	0%				
Total	5	7	2	0	0		14	4,21	4

Mass casualty incidents (etc. leaderships, Triage)
Intensivist 1: Yes
Intensivist 2: Any situation that requires deep understanding for hospital resources and resources on the field.
Number of responders: 14

	5	4	3	2	1		Total	Average	Median
Strongly agree	10	3	1	0	0	Strongly disagree	14	4,64	5
	71,43%	21,43%	7,14%	0%	0%				
Total	10	3	1	0	0		14	4,64	5

Tactical Emergency Medical Service (Police or Military lead special operations)
Intensivist 1: Yes
Intensivist 2:Any incident with a high risk for mass casualty or severe casualty.
Number of responders: 14

	5	4	3	2	1		Total	Average	Median
Strongly agree	4	5	2	2	1	Strongly disagree	14	3,64	4
	28,57%	35,71%	14,29%	14,29%	7,14%				
Total	4	5	2	2	1		14	3,64	4

Patients with sepsis and/or septic shock
Intensivist 1: Very rarely
Intensivist 2:Severe sepsis, need for vasoactive medication and life-saving fluid resiscitation. Obtaining samples and starting iv-antibiotics, especially for patients with long distance to hospital. Typical diseases meningitis, streptococcal sepsis, severe pneumonia.
Pediatrician 1: Important. likely and potentially life-saving in children. Most important = suspicion and start of fluid resuscitation and vasoconstrictors if shocked + to transport straight to a place where pediatric intensive care possible
Peditrcian 2: Patients with respiratory/circulatory failure, decreased conscious level. Monitoring (oxygenation, breathing, circulation, neurology). treatment: FiO2 100% oxygenation via nasal prongs/mask or intubation and mechanical ventilation in severe respiratory failure or unconsiuous patient.rapid iv/io access, fluids, vasoactives if needed.treatment of possible seizures (sepsis + meningitis?).
Pediatrician 3: fluid resuscitation
Number of responders: 14

	5	4	3	2	1		Total	Average	Median
Strongly agree	4	10	0	0	0	Strongly disagree	14	4,29	4
	28,57%	71,43%	0%	0%	0%				
Total	4	10	0	0	0		14	4,29	4

Vasoactives or other physician-staffed EMS level medication
Intensivist 1: Hardly ever
Intensivist 2: Severe sepsis, severe heart failure, maintaining CPP.
Pediatrician 1: Most important = suspicion and start of fluid resuscitation
Pediatrician 2: in cases of circulatory failure not responding to fluid boluses, signs of end-organ failure
Pediatrician 3: usually not needed
Number of responders: 14

	5	4	3	2	1		Total	Average	Median
Strongly agree	5	7	1	1	0	Strongly disagree	14	4,14	4
	35,72%	50%	7,14%	7,14%	0%				
Total	5	7	1	1	0		14	4,14	4

Pre-hospital antibiotic administration
Intensivist 1: Very rarely
Intensivist 2: Severe sepsis, transportation time to hospital over 1 hour. Physician should weight the benefits and harms for antibiotics considering diagnostics in hospital.
Pediatrician 1: May be considered after consultaion if long transport time (> 1-2 h)
Pediatrician 2: Not mandatory in most cases (distance less than one hour form hospital)
Pediatrician 3: depending on transporttime
Number of responders: 14

	5	4	3	2	1		Total	Average	Median
Strongly agree	2	2	3	4	3	Strongly disagree	14	2,71	2,5
	14,28 %	14,29 %	21,43 %	28,57 %	21,43 %				
Total	2	2	3	4	3		14	2,71	2,5

Arterial blood gas or other blood sample
Intensivist 1: Very rarely
Intensivist 2: Blood cultures before antibiotics. Directing treatment based on abg (ventilation, diagnostics etc.). Methanol intoxication and fomepizol. Carbonmonoxide inhalation and neurological symptoms.
Pediatrician 1: May be of use for diagnostic purposes (lactate), but should not delay start of treatment and transport
Pediatrician 2: arterial access may be challenging among critically ill children, blood samples could be taken from iv/io cannule
Number of responders: 13

	**5**	**4**	**3**	**2**	**1**		**Total**	**Average**	**Median**
Strongly agree	0	6	0	3	4	Strongly disagree	13	2,62	2
	0%	46,15%	0%	23,08%	30,77%				
Total	0	6	0	3	4		13	2,62	2

Meningitis
Intensivist 1: Rarely
Intensivist 2: Meningoccal (or other bacterial) meningitis with rapid onset, administration of steroid and antibiotics after blood cultures (CSF-sample not possible). Treatment of septic shock and ICP.
Pediatrician 1: As for sepsis / shock
Pediatrician 2: Monitoring: oxygenation, ventilation, neurology, blood glucose etc.Treatment of any signs of organ failure: oxygenation, respiratory support, circulatory support (fluid boluses, vasoactives), treatment of seizures
Number of responders: 14

	**5**	**4**	**3**	**2**	**1**		**Total**	**Average**	**Median**
Strongly agree	2	6	2	2	2	Strongly disagree	14	3,29	4
	14,28 %	42,86 %	14,28 %	14,29 %	14,29 %				
Total	2	6	2	2	2		14	3,29	4

Necrotizing fasciitis
Intensivist 1: Very rarely
Intensivist 2: Recognizing, blood cultures and antibiotics, treatment of septic shock. Directing patients to hospitals with plastic surgeons on call/HBOT.
Pediatrician 1: Rare in children, same observations than for sepsis apply
Pediatrician 2: Monitoring: signs of septic shock
Number of responders: 14

	**5**	**4**	**3**	**2**	**1**		**Total**	**Average**	**Median**
Strongly agree	3	4	1	3	3	Strongly disagree	14	3,07	3,5
	21,43%	28,57%	7,14%	21,43%	21,43%				
Total	3	4	1	3	3		14	3,07	3,5

Steroid administration
Intensivist 1: Hardly ever
Intensivist 2: In certain resuscitations, meningitis, anaphylaxis. In asthma and copd, although onset is slow

Pediatrician 1: Can wait until hospital in most cases, potentially useful in anaphylaxis if long transport time
Pediatrician 2: Iv administration if neede (asthma,anaphylaxis etc)
Number of responders: 14

	5	4	3	2	1		Total	Average	Median
Strongly agree	0	1	4	2	7	Strongly disagree	14	1,93	1,5
	0%	7,14%	28,57%	14,29%	50%				
Total	0	1	4	2	7		14	1,93	1,5

Severe acute lung injury
Intensivist 1: Very rarely
Intensivist 2: Smoke inhalation and lowered GCS. Viral or bacterial pneumonia with need for IMV. Severe hypoxia or hypercarbia in patients without previous or mild medical history, need for NMBA to facilitate IMV.
Pediatrician 1: Monitoring and treatment of oxygenation, ventilation and circulationtransport to hospital
Number of responders: 14

	5	4	3	2	1		Total	Average	Median
Strongly agree	2	5	3	3	1	Strongly disagree	14	3,29	3,5
	14,29%	35,71%	21,43%	21,43%	7,14%				
Total	2	5	3	3	1		14	3,29	3,5

Invasive monitoring
Intensivist 1: No
Intensivist 2: Arterial line and need for vasoactive medication
Pediatrician 1: depending on the length of the transport (not important in small children with 30-60 min transport time - blood pressure not as vital parameter as in adults)
Pediatrician 2: Insertion of arteria access may be difficult in out-of hospital enviroment.Saturation, etCO2 and non-invasive BP measurement is sufficient in most of the cases.
Pediatrician 3: usually not necessary
Number of responders: 14

	5	4	3	2	1		Total	Average	Median
Strongly agree	5	4	0	2	3	Strongly disagree	14	3,43	4
	35,71%	28,57%	0%	14,29%	21,43%				
Total	5	4	0	2	3		14	3,43	4

Management of severe heart failure
Intensivist 1: No
Intensivist 2: Instable patients eg. hemodynamical, respiratory or neurological instability. Difficult airway and/or need for surgical airway. Need for circulatory support eg. vasoactive medication.
Pediatrician 1: Quite important - more important would be to suspect this in children who often present with ambiguous signs and symptoms in case of heart failure, and rapidly start treatment (fluids - inotropy - minimise oxygen consumption)
Pediatrician 2: Oxygenation (FiO2 100%).Ventilatory support if conscious level drops or pulmonary oedema worsens; early tracheal intubation and mechanical ventilation, if expert help available.Cardiovascular monitoring, early vascular access.transport to PICU (paediatric cardiologist)

Pediatrician 3: usually not necessary
Number of responders: 14

	5	4	3	2	1		Total	Average	Median
Strongly agree	6	7	0	1	0	Strongly disagree	14	4,29	4
	42,86%	50%	0%	7,14%	0%				
Total	6	7	0	1	0		14	4,29	4

Pulmonary embolism treatment (etc. CPAP, thrombolysis)
Intensivist 1: Very rarely
Intensivist 2: Resuscitation, need for airway management, thrombolysis on site based on clinical status and echocardiography.
Pediatrician 1: PE very rare in children, thrombolysis not performed out of hospital in children.In contrast, use of CPAP for other breathing difficulties in children would be of vital importance
Pediatrician 2: Occurs extremely rarely in children,oxygenation, ventilatory support etc.
Gynecologist: agree
Number of responders: 14

	5	4	3	2	1		Total	Average	Median
Strongly agree	3	11	0	0	0	Strongly disagree	14	4,21	4
	21,43%	78,57%	0%	0%	0%				
Total	3	11	0	0	0		14	4,21	4

Status asthmaticus
Intensivist 1: Hardly ever
Intensivist 2: Resuscitation, need for airway management, thrombolysis on site based on clinical status and echocardiography.
Pediatrician 1: Vital importance
Pediatrician 2: Intubation in respiratory failure
Pediatrician 3: inhaled drugs
Number of responders: 14

	5	4	3	2	1		Total	Average	Median
Strongly agree	5	4	2	3	0	Strongly disagree	14	3,79	4
	35,71%	28,57%	14,29%	21,43%	0%				
Total	5	4	2	3	0		14	3,79	4

Administration of drugs only available for physician-staffed EMS unit (etc. certain bronchodilatators)
Intensivist 1: Very rarely
Intensivist 2: Resuscitation, severe obstruction and need for articial airway, severe obstruction and auto-PEEP, very difficult mechanical ventilation.
Pediatrician 1: Vital importance in children (racemic adrenaline, magnesium?)
Pediatrician 2: certain bronchodilatators and vasoactive treatment etc
Pediatrician 3: beta2-agonists are primary treatment in children
Number of responders: 14

	5	4	3	2	1		Total	Average	Median

Strongly agree	2	6	2	2	2	Strongly disagree	14	3,29	4
	14,28 %	42,86 %	14,28 %	14,29 %	14,29 %				
Total	2	6	2	2	2		14	3,29	4

Status epilepticus or other underlying neurological disease in need of anti-convulsive or other drugs only provided by physician-staffed EMS
Neurologist 1: Different anticonvulsive medications (up to general anesthesia if needed) are vital in ceasing the seizure. Safely and efficiently securing ABC. Possible underlying causes (e.g., hyponatremia) of convulsive activity can be screened and possible treated.
Neurologist 2: Does benefit from first- (benzodiazepines) and second phase(anti-epileptics)as early as possible, transportation to the hospital and if necessary, on a long transportation, also from sedation during the transport. There should always be able to check patients GCS and make an evaluation of care at hospital. I don't encourage to general anesthesia, first and second phase medication should be primary treatments to end seizures.
Pediatrician 1: Very important scenario. Prompt treatment of seizures prevents status epilepticus and, hence, neurological disabilities.
Pediatrician 2: ABCDE approach, oxygenation, intubation and ventilation of unconscious patient, treatment of convulsions, sedation, iv/io fluids
Pediatrician 3: only if still in status, abstain from RSI fro post convulsion/drug sleep
Number of responders: 14

	5	4	3	2	1		Total	Average	Median
Strongly agree	4	7	2	1	0	Strongly disagree	14	4	4
	28,57%	50%	14,29%	7,14%	0%				
Total	4	7	2	1	0		14	4	4

Status epilepticus or other neurological underlying disease in need of RSI
Neurologist 1: Different anticonvulsive medications (up to general anesthesia if needed) are vital in ceasing the seizure. Safely and efficiently securing ABC. Possible underlying causes (e.g., hyponatremia) of convulsive activity can be screened and possible treated.
Pediatrician 1: As above (paediatric airway can often be well maintained without intubation if personnel not comfortable with intubation of a child)
Pediatrician 2: ABCDE approach, oxygenation, intubation and ventilation of unconscious patient, treatment of convulsions, sedation, iv/io fluids
Pediatrician 3: only if still in status, abstain from RSI fro post convulsion/drug sleep
Number of responders: 14

	5	4	3	2	1		Total	Average	Median
Strongly agree	11	3	0	0	0	Strongly disagree	14	4,79	5
	78,57%	21,43%	0%	0%	0%				
Total	11	3	0	0	0		14	4,79	5

Severe meningitis/encephalitis and prehospital antibiotic administration
Neurologist 1: Quite theoretical, diagnosis rarely available before hospital
INeurologist 2: Hyperacute antibiotic treatment can be lifesaving and outcome can be improved by immediate treatment.
Neurologist 3: Does benefit
Pediatrician 1: relevant if > 1-2 h transport
Pediatrician 2: ABCDE approach, oxygenation, intubation and ventilation of unconscious patient, treatment of convulsions, sedation, iv/io fluids
Pediatrician 3: if long transportation time
Number of responders: 14

	5	4	3	2	1		Total	Average	Median
Strongly agree	4	4	2	3	1	Strongly disagree	14	3,5	4
	28,57%	28,57%	14,29%	21,43%	7,14%				
Total	4	4	2	3	1		14	3,5	4

Intracranial hypertension
Neurologist 1: Increased intracranial pressure (ICP) can be periodically treated with Mannitol/hypertonic saline, hyperventilation and proper sedation.Efficient treatment of hypotension and hypoxia is crucial in improving outcome.
Pediatrician 1: Role of EMS crucial (optimisation of ventilation, glycaemia,oxygenation, prompt transport)
Pediatrician 2: ABCDE approach, oxygenation, rapid intubation and ventilation, treatment of fever and convulsions, sedation, iv/io fluids, vasoactive medication
Number of responders: 14

	5	4	3	2	1		Total	Average	Median
Strongly agree	9	4	1	0	0	Strongly disagree	14	4,57	5
	64,29%	28,57%	7,14%	0%	0%				
Total	9	4	1	0	0		14	4,57	5

Intubation and ventilation of prematures and new-born babies
Pediatrician 1: Life-saving measure. Ventilation may be all that is needed.
Pediatrician 2: Neonatology and neonatology nurse is taken along to the sceneIntubation and ventilation of prematures and newborns is performed by the expertIf experts are not available, EMS physician will intubate and ventilate the infants
Pediatrician 3: ventilation
Gynecologist: agree
Number of responders: 14

	5	4	3	2	1		Total	Average	Median
Strongly agree	10	3	0	1	0	Strongly disagree	14	4,57	5
	71,43%	21,43%	0%	7,14%	0%				
Total	10	3	0	1	0		14	4,57	5

Surfactant administration
Pediatrician 1: Can normally wait until hospital (except in cases of long transport times)
Pediatrician 2: Neonatology and neonatology nurse is taken along to the sceneSurfactan administration is performed by the expert
Pediatrician 3: no
Gynecologist: agree
Number of responders: 14

	5	4	3	2	1		Total	Average	Median
Strongly agree	4	2	3	0	5	Strongly disagree	14	3	3
	28,57%	14,29%	21,43%	0%	35,71%				
Total	4	2	3	0	5		14	3	3

Pre-hospital caesarean section
Pediatrician: Can be life-saving
Gynecologist: agree
Number of responders: 14

	5	4	3	2	1		Total	Average	Median
Strongly agree	9	4	0	0	1	Strongly disagree	14	4,43	5
	64,29%	28,57%	0%	0%	7,14%				
Total	9	4	0	0	1		14	4,43	5

Pre-eclampsia and eclampsia treatments
Gynecologist: agree. Any emergency staff could start life-saving MgSo4-infusion in case on eclampsia under supervision of a physician (by phone for example). In case of pre-eclampsia there is usually time to transport the patient to hospital
Number of responders: 13

	5	4	3	2	1		Total	Average	Median
Strongly agree	5	6	1	1	0	Strongly disagree	13	4,15	4
	38,46%	46,16%	7,69%	7,69%	0%				
Total	5	6	1	1	0		13	4,15	4

Maternal rapid sequence intubation and ventilation
Gynecologist: agree
Number of responders: 14

	5	4	3	2	1		Total	Average	Median
Strongly agree	9	4	0	0	1	Strongly disagree	14	4,43	5
	64,29%	28,57%	0%	0%	7,14%				
Total	9	4	0	0	1		14	4,43	5

Resuscitation of newborn
Pediatrician 1: Life-saving measure. Ventilation may be all that is needed.
Pediatrician 2: Neonatology and neonatology nurse is taken along to the sceneResuscitation of prematures and newborns is performed by the expertIf experts are not available, EMS physician will resuscitate the newborn with the help of other staff
Pediatrician 3: yes
Gynecologist: agree
Number of responders: 14

	5	4	3	2	1		Total	Average	Median
Strongly agree	11	3	0	0	0	Strongly disagree	14	4,79	5
	78,57%	21,43%	0%	0%	0%				
Total	11	3	0	0	0		14	4,79	5

Pre-hospital childbirth of a premature, or special maneuvers in childbirth (etc. repositioning of fetus)
Pediatrician 1: Life-saving measure. Ventilation may be all that is needed.
Pediatrician 2: Neonatology and neonatology nurse is taken along to the sceneTreatment of prematures and newborns is performed by the expert
Gynecologist: agree. when physician is not available, any medical staff should attempt maneuvers under instructions by a physician eg by phone, because the situation must be handled abruptly
Number of responders: 14

	5	4	3	2	1		Total	Average	Median
Strongly agree	8	4	1	1	0	Strongly disagree	14	4,36	5
	57,14%	28,57%	7,15%	7,14%	0%				
Total	8	4	1	1	0		14	4,36	5

Severe bleeding
Pediatrician 1: In mother? NA.In case of out-of-hospital birth after placental ablation -> resuscitation of newborn, potentially fluid bolus to newborn; blood transfusion to the newborn after ablation could be considered if long transport time and if the child is responding to resuscitation
Gynecologist: agree. When a physician is not available shortly, any medical staff must start maneuvers promptly (uterine or aortic compression, medication under instructions)
Number of responders: 14

	5	4	3	2	1		Total	Average	Median
Strongly agree	3	9	2	0	0	Strongly disagree	14	4,07	4
	21,43%	64,28%	14,29%	0%	0%				
Total	3	9	2	0	0		14	4,07	4

Pre-hospital episiotomy
Gynecologist: Any medical staff should be able to perform under instructions -seldom needed in spontaneous dleiveries
Number of responders: 14

	5	4	3	2	1		Total	Average	Median
Strongly agree	4	2	1	4	3	Strongly disagree	14	3	2,5
	28,57%	14,29%	7,14%	28,57%	21,43%				

Total	4	2	1	4	3		14	3	2,5

HELP syndrome treatments
Gynecologist: MgSo4- infusion should be initiated by medical staff under instructions if a physician is not available shortly. Rarely needed so promtly, usually possible to transport to hospital first.
Number of responders: 14

	5	4	3	2	1		Total	Average	Median
Strongly agree	4	3	3	2	2	Strongly disagree	14	3,36	3,5
	28,57 %	21,43 %	21,43 %	14,28 %	14,29 %				
Total	4	3	3	2	2		14	3,36	3,5

Severe haemodynamic instability
Number of responders: 14

	5	4	3	2	1		Total	Average	Median
Strongly agree	9	3	1	0	1	Strongly disagree	14	4,36	5
	64,29%	21,43%	7,14%	0%	7,14%				
Total	9	3	1	0	1		14	4,36	5

Time benefit >15min with the use of helicopter unit, or rapid transportation with car unit
Number of responders: 14

	5	4	3	2	1		Total	Average	Median
Strongly agree	3	5	3	1	2	Strongly disagree	14	3,43	4
	21,43%	35,71%	21,43%	7,14%	14,29%				
Total	3	5	3	1	2		14	3,43	4

Pre-hospitally performed CT scan and prehospital thrombolysis
Number of responders: 14

	5	4	3	2	1		Total	Average	Median
Strongly agree	4	4	2	2	2	Strongly disagree	14	3,43	4
	28,57 %	28,57 %	14,28 %	14,29 %	14,29 %				
Total	4	4	2	2	2		14	3,43	4

Early thrombolysis
Cardiologist: Early thrombolysis is indicated and beneficial in case of ST-elevation MI if patient can not be transferred to angiography in 120 minutes. Early thrombosis is essential in patient with pulmonary embolism and hemodynamic compromise.
Number of responders: 14

	**5**	**4**	**3**	**2**	**1**		**Total**	**Average**	**Median**
Strongly agree	4	4	2	2	2	Strongly disagree	14	3,43	4
	28,57 %	28,57 %	14,28 %	14,29 %	14,29 %				
Total	4	4	2	2	2		14	3,43	4

Severe pulmonary embolism
Cardiologist: Early thrombosis is essential in patient with pulmonary embolism and hemodynamic compromise.
Number of responders: 14

	**5**	**4**	**3**	**2**	**1**		**Total**	**Average**	**Median**
Strongly agree	5	7	1	0	1	Strongly disagree	14	4,07	4
	35,72%	50%	7,14%	0%	7,14%				
Total	5	7	1	0	1		14	4,07	4

Severe AMI
Cardiologist: Patient should be diagnosed with ECG immediately and transferred to angiolab.
Number of responders: 14

	**5**	**4**	**3**	**2**	**1**		**Total**	**Average**	**Median**
Strongly agree	3	5	0	3	3	Strongly disagree	14	3,14	4
	21,43%	35,71%	0%	21,43%	21,43%				
Total	3	5	0	3	3		14	3,14	4

Cardioversion or drug administration that is only available for physician-staffed EMS unit
Number of responders: 14

	**5**	**4**	**3**	**2**	**1**		**Total**	**Average**	**Median**
Strongly agree	6	5	1	1	1	Strongly disagree	14	4	4
	42,86%	35,72%	7,14%	7,14%	7,14%				
Total	6	5	1	1	1		14	4	4

Benefit of HEMS transportation
Number of responders: 14

	5	4	3	2	1		Total	Average	Median
Strongly agree	5	4	2	2	1	Strongly disagree	14	3,71	4
	35,71%	28,57%	14,29%	14,29%	7,14%				
Total	5	4	2	2	1		14	3,71	4

Thoracic aneurysm
Number of responders: 14

	5	4	3	2	1		Total	Average	Median
Strongly agree	2	5	1	5	1	Strongly disagree	14	3,14	3,5
	14,29%	35,72%	7,14%	35,71%	7,14%				
Total	2	5	1	5	1		14	3,14	3,5

Cardiac arrest and difficult airway
Cardiologist: Benefit Score 8.Resuscitation on scene as soon as possible (compression, defibrillation, iv-lines, airway).Physician is needed on scene.
Number of responders: 14

	5	4	3	2	1		Total	Average	Median
Strongly agree	10	2	0	2	0	Strongly disagree	14	4,43	5
	71,43%	14,28%	0%	14,29%	0%				
Total	10	2	0	2	0		14	4,43	5

Cardiac arrest of a neonate, a newborn, a child or a pregnant patient
Number of responders: 14

	5	4	3	2	1		Total	Average	Median
Strongly agree	11	2	0	1	0	Strongly disagree	14	4,64	5
	78,57%	14,29%	0%	7,14%	0%				
Total	11	2	0	1	0		14	4,64	5

Thrombolysis administered during resuscitation
Cardiologist: Score 8. Diagnostic evaluation on scene. Is pulmonary embolism is suspected, thrombolysis might be lifesaving. Physicians attendance preferable (diagnosis, echo), but in some cases telephone consultation mihgt be enough because treatment can not be delayd.
Number of responders: 14

	5	4	3	2	1		Total	Average	Median
Strongly agree	3	4	3	4	0	Strongly disagree	14	3,43	3,5
	21,43%	28,57%	21,43%	28,57%	0%				
Total	3	4	3	4	0		14	3,43	3,5

Instability after ROSC requiring advanced skills etc. pacing, vasoactive or other specialized drugs

Number of responders: 14

	5	4	3	2	1		Total	Average	Median
Strongly agree	11	2	1	0	0	Strongly disagree	14	4,71	5
	78,57%	14,29%	7,14%	0%	0%				
Total	11	2	1	0	0		14	4,71	5

Cardiac arrest requiring dual or syncroneus defibrillation
Cardiologist: Score 8. Immediate dibrillation with semi-automatic defibrillarors can be life-saving. Physician on scene is not mandatory.
Number of responders: 14

	5	4	3	2	1		Total	Average	Median
Strongly agree	2	3	1	3	5	Strongly disagree	14	2,57	2
	14,29%	21,43%	7,14%	21,43%	35,71%				
Total	2	3	1	3	5		14	2,57	2

Patients after complex cardiac surgery
Cardiologist: Cardiac arrest after cardiac surgery can be due to tamponation and in case of arrest, prognosis is grave without immediate pericardiocentesis on scene.
Number of responders: 14

	5	4	3	2	1		Total	Average	Median
Strongly agree	3	2	4	2	3	Strongly disagree	14	3	3
	21,43 %	14,28 %	28,57 %	14,29 %	21,43 %				
Total	3	2	4	2	3		14	3	3

Cardiac Arrest should always be treated by physician
Cardiologist: No. Professional paramedics are cabable to start the treatment.
Number of responders: 14

	5	4	3	2	1		Total	Average	Median
Strongly agree	2	0	3	2	7	Strongly disagree	14	2,14	1,5
	14,28%	0%	21,43%	14,29%	50%				
Total	2	0	3	2	7		14	2,14	1,5

Free comments on HBS 8
Number of responders: 1

Responses
Key points:<br>- DEATH without care<br>- Good prognosis suspected<br>- other EMS staff not capable<br>BUT: 7 or 8 depends on the level of EMS care

HBS7

Thoracotomy
Traumatologist: minimal benefit
Number of responders: 14

	5	4	3	2	1		Total	Average	Median
Strongly agree	5	5	1	1	2	Strongly disagree	14	3,71	4
	35,72%	35,71%	7,14%	7,14%	14,29%				
Total	5	5	1	1	2		14	3,71	4

Rapid sequence intubation and ventilation
Traumatologist: minor benefit
Number of responders: 14

	5	4	3	2	1		Total	Average	Median
Strongly agree	6	3	3	1	1	Strongly disagree	14	3,86	4
	42,86%	21,43%	21,43%	7,14%	7,14%				
Total	6	3	3	1	1		14	3,86	4

Isolated severe trauma managed with simple maneuvers (etc. direct compression, tourniquet)
Traumatologist: minor benefit
Number of responders: 14

	5	4	3	2	1		Total	Average	Median
Strongly agree	7	4	0	2	1	Strongly disagree	14	4	4,5
	50%	28,57%	0%	14,29%	7,14%				
Total	7	4	0	2	1		14	4	4,5

Opening an obstructed airway or use of a supraglottic device and manual ventilation, or bag-mask ventilation
Traumatologist: great benefit
Number of responders: 14

	5	4	3	2	1		Total	Average	Median
Strongly agree	11	1	0	2	0	Strongly disagree	14	4,5	5
	78,57%	7,14%	0%	14,29%	0%				
Total	11	1	0	2	0		14	4,5	5

EMS (paramedic) -level medication
Traumatologist: minor benefit
Number of responders: 14

	5	4	3	2	1		Total	Average	Median
Strongly agree	0	2	4	3	5	Strongly disagree	14	2,21	2
	0%	14,29%	28,57%	21,43%	35,71%				
Total	0	2	4	3	5		14	2,21	2

Neurotrauma, or trauma needing EMS (paramedic) -level maneuvers
Traumatologist: minor benefit
Number of responders: 14

	5	4	3	2	1		Total	Average	Median
Strongly agree	1	5	1	4	3	Strongly disagree	14	2,79	2,5
	7,14%	35,72%	7,14%	28,57%	21,43%				
Total	1	5	1	4	3		14	2,79	2,5

Tranexamic acid administration
Traumatologist: great benefit
Number of responders: 14

	5	4	3	2	1		Total	Average	Median
Strongly agree	3	4	1	3	3	Strongly disagree	14	3,07	3,5
	21,43%	28,57%	7,14%	21,43%	21,43%				
Total	3	4	1	3	3		14	3,07	3,5

Needle thoracocentesis / pneumothorax
Traumatologist: great benefit
Number of responders: 14

	5	4	3	2	1		Total	Average	Median
Strongly agree	3	10	0	1	0	Strongly disagree	14	4,07	4
	21,43%	71,43%	0%	7,14%	0%				
Total	3	10	0	1	0		14	4,07	4

Blood transfusions
Traumatologist: minor benefit
Number of responders: 14

	5	4	3	2	1		Total	Average	Median
Strongly agree	5	6	1	2	0	Strongly disagree	14	4	4
	35,71%	42,86%	7,14%	14,29%	0%				
Total	5	6	1	2	0		14	4	4

Massive haemorrhage or severe trauma
Traumatologist: minor benefit

Number of responders: 14

	5	4	3	2	1		Total	Average	Median
Strongly agree	7	3	1	2	1	Strongly disagree	14	3,93	4,5
	50%	21,43%	7,14%	14,29%	7,14%				
Total	7	3	1	2	1		14	3,93	4,5

Need of helicopter unit to reach patient (remote area)
Traumatologist: great benefit
Number of responders: 14

	5	4	3	2	1		Total	Average	Median
Strongly agree	6	3	3	0	2	Strongly disagree	14	3,79	4
	42,86%	21,43%	21,43%	0%	14,28%				
Total	6	3	3	0	2		14	3,79	4

Drowning
Traumatologist: minor benefit
Number of responders: 14

	5	4	3	2	1		Total	Average	Median
Strongly agree	3	6	2	3	0	Strongly disagree	14	3,64	4
	21,43%	42,86%	14,28%	21,43%	0%				
Total	3	6	2	3	0		14	3,64	4

Unconscious patient
Traumatologist: minor benefit
Number of responders: 14

	5	4	3	2	1		Total	Average	Median
Strongly agree	1	5	4	4	0	Strongly disagree	14	3,21	3
	7,14%	35,72%	28,57%	28,57%	0%				
Total	1	5	4	4	0		14	3,21	3

Same as EMS Benefit score 8, when paramedics are qualified to perform
Traumatologist: minimal benefit
Intensivist 1: Rarely
Intensivist 2: Anything to save the patient as long as they have been trained, indications are vital and there is no physician present.Gynaecologist: agree(good prognosis if treated well)
Number of responders: 14

	5	4	3	2	1		Total	Average	Median
Strongly agree	3	2	5	2	2	Strongly disagree	14	3,14	3
	21,43%	14,28%	35,71%	14,29%	14,29%				
Total	3	2	5	2	2		14	3,14	3

Long-bone fracture reduction and stabilization
Traumatologist: great benefit
Number of responders: 14

	5	4	3	2	1		Total	Average	Median
Strongly agree	2	4	0	5	3	Strongly disagree	14	2,79	2
	14,29%	28,57%	0%	35,71%	21,43%				
Total	2	4	0	5	3		14	2,79	2

Transportation to ECMO
Traumatologist: minor benefit
Number of responders: 14

	5	4	3	2	1		Total	Average	Median
Strongly agree	3	3	3	3	2	Strongly disagree	14	3,14	3
	21,43 %	21,43 %	21,43 %	21,43 %	14,28 %				
Total	3	3	3	3	2		14	3,14	3

Prehospital GA
Traumatologist: minor benefit
Number of responders: 14

	5	4	3	2	1		Total	Average	Median
Strongly agree	5	1	6	1	1	Strongly disagree	14	3,57	3
	35,72%	7,14%	42,86%	7,14%	7,14%				
Total	5	1	6	1	1		14	3,57	3

Antidotes
Intensivist 1: Sometimes yes
Intensivist 2: Glucose for insulin intoxications, atropine for organophosphate poisonings
Pediatrician 1: Rare in children, naloxone could be life-saving in opioid intoxication in adolescents in the same way as in adults
Pediatrician 2: After consultation of "national poisoning center" depending of the poison and/or severity of poisoning and whether the antidote is immediately available.Call for hospital to inform about the need of antidote
Pediatrician 3 :if cause known and available and long transportation
Number of responders: 14

	5	4	3	2	1		Total	Average	Median
Strongly agree	1	5	3	2	3	Strongly disagree	14	2,93	3
	7,14%	35,71%	21,43%	14,29%	21,43%				
Total	1	5	3	2	3		14	2,93	3

Liver failure management
Intensivist 1: Very rarely
Intensivist 2: Difficult to treat prehospitally and often have a bad prognosis. ALF due to acetaminophen and acetylcysteine infusion perhaps

Pediatrician 1: Normally not needed in prehospital setting unless > 4-6h transport
Pediatrician 2: ABCDE assessment, oxygenation, intubation and ventilation of unconscious patient, fluids via iv/io-route for hypovolemic patient.Transport to the hospital
Number of responders: 14

	5	4	3	2	1		Total	Average	Median
Strongly agree	0	0	3	1	10	Strongly disagree	14	1,5	1
	0%	0%	21,43%	7,14%	71,43%				
Total	0	0	3	1	10		14	1,5	1

Glucose administration
Intensivist 1: Sometimes yes
Intensivist 2: In hypoglycemia or as part of glucose-insulin-infusion to treat hyperkalemia.
Pediatrician 1:Vital importance in all age groups
Pediatrician 2:po/iv/io for hypoglycemic patients
Pediatrician 3: yes
Number of responders: 14

	5	4	3	2	1		Total	Average	Median
Strongly agree	3	5	1	2	3	Strongly disagree	14	3,21	4
	21,43%	35,71%	7,14%	14,29%	21,43%				
Total	3	5	1	2	3		14	3,21	4

Sedation
Intensivist 1: Very rarely
Intensivist 2: Postresuscitation care, elevated ICP.
Pediatrician 1:Rarely needed in children except for cases needing intubation
Pediatrician 2:ABCDE assessment, oxygenation, intubation and ventilation of unconscious patient, iv/io-route for medication, sedation if needed (s-ketamine, opioids)
Number of responders: 14

	5	4	3	2	1		Total	Average	Median
Strongly agree	1	4	2	4	3	Strongly disagree	14	2,71	2,5
	7,14%	28,57%	14,29%	28,57%	21,43%				
Total	1	4	2	4	3		14	2,71	2,5

Preventive maneuvers (etc. suicidal patients and prevent to jump from high)
Intensivist: Sometimes yes
Pediatrician: Ad in adults
Number of responders: 14

	5	4	3	2	1		Total	Average	Median
Strongly agree	1	4	4	1	4	Strongly disagree	14	2,79	3
	7,15%	28,57%	28,57%	7,14%	28,57%				
Total	1	4	4	1	4		14	2,79	3

Cardioversion or cardiac pacing
Intensivist 1: Very rarely

Intensivist 2: Postresuscitation care, elevated ICP.
Pediatrician: As in adults, but rare in children
Number of responders: 14

	5	4	3	2	1		Total	Average	Median
Strongly agree	6	5	2	0	1	Strongly disagree	14	4,07	4
	42,86%	35,71%	14,29%	0%	7,14%				
Total	6	5	2	0	1		14	4,07	4

Severe hypoglycemia
Intensivist 1: Sometimes yes
Intensivist 2: Hypoglycemia from any cause, especially children and insulin poisonings
Number of responders: 14

	5	4	3	2	1		Total	Average	Median
Strongly agree	6	3	1	2	2	Strongly disagree	14	3,64	4
	42,86%	21,43%	7,14%	14,28%	14,29%				
Total	6	3	1	2	2		14	3,64	4

Anafylactic shock
Intensivist 1: Sometimes yes
Intensivist 2: Anaphylaxis causing severe bronchus obstruction, airway obstruction or hemodynamic collapse that is relieved with epinephrine and other medications.
Number of responders: 14

	5	4	3	2	1		Total	Average	Median
Strongly agree	9	4	1	0	0	Strongly disagree	14	4,57	5
	64,29%	28,57%	7,14%	0%	0%				
Total	9	4	1	0	0		14	4,57	5

Basic or advanced airway management
Intensivist 1: Sometimes yes
Intensivist 2: Supraglottic devices for airway management in patients without the ability to maintain open airway.
Number of responders: 14

	5	4	3	2	1		Total	Average	Median
Strongly agree	4	8	2	0	0	Strongly disagree	14	4,14	4
	28,57%	57,14%	14,29%	0%	0%				
Total	4	8	2	0	0		14	4,14	4

Haemorrhage control
Intensivist 1: Yes
Intensivist 2:Applying a tourniquet or pressure on severely bleeding wounds (amputation, crush injuries, penetrating injuries). Starting an iv-line, administering tranexamic acid.
Number of responders: 14

	5	4	3	2	1		Total	Average	Median

Strongly agree	9	4	1	0	0	Strongly disagree	14	4,57	5
	64,29%	28,57%	7,14%	0%	0%				
Total	9	4	1	0	0		14	4,57	5

Circulatory support
Intensivist 1: Sometimes yes
Intensivist 2: In hemodynamically instable bleeding patients. In severe septic shock and need for transportation.
Number of responders: 14

	5	4	3	2	1		Total	Average	Median
Strongly agree	3	7	3	1	0	Strongly disagree	14	3,86	4
	21,43%	50%	21,43%	7,14%	0%				
Total	3	7	3	1	0		14	3,86	4

Mechanical CPR
Intensivist 1: Sometimes yes
Intensivist 2: Hypothermic patients with circulatory collapse (drowning victims etc), young patients with prolonged cpr and a treatable cause for cardiac arrest (STEMI, pulmonary embolism)
Number of responders: 14

	5	4	3	2	1		Total	Average	Median
Strongly agree	2	6	2	2	2	Strongly disagree	14	3,29	4
	14,28 %	42,86 %	14,28 %	14,29 %	14,29 %				
Total	2	6	2	2	2		14	3,29	4

Antibiotics administration
Intensivist 1: Very rarely
Intensivist 2: Long transportation time in sepsis
Pediatrician: Not mandatory in most cases (distance less than one hour form hospital)
Number of responders: 14

	5	4	3	2	1		Total	Average	Median
Strongly agree	2	2	3	5	2	Strongly disagree	14	2,79	2,5
	14,28 %	14,29 %	21,43 %	35,71 %	14,29 %				
Total	2	2	3	5	2		14	2,79	2,5

Moderate septic shock
Intensivist 1: No
Intensivist 2: Long transportation time in sepsis. Need for fluid resuscitation/hemodynamic collapse and/or respiratory failure.
Pediatrician:Patients with the risk of respiratory/circulatory failure, decreased conscious level.Monitoring (oxygenation, breathing, circulation, neurology) treatment: O2 100% oxygenation via nasal prongs/mask. readiness for intubation and mechanical ventilation in progressive severe respiratory failure or unconsiuous patient.rapid iv/io access, fluids, vasoactives if neededtreatment of possible seizures (sepsis + meningitis?)

Number of responders: 14

	5	4	3	2	1		Total	Average	Median
Strongly agree	1	4	4	3	2	Strongly disagree	14	2,93	3
	7,14%	28,57%	28,57%	21,43%	14,29%				
Total	1	4	4	3	2		14	2,93	3

Airway management with supraglottic device and mask ventilation, or bag-mask ventilation or non-invasive ventilation

Intensivist 1: Rarely
Intensivist 2: Severe respiratory failure or inability to maintain open airway for any reason.
Pediatrician 1: Very important in children - can often lead to no need for intubation
Pediatrician 2:in cases of progressive respiratory failure

Number of responders: 14

	5	4	3	2	1		Total	Average	Median
Strongly agree	2	5	2	4	1	Strongly disagree	14	3,21	3,5
	14,29%	35,71%	14,29%	28,57%	7,14%				
Total	2	5	2	4	1		14	3,21	3,5

Severe sepsis

Intensivist 1: Very rarely
Intensivist 2: Sepsis in previously healthy/quite healthy and need for hemodynamic support. Long transportation time -> antibiotics
Pediatrician: Patients with respiratory/circulatory failure, decreased conscious level.Monitoring (oxygenation, breathing, circulation, neurology) treatment: FiO2 100% oxygenation via nasal prongs/mask or intubation and mechanical ventilation in severe respiratory failure or unconsiuous.ntrapid iv/io access, fluids, vasoactives if needed.treatment of possible seizures (sepsis + meningitis?)

Number of responders: 14

	5	4	3	2	1		Total	Average	Median
Strongly agree	5	4	1	2	2	Strongly disagree	14	3,57	4
	35,71%	28,57%	7,14%	14,29%	14,29%				
Total	5	4	1	2	2		14	3,57	4

Inotropes

Intensivist 1: Very rarely
Intensivist 2: Epinephrine in post-resuscitation
Peditarician 1:Useful if e.g. septic shock and long transport time
Pediatrician 2: in cases with circulatory failure not responding to fluid boluses

Number of responders: 14

	5	4	3	2	1		Total	Average	Median
Strongly agree	6	3	1	3	1	Strongly disagree	14	3,71	4
	42,86%	21,43%	7,14%	21,43%	7,14%				
Total	6	3	1	3	1		14	3,71	4

ABC (airway, breathing, circulation) –support
Intensivist 1: Rarely
Intensivist 2: If severe compromise in any ABC, rapid intervention.
Pediatrician 1: Mandatory approach in all children
Pediatrician 2: Evaluation and monitoring and support necessary for all patients who have signs of organ failure
Pediatrician 3: Yes
Number of responders: 14

	5	4	3	2	1		Total	Average	Median
Strongly agree	3	6	1	3	1	Strongly disagree	14	3,5	4
	21,43%	42,86%	7,14%	21,43%	7,14%				
Total	3	6	1	3	1		14	3,5	4

Meningitis
Intensivist 1: Very rarely
Intensivist 2:Sepsis in previously healthy/quite healthy and need for hemodynamic support. Long transportation time -> antibiotics. Need for IMV and controlling ICP.
Pediatrician: Monitoring: oxygenation, ventilation, neurology, blood glucose etc.Treatment of any signs of organ failure: oxygenation, respiratory support, circulatory support (fluid boluses, vasoactives), treatment of seizures
Number of responders: 14

	5	4	3	2	1		Total	Average	Median
Strongly agree	3	2	1	5	3	Strongly disagree	14	2,79	2
	21,43%	14,29%	7,14%	35,71%	21,43%				
Total	3	2	1	5	3		14	2,79	2

Acute lung injury
Intensivist 1: Very rarely
Intensivist 2:Severe hypoxia or hypercarbia in patients without severe chronic pulmonary condition.
Pediatrician: Monitoring and treatment of oxygenation, ventilation and circulation, transport to hospital
Number of responders: 14

	5	4	3	2	1		Total	Average	Median
Strongly agree	2	4	1	5	2	Strongly disagree	14	2,93	2,5
	14,29%	28,57%	7,14%	35,71%	14,29%				
Total	2	4	1	5	2		14	2,93	2,5

Pneumothorax
Intensivist 1: Very rarely
Intensivist 2: No hemodynamical instability, mild to moderate desaturation, tension pneumothx and needle thoracosentesis.
Pediatrician 1: The most important would be to suspect heart failure and to transport directly to a centre with pediatric cardiologic experience where this can be treated. Possibly important to start fluids, inotropy, minimise oxygen consumption.
Pediatrician 2:Continuous monitoring of oxygenation, breathing, ventilationStaff prepared to ask for help to insert chest drain if signs of respiratory failure
Number of responders: 14

	5	4	3	2	1		Total	Average	Median
Strongly agree	3	6	2	1	2	Strongly disagree	14	3,5	4
	21,43%	42,86%	14,28%	7,14%	14,29%				
Total	3	6	2	1	2		14	3,5	4

Facial injuries
Intensivist 1: Very rarely
Intensivist 2: Need for managing airway (in severe cases requires physician) without ETT. Obstructing aspiration of blood by positioning and suction.
Number of responders: 14

	5	4	3	2	1		Total	Average	Median
Strongly agree	1	7	3	1	2	Strongly disagree	14	3,29	4
	7,14%	50%	21,43%	7,14%	14,29%				
Total	1	7	3	1	2		14	3,29	4

Asthma
Intensivist 1: Hardly ever
Intensivist 2: Severe obstruction, need for bronchodilatory medication of all kind. Sedation and monitoring
Pediatrician 1: common in children, but rarely life-threatening before reaching hospital
Pediatrician 2:Continuous monitoring of oxygenation, breathing, ventilation,oxygenation,medication: inhaled salbutamol, i.m. adrenalin in life-threatening airway obstruction,iv access: corticosteroid
Number of responders: 14

	5	4	3	2	1		Total	Average	Median
Strongly agree	0	8	1	4	1	Strongly disagree	14	3,14	4
	0%	57,14%	7,15%	28,57%	7,14%				
Total	0	8	1	4	1		14	3,14	4

Anaphylactic reaction
Intensivist 1: Sometimes
Intensivist 2: Severe anaphylaxis, need for epinephrine and even epinephrine infusion. Open airway.
Number of responders: 13

	5	4	3	2	1		Total	Average	Median
Strongly agree	5	4	0	4	0	Strongly disagree	13	3,77	4
	38,46%	30,77%	0%	30,77%	0%				
Total	5	4	0	4	0		13	3,77	4

Non-invasive ventilation
Intensivist 1: Sometimes
Intensivist 2: Cardiac failure, acute respiratory failure of any reason. COPD exacerbation.
Pediatrician 1: could be life-saving in infants, but probably not performed in the prehospital setting due to lack of equipment for neonates/ infants?
Pediatrician 2: Not indicated in otherwise healthy children. Usable among children wo have NIV treatment at home; they could be transported to hospital using their own device
Pediatrician 3: usually not necessary
Number of responders: 14

	5	4	3	2	1		Total	Average	Median
Strongly agree	2	4	0	8	0	Strongly disagree	14	3	2
	14,29%	28,57%	0%	57,14%	0%				
Total	2	4	0	8	0		14	3	2

Obstruction release with EMS (paramedic) level medication
Intensivist 1: Rarely
Intensivist 2: Desaturation, recurrent obstuction, need for sedative agents.
Number of responders: 14

	5	4	3	2	1		Total	Average	Median
Strongly agree	0	6	1	4	3	Strongly disagree	14	2,71	2,5
	0%	42,86%	7,14%	28,57%	21,43%				
Total	0	6	1	4	3		14	2,71	2,5

Airway management excluding RSI (etc. supraglottic device, release of foreign material or bag-mask ventilation)
Intensivist 1: Sometimes
Intensivist 2: Other indications than pulseless patient for airway management.
Number of responders: 14

	5	4	3	2	1		Total	Average	Median
Strongly agree	3	5	3	2	1	Strongly disagree	14	3,5	4
	21,43%	35,71%	21,43%	14,29%	7,14%				
Total	3	5	3	2	1		14	3,5	4

Rapid or long distance helicopter transportation of dyspneic patient
Intensivist 1: Very rarely
Intensivist 2: If there is no qualified physician available for transportation
Number of responders: 14

	5	4	3	2	1		Total	Average	Median
Strongly agree	1	2	4	6	1	Strongly disagree	14	2,71	2,5
	7,14%	14,29%	28,57%	42,86%	7,14%				
Total	1	2	4	6	1		14	2,71	2,5

Neurological disease in need of rapid sequence intubation
Neurologist 1: In cases with impaired consciousness (e.g., due to traumatic brain injury (TBI), ICH, acute hydrocephalus, aSAH), rapid intubation by an experienced physician is vital. Intubation by unexperienced professionals can be detrimental (e.g., in TBI can results in iatrogenic increase in intracranial pressure).
Neurologist 2: bacillary obstruction?
Number of responders: 14

	5	4	3	2	1		Total	Average	Median
Strongly agree	7	2	2	1	2	Strongly disagree	14	3,79	4,5
	50%	14,28%	14,29%	7,14%	14,29%				
Total	7	2	2	1	2		14	3,79	4,5

Status epilepticus and administration of first, or second line treatments (drugs provided by EMS unit, etc. bentsodiazepines or phosphenytoin)
Neurologist 1: In cases of protracted seizures, first and second line drugs are key in avoiding the development of status epilepticus. Without proper medication, the outcome can be extremely poor.
Neurologist 2: Extremly important
Number of responders: 14

	5	4	3	2	1		Total	Average	Median
Strongly agree	4	8	1	0	1	Strongly disagree	14	4	4
	28,57%	57,14%	7,15%	0%	7,14%				
Total	4	8	1	0	1		14	4	4

Treatment of hypoglycemia induced coma or seizures
Neurologist 1: The administration of thiamine is recommended.Neurologist 2: Extremly important

Pediatrician 1: Very important to diagnose and treat, prehospital treatment may prevent neurological sequelae
Pediatrician 2: ABCDE approach, oxygenation, intubation and ventilation of patient, treatment of hypoglycemia (10% glucose) and convulsions (benzodiat), sedation, iv/io fluids
Pediatrician 3: yes
Number of responders: 14

	5	4	3	2	1		Total	Average	Median
Strongly agree	8	3	2	1	0	Strongly disagree	14	4,29	5
	57,14%	21,43%	14,29%	7,14%	0%				
Total	8	3	2	1	0		14	4,29	5

Treatment of hypoventilation and airway control with EMS maneuvers (etc. bag mask, or supraglottic device)
Neurologist 1: Even short periods of hypoxemia are detrimental for brain injury and hemorrhage patients and can significantly reduce the odds of favorable outcome.
Neurologist 2: Extremly important
Pediatrician 1: Often all that is needed for airway control in children, very important
Pediatrician 2: ABCDE approach, oxygenation, BM-ventilation, SGD assesment and ventilation of patient, measurement of saturation etc
Pediatrician 3: yes
Number of responders: 14

	5	4	3	2	1		Total	Average	Median
Strongly agree	2	9	1	1	1	Strongly disagree	14	3,71	4
	14,29%	64,29%	7,14%	7,14%	7,14%				
Total	2	9	1	1	1		14	3,71	4

Child above 13-year-old with status epilepticus
Neurologist 1: Important drug administration
Pediatrician 1: Not different from a child under 13 y?
Pediatrician 2: treated as adults
Pediatrician 3:treat according to protocol
Number of responders: 14

	5	4	3	2	1		Total	Average	Median
Strongly agree	4	5	1	2	2	Strongly disagree	14	3,5	4
	28,57%	35,71%	7,14%	14,29%	14,29%				
Total	4	5	1	2	2		14	3,5	4

Pre-hospital complicated childbirth
Gynaecologist:agree(good prognosis if treated well)
Number of responders: 14

	5	4	3	2	1		Total	Average	Median
Strongly agree	7	4	0	1	2	Strongly disagree	14	3,93	4,5
	50%	28,57%	0%	7,14%	14,29%				
Total	7	4	0	1	2		14	3,93	4,5

Pre-eclampsia and eclampsia treatments
Gynaecologist:in case of eclampsia, treatment outside the hospital improves the prognosis and the prognosis is often good.In case of pre-eclampsia, transport to hospital before treatment usually is ok
Number of responders: 14

	5	4	3	2	1		Total	Average	Median
Strongly agree	5	4	2	2	1	Strongly disagree	14	3,71	4
	35,71%	28,57%	14,29%	14,29%	7,14%				
Total	5	4	2	2	1		14	3,71	4

Minor heamorrhage
Gynaecologist: the patient would not die in minor hemorrhage, but the prognosis improves if treated already on field
Number of responders: 13

	5	4	3	2	1		Total	Average	Median
Strongly agree	1	1	1	3	7	Strongly disagree	13	1,92	1
	7,69%	7,69%	7,69%	23,08%	53,85%				
Total	1	1	1	3	7		13	1,92	1

Haemorrhage controlling maneuvers (etc. aortic compression)
Gynaecologist: agree
Number of responders: 14

	5	4	3	2	1		Total	Average	Median
Strongly agree	3	7	3	0	1	Strongly disagree	14	3,79	4
	21,43%	50%	21,43%	0%	7,14%				
Total	3	7	3	0	1		14	3,79	4

Rapid transportation
Gynaecologist: agree
Number of responders: 14

	5	4	3	2	1		Total	Average	Median
Strongly agree	5	3	1	3	2	Strongly disagree	14	3,43	4
	35,71%	21,43%	7,14%	21,43%	14,29%				
Total	5	3	1	3	2		14	3,43	4

Uteral rupture treatments
Gynaecologist: Only general maneuvers to control bleeding aree possible outside the hospital. Diagnosis difficult to make outside the hospital .Controlling bleedind /infusion improves maternal survival/prognosis, but fetal prognosis may be poor
Number of responders: 14

	5	4	3	2	1		Total	Average	Median
Strongly agree	1	3	5	3	2	Strongly disagree	14	2,86	3
	7,14%	21,43%	35,71%	21,43%	14,29%				
Total	1	3	5	3	2		14	2,86	3

Bag-mask ventilation and CPR of a newborn
Gynaecologist: agree
Number of responders: 14

	5	4	3	2	1		Total	Average	Median
Strongly agree	11	2	0	1	0	Strongly disagree	14	4,64	5
	78,57%	14,29%	0%	7,14%	0%				
Total	11	2	0	1	0		14	4,64	5

Resuscitation of a pregnant patient
Gynaecologist: agree
Number of responders: 14

	5	4	3	2	1		Total	Average	Median
Strongly agree	9	2	3	0	0	Strongly disagree	14	4,43	5
	64,28%	14,29%	21,43%	0%	0%				
Total	9	2	3	0	0		14	4,43	5

Time benefit of rapid transportation
Gynaecologist: agree
Number of responders: 14

	5	4	3	2	1		Total	Average	Median
Strongly agree	4	3	3	2	2	Strongly disagree	14	3,36	3,5
	28,57 %	21,43 %	21,43 %	14,28 %	14,29 %				
Total	4	3	3	2	2		14	3,36	3,5

Prehospital CT and thrombolysis
Number of responders: 14

	5	4	3	2	1		Total	Average	Median
Strongly agree	3	2	3	3	3	Strongly disagree	14	2,93	3
	21,43 %	14,28 %	21,43 %	21,43 %	21,43 %				
Total	3	2	3	3	3		14	2,93	3

Spontaneous pneumothorax
Number of responders: 14

	5	4	3	2	1		Total	Average	Median
Strongly agree	0	2	4	5	3	Strongly disagree	14	2,36	2
	0%	14,29%	28,57%	35,71%	21,43%				
Total	0	2	4	5	3		14	2,36	2

Transportation to PCI within 30min
Cardiologist: In STEMI the wire crossing of thrombus should be done in 90 minutes from diagnosis.
Number of responders: 14

	5	4	3	2	1		Total	Average	Median
Strongly agree	2	6	2	2	2	Strongly disagree	14	3,29	4
	14,28 %	42,86 %	14,28 %	14,29 %	14,29 %				
Total	2	6	2	2	2		14	3,29	4

Thrombolysis
Number of responders: 14

	5	4	3	2	1		Total	Average	Median
Strongly agree	1	6	3	2	2	Strongly disagree	14	3,14	3,5
	7,14%	42,86%	21,43%	14,28%	14,29%				
Total	1	6	3	2	2		14	3,14	3,5

Benefit of helicopter unit to reach or transport a patient
Number of responders: 14

	5	4	3	2	1		Total	Average	Median
Strongly agree	2	6	3	1	2	Strongly disagree	14	3,36	4
	14,28%	42,86%	21,43%	7,14%	14,29%				
Total	2	6	3	1	2		14	3,36	4

EMS level cardioversion or drug administration
Number of responders: 13

	5	4	3	2	1		Total	Average	Median
Strongly agree	1	7	2	2	1	Strongly disagree	13	3,38	4
	7,69%	53,85%	15,39%	15,38%	7,69%				
Total	1	7	2	2	1		13	3,38	4

Oxygen or fluid administration
Number of responders: 14

	5	4	3	2	1		Total	Average	Median
Strongly agree	1	1	4	7	1	Strongly disagree	14	2,57	2
	7,15%	7,14%	28,57%	50%	7,14%				
Total	1	1	4	7	1		14	2,57	2

Aortic dissection
Number of responders: 14

	5	4	3	2	1		Total	Average	Median
Strongly agree	2	2	2	5	3	Strongly disagree	14	2,64	2
	14,28%	14,29%	14,29%	35,71%	21,43%				
Total	2	2	2	5	3		14	2,64	2

Chest drain
Number of responders: 14

	5	4	3	2	1		Total	Average	Median
Strongly agree	3	3	3	2	3	Strongly disagree	14	3,07	3
	21,43%	21,43%	21,43%	14,28%	21,43%				
Total	3	3	3	2	3		14	3,07	3

Overall resuscitation maneuvers (ALS, BLS) and Post-ROSC treatments
Number of responders: 14

	5	4	3	2	1		Total	Average	Median
Strongly agree	3	6	4	0	1	Strongly disagree	14	3,71	4
	21,43%	42,86%	28,57%	0%	7,14%				

Total	3	6	4	0	1		14	3,71	4

Cardiac arrest requiring transfer to ECMO or bypass
Number of responders: 14

	5	4	3	2	1		Total	Average	Median
Strongly agree	3	7	2	2	0	Strongly disagree	14	3,79	4
	21,43%	50%	14,28%	14,29%	0%				
Total	3	7	2	2	0		14	3,79	4

Cardiac arrest and blocked airway
Number of responders: 13

	5	4	3	2	1		Total	Average	Median
Strongly agree	8	4	1	0	0	Strongly disagree	13	4,54	5
	61,54%	30,77%	7,69%	0%	0%				
Total	8	4	1	0	0		13	4,54	5

Hypovolemic cardiac arrest (etc. need of blood products)
Number of responders: 14

	5	4	3	2	1		Total	Average	Median
Strongly agree	5	7	0	1	1	Strongly disagree	14	4	4
	35,72%	50%	0%	7,14%	7,14%				
Total	5	7	0	1	1		14	4	4

Cardiac arrest due to anaphylaxis
Number of responders: 14

	5	4	3	2	1		Total	Average	Median
Strongly agree	8	4	1	0	1	Strongly disagree	14	4,29	5
	57,14%	28,57%	7,15%	0%	7,14%				
Total	8	4	1	0	1		14	4,29	5

Cardiac arrest and pneumothorax
Number of responders: 14

	5	4	3	2	1		Total	Average	Median
Strongly agree	10	3	0	0	1	Strongly disagree	14	4,5	5
	71,43%	21,43%	0%	0%	7,14%				
Total	10	3	0	0	1		14	4,5	5

Need of helicopter unit to reach a cardiac arrest patient
Number of responders: 14

	5	4	3	2	1		Total	Average	Median

Strongly agree	5	4	1	2	2	Strongly disagree	14	3,57	4
	35,71%	28,57%	7,14%	14,29%	14,29%				
Total	5	4	1	2	2		14	3,57	4

Free comments on HBS 7
Number of responders: 2

Responses
7 or 8 depends on the level of EMS care
A lot of the mentioned examples will need a physician at the scene.

HBS6

Patient suffering hypovolemia, hypotension or/and bleeding
Traumatologist: maybe benefit
Number of responders: 15

	5	4	3	2	1		Total	Average	Median
Strongly agree	8	6	1	0	0	Strongly disagree	15	4,47	5
	53,33%	40%	6,67%	0%	0%				
Total	8	6	1	0	0		15	4,47	5

Hypoxia
Traumatologist: maybe benefit
Number of responders: 15

	5	4	3	2	1		Total	Average	Median
Strongly agree	5	8	1	1	0	Strongly disagree	15	4,13	4
	33,33%	53,33%	6,67%	6,67%	0%				
Total	5	8	1	1	0		15	4,13	4

Rapid sequence intubation and ventilation
Traumatologist: maybe benefit
Intensivist 1: Sometimes yes
Intensivist 2: Intoxications. TBI etc. ARF.
Number of responders: 14

	5	4	3	2	1		Total	Average	Median
Strongly agree	6	5	2	1	0	Strongly disagree	14	4,14	4
	42,86%	35,71%	14,29%	7,14%	0%				
Total	6	5	2	1	0		14	4,14	4

Pleural decompression
Traumatologist: great benefit
Number of responders: 15

	5	4	3	2	1		Total	Average	Median
Strongly agree	8	6	0	1	0	Strongly disagree	15	4,4	5
	53,33%	40%	0%	6,67%	0%				
Total	8	6	0	1	0		15	4,4	5

Transfusion of blood products
Traumatologist: great benefit
Number of responders: 15

	5	4	3	2	1		Total	Average	Median
Strongly agree	8	6	0	1	0	Strongly disagree	15	4,4	5
	53,33%	40%	0%	6,67%	0%				
Total	8	6	0	1	0		15	4,4	5

Pericardiocentesis
Traumatologist: great benefit
Number of responders: 15

	5	4	3	2	1		Total	Average	Median
Strongly agree	5	1	5	3	1	Strongly disagree	15	3,4	3
	33,33%	6,67%	33,33%	20%	6,67%				
Total	5	1	5	3	1		15	3,4	3

Reduction and stabilization of fractures or luxations
Traumatologist: maybe benefit
Intensivist 1: Rarely
Intensivist 2: Dislocated fractures, especially ankle
Number of responders: 15

	5	4	3	2	1		Total	Average	Median
Strongly agree	4	7	2	1	1	Strongly disagree	15	3,8	4
	26,67%	46,66%	13,33%	6,67%	6,67%				
Total	4	7	2	1	1		15	3,8	4

Vasoactive administration
Traumatologist: great benefit
Number of responders: 15

	5	4	3	2	1		Total	Average	Median
Strongly agree	5	7	3	0	0	Strongly disagree	15	4,13	4
	33,33%	46,67%	20%	0%	0%				
Total	5	7	3	0	0		15	4,13	4

Triage and patient selection to dedicated center and rapid transportation
Traumatologist: great benefit
Intensivist 1: Yes
Intensivist 2: Recognizing red patients
Neurologist 1:In Finland, only university hospitals have neurosurgical services that can treat emergency cases. If a patient potentially has a neurosurgically treatable condition, although the prognosis seem unfavorable, the initial place of assessment/treatment should be the closest university hospital if logistically possible.
Neurologist 2: If patient is already a mRS >3 (Modified Rankin Scale for Neurologic Disability) before the prehospital incident, and in need of assistance for everyday-life or in institutional care, then should not be transported that often
Number of responders: 15

	5	4	3	2	1		Total	Average	Median
Strongly agree	10	4	0	1	0	Strongly disagree	15	4,53	5
	66,66%	26,67%	0%	6,67%	0%				
Total	10	4	0	1	0		15	4,53	5

Multitrauma
Traumatologist: minor benefit
Number of responders: 15

	5	4	3	2	1		Total	Average	Median
Strongly agree	6	6	2	1	0	Strongly disagree	15	4,13	4
	40%	40%	13,33%	6,67%	0%				
Total	6	6	2	1	0		15	4,13	4

Tranexamic acid
Traumatologist: maybe benefit
Number of responders: 15

	5	4	3	2	1		Total	Average	Median
Strongly agree	3	8	1	3	0	Strongly disagree	15	3,73	4
	20%	53,33%	6,67%	20%	0%				
Total	3	8	1	3	0		15	3,73	4

Prophylactic antibiotic administration
Traumatologist: great benefit
Number of responders: 15

	5	4	3	2	1		Total	Average	Median
Strongly agree	2	6	2	2	3	Strongly disagree	15	3,13	4
	13,34%	40%	13,33%	13,33%	20%				
Total	2	6	2	2	3		15	3,13	4

Conscious trauma patient
Traumatologist: great benefit
Number of responders: 15

	5	4	3	2	1		Total	Average	Median
Strongly agree	2	5	3	3	2	Strongly disagree	15	3,13	3
	13,34%	33,33%	20%	20%	13,33%				
Total	2	5	3	3	2		15	3,13	3

Oxygen administration
Traumatologist: maybe benefit
Intensivist 1: Sometimes yes
Intensivist 2: STEMI
Number of responders: 15

	5	4	3	2	1		Total	Average	Median
Strongly agree	1	7	2	5	0	Strongly disagree	15	3,27	4
	6,67%	46,67%	13,33%	33,33%	0%				
Total	1	7	2	5	0		15	3,27	4

Temperature preservation
Traumatologist: maybe benefit
Number of responders: 15

	5	4	3	2	1		Total	Average	Median
Strongly agree	4	5	3	3	0	Strongly disagree	15	3,67	4
	26,67%	33,33%	20%	20%	0%				
Total	4	5	3	3	0		15	3,67	4

Analgesia
Traumatologist: great benefit
Intensivist 1: Sometimes yes
Intensivist 2: STEMI. Intracranial hemorrage of any kind, analgesia to reduce blood pressure.
Number of responders: 15

	5	4	3	2	1		Total	Average	Median
Strongly agree	1	7	3	2	2	Strongly disagree	15	3,2	4
	6,67%	46,67%	20%	13,33%	13,33%				
Total	1	7	3	2	2		15	3,2	4

Spinal stabilization
Traumatologist: maybe benefit
Number of responders: 15

	5	4	3	2	1		Total	Average	Median
Strongly agree	2	3	3	5	2	Strongly disagree	15	2,87	3
	13,34%	20%	20%	33,33%	13,33%				
Total	2	3	3	5	2		15	2,87	3

Severe burns
Traumatologist: great benefit
Number of responders: 15

	5	4	3	2	1		Total	Average	Median
Strongly agree	6	6	1	2	0	Strongly disagree	15	4,07	4
	40%	40%	6,67%	13,33%	0%				
Total	6	6	1	2	0		15	4,07	4

Patients with amputated limbs
Traumatologist: minor benefit
Number of responders: 15

	5	4	3	2	1		Total	Average	Median
Strongly agree	4	6	1	4	0	Strongly disagree	15	3,67	4
	26,66%	40%	6,67%	26,67%	0%				
Total	4	6	1	4	0		15	3,67	4

Head trauma and neuroprotection
Traumatologist: great benefit
Number of responders: 15

	5	4	3	2	1		Total	Average	Median
Strongly agree	11	3	1	0	0	Strongly disagree	15	4,67	5
	73,33%	20%	6,67%	0%	0%				
Total	11	3	1	0	0		15	4,67	5

Ultrasound diagnostics
Traumatologist: minimal benefit
Number of responders: 15

	5	4	3	2	1		Total	Average	Median
Strongly agree	4	2	2	3	4	Strongly disagree	15	2,93	3
	26,67%	13,33%	13,33%	20%	26,67%				
Total	4	2	2	3	4		15	2,93	3

Time saving in transportation with helicopter unit
Traumatologist: maybe benefit
Number of responders: 15

	5	4	3	2	1		Total	Average	Median
Strongly agree	4	8	2	1	0	Strongly disagree	15	4	4
	26,67%	53,33%	13,33%	6,67%	0%				
Total	4	8	2	1	0		15	4	4

Hemodynamic collapse
Intensivist 1: sometimes yes
Intensivist 2: Hypovolemia, less severe septic shock
Number of responders: 13

	5	4	3	2	1		Total	Average	Median
Strongly agree	4	7	1	1	0	Strongly disagree	13	4,08	4
	30,77%	53,85%	7,69%	7,69%	0%				
Total	4	7	1	1	0		13	4,08	4

Extended vasoactive administration (dual or trippel)
Intensivist 1: Very rarely
Intensivist 2: Elevating or lowering BP, inotropes are not usually required prehospitally.
Number of responders: 15

	5	4	3	2	1		Total	Average	Median
Strongly agree	6	2	4	2	1	Strongly disagree	15	3,67	4
	40%	13,33%	26,67%	13,33%	6,67%				
Total	6	2	4	2	1		15	3,67	4

Antidotes and other EMS (paramedic) -level drugs
Intensivist 1: Sometimes yes
Intensivist 2: Charcoal
Number of responders: 15

	5	4	3	2	1		Total	Average	Median
Strongly agree	4	7	2	2	0	Strongly disagree	15	3,87	4
	26,67%	46,67%	13,33%	13,33%	0%				
Total	4	7	2	2	0		15	3,87	4

Sedation
Intensivist 1: Sometimes yes
Intensivist 2: STEMI
Number of responders: 15

	5	4	3	2	1		Total	Average	Median
Strongly agree	4	4	3	3	1	Strongly disagree	15	3,47	4
	26,66%	26,67%	20%	20%	6,67%				
Total	4	4	3	3	1		15	3,47	4

Admittance to involuntary care
Intensivist 1: Sometimes yes
Intensivist 2: Suicidal patients, consultation.
Number of responders: 15

	5	4	3	2	1		Total	Average	Median
Strongly agree	2	3	4	3	3	Strongly disagree	15	2,87	3
	13,33%	20%	26,67%	20%	20%				
Total	2	3	4	3	3		15	2,87	3

Cooling
Intensivist 1: Very rarely
Intensivist 2: Hyperthermia. In other indications the benefit is not established
Number of responders: 15

	5	4	3	2	1		Total	Average	Median
Strongly agree	0	6	4	3	2	Strongly disagree	15	2,93	3
	0%	40%	26,67%	20%	13,33%				
Total	0	6	4	3	2		15	2,93	3

Patient positioning and suctioning
Intensivist 1: Sometimes yes
Intensivist 2: Head trauma, ARF. Suctioning is often life-saving, if mucus is the problem.
Number of responders: 15

	5	4	3	2	1		Total	Average	Median
	1	7	1	6	0		15	3,2	4

Strongly agree	6,67%	46,66%	6,67%	40%	0%	Strongly disagree			
Total	1	7	1	6	0		15	3,2	4

Hyperkalemia treatments
Intensivist 1: Very rarely
Intensivist 2: Glucose and insulin, furosemide. Sodiumbicarbonate
Number of responders: 15

	5	4	3	2	1		Total	Average	Median
Strongly agree	3	3	3	5	1	Strongly disagree	15	3,13	3
	20%	20%	20%	33,33%	6,67%				
Total	3	3	3	5	1		15	3,13	3

Severe intoxication
Intensivist 1: Quite often
Intensivist 2:Hemodynamic support. Charcoal
Number of responders: 15

	5	4	3	2	1		Total	Average	Median
Strongly agree	3	5	5	2	0	Strongly disagree	15	3,6	4
	20%	33,33%	33,33%	13,34%	0%				
Total	3	5	5	2	0		15	3,6	4

Fluid administration
Intensivist 1: Sometimes yes
Intensivist 2: Dehydrated patients
Number of responders: 14

	5	4	3	2	1		Total	Average	Median
Strongly agree	1	5	3	3	2	Strongly disagree	14	3	3
	7,14%	35,71%	21,43%	21,43%	14,29%				
Total	1	5	3	3	2		14	3	3

Simple airway management
Intensivist 1: Sometimes yes
Intensivist 2: Bagmask, CPAP.
Number of responders: 15

	5	4	3	2	1		Total	Average	Median
Strongly agree	1	7	3	3	1	Strongly disagree	15	3,27	4
	6,67%	46,66%	20%	20%	6,67%				
Total	1	7	3	3	1		15	3,27	4

Moderate ABC (airway, breathing, circulation) failure
Intensivist 1: Sometimes yes
Intensivist 2:Laryngeal airway, bag-mask, iv-fluids and short transportation time
Number of responders: 15

	5	4	3	2	1		Total	Average	Median
Strongly agree	3	6	4	1	1	Strongly disagree	15	3,6	4
	20%	40%	26,66%	6,67%	6,67%				
Total	3	6	4	1	1		15	3,6	4

Suicidal patients requiring physician attendance
Intensivist 1: Sometimes yes
Number of responders: 15

	5	4	3	2	1		Total	Average	Median
Strongly agree	2	1	6	3	3	Strongly disagree	15	2,73	3
	13,33%	6,67%	40%	20%	20%				
Total	2	1	6	3	3		15	2,73	3

Hydroxicobalamin administration
Intensivist 1: Sometimes yes
Intensivist 2: House, car or other burn incident, especially inhalational injuries.
Number of responders: 15

	5	4	3	2	1		Total	Average	Median
Strongly agree	2	8	2	2	1	Strongly disagree	15	3,53	4
	13,33%	53,34%	13,33%	13,33%	6,67%				
Total	2	8	2	2	1		15	3,53	4

Patient treated on scene, and hospital transportation avoided
Intensivist 1: Sometimes yes
Intensivist 2: Hypoglycemia
Number of responders: 15

	5	4	3	2	1		Total	Average	Median
Strongly agree	2	3	1	4	5	Strongly disagree	15	2,53	2
	13,33%	20%	6,67%	26,67%	33,33%				
Total	2	3	1	4	5		15	2,53	2

Amputation
Intensivist 1: Very rarely
Intensivist 2: Bleeding control, pain medication because of amputated limbs. Taking care of the amputated limb and taking it to the hospital for replantation.
Number of responders: 15

	5	4	3	2	1		Total	Average	Median
Strongly agree	5	6	2	2	0	Strongly disagree	15	3,93	4
	33,33%	40%	13,34%	13,33%	0%				
Total	5	6	2	2	0		15	3,93	4

Nitroglycerin

Intensivist 1: Sometimes yes
Intensivist 2:CHF
Number of responders: 15

	5	4	3	2	1		Total	Average	Median
Strongly agree	0	3	6	4	2	Strongly disagree	15	2,67	3
	0%	20%	40%	26,67%	13,33%				
Total	0	3	6	4	2		15	2,67	3

Diuretics
Intensivist 1: No
Intensivist 2:If transportation time is long. Requires urethral catetrization.
Number of responders: 15

	5	4	3	2	1		Total	Average	Median
Strongly agree	0	3	3	6	3	Strongly disagree	15	2,4	2
	0%	20%	20%	40%	20%				
Total	0	3	3	6	3		15	2,4	2

Adenosine
Intensivist 1: Very rarely
Intensivist 2:SVT. Differential diagnosis test.
Number of responders: 15

	5	4	3	2	1		Total	Average	Median
Strongly agree	3	5	1	4	2	Strongly disagree	15	3,2	4
	20%	33,33%	6,67%	26,67%	13,33%				
Total	3	5	1	4	2		15	3,2	4

IV glucose
Intensivist 1: Sometimes yes
Intensivist 2: Hypoglycemia.
Number of responders: 15

	5	4	3	2	1		Total	Average	Median
Strongly agree	4	6	1	2	2	Strongly disagree	15	3,53	4
	26,67%	40%	6,67%	13,33%	13,33%				
Total	4	6	1	2	2		15	3,53	4

Transportation of patient in need of a specific center (etc. traumatic amputation and re-implantation)
Intensivist 1: Sometimes yes
Intensivist 2: Head injuries, thoracic injuries, amputations. Pediatric severely ill patients. Thrombectomies for brain insults.
Neurologist: in case of neurological patient, this is often the case (etc. subarachnoid hemorrhage, epidural hematoma, candidate for mechanical thrombectomy)
Number of responders: 15

	5	4	3	2	1		Total	Average	Median

Strongly agree	6	6	1	2	0	Strongly disagree	15	4,07	4
	40%	40%	6,67%	13,33%	0%				
Total	6	6	1	2	0		15	4,07	4

Vasoactives
Intensivist 1: Sometimes yes
Intensivist 2:All patients, if transportation time is long
Number of responders: 15

	5	4	3	2	1		Total	Average	Median
Strongly agree	6	5	4	0	0	Strongly disagree	15	4,13	4
	40%	33,33%	26,67%	0%	0%				
Total	6	5	4	0	0		15	4,13	4

Severe sepsis
Intensivist 1: Very rarely
Intensivist 2:Sepsis in previously healthy/quite healthy and need for hemodynamic support. Long transportation time -> antibiotics
Number of responders: 15

	5	4	3	2	1		Total	Average	Median
Strongly agree	6	6	2	1	0	Strongly disagree	15	4,13	4
	40%	40%	13,33%	6,67%	0%				
Total	6	6	2	1	0		15	4,13	4

Acute lung injury
Intensivist 1: Very rarely
Intensivist 2:Severe hypoxia or hypercarbia in patients without severe chronic pulmonary condition.
Number of responders: 15

	5	4	3	2	1		Total	Average	Median
Strongly agree	3	5	6	1	0	Strongly disagree	15	3,67	4
	20%	33,33%	40%	6,67%	0%				
Total	3	5	6	1	0		15	3,67	4

Meningitis
Intensivist 1: Sometimes yes
Intensivist 2:Antibiotics.
Number of responders: 14

	5	4	3	2	1		Total	Average	Median
Strongly agree	4	6	3	1	0	Strongly disagree	14	3,93	4
	28,57%	42,86%	21,43%	7,14%	0%				
Total	4	6	3	1	0		14	3,93	4

Blood samples
Intensivist 1: Very rarely
Intensivist 2:ABG, blood cultures before antibiotics.
Number of responders: 15

	5	4	3	2	1		Total	Average	Median
Strongly agree	0	2	3	5	5	Strongly disagree	15	2,13	2
	0%	13,34%	20%	33,33%	33,33%				
Total	0	2	3	5	5		15	2,13	2

Long transport time
Intensivist 1: Sometimes yes
Intensivist 2:Transportation, adequate monitoring to tertiary hospital.
Number of responders: 15

	5	4	3	2	1		Total	Average	Median
Strongly agree	1	6	3	3	2	Strongly disagree	15	3,07	3
	6,67%	40%	20%	20%	13,33%				
Total	1	6	3	3	2		15	3,07	3

Necrotizing fasciitis
Intensivist 1: Sometimes yes
Intensivist 2:Antibiotics
Number of responders: 15

	5	4	3	2	1		Total	Average	Median
Strongly agree	3	4	4	2	2	Strongly disagree	15	3,27	3
	20%	26,67%	26,67%	13,33%	13,33%				
Total	3	4	4	2	2		15	3,27	3

Peri-arrest situation managed by physician-staffed EMS-unit
Intensivist 1: Sometimes yes
Intensivist 2:STEMI, non-STEMI, respiratory failure of any reason.
Number of responders: 15

	5	4	3	2	1		Total	Average	Median
Strongly agree	10	4	1	0	0	Strongly disagree	15	4,6	5
	66,66%	26,67%	6,67%	0%	0%				
Total	10	4	1	0	0		15	4,6	5

Non-invasive ventilation
Intensivist 1: Sometimes yes
Intensivist 2:STEMI, non-STEMI, respiratory failure of any reason.
Number of responders: 15

	5	4	3	2	1		Total	Average	Median
Strongly agree	2	8	3	1	1	Strongly disagree	15	3,6	4
	13,33%	53,33%	20%	6,67%	6,67%				
Total	2	8	3	1	1		15	3,6	4

Anaphylactic reaction
Intensivist 1: Yes
Intensivist 2:Iv/sc epinephrine + corticosteroids
Number of responders: 15

	5	4	3	2	1		Total	Average	Median
Strongly agree	4	5	2	3	1	Strongly disagree	15	3,53	4
	26,67%	33,33%	13,33%	20%	6,67%				
Total	4	5	2	3	1		15	3,53	4

Asthma or COPD
Intensivist 1: Sometimes yes
Intensivist 2:Bronchodilators and/or NIV in COPD, oxygen administration
Number of responders: 15

	5	4	3	2	1		Total	Average	Median
Strongly agree	2	7	3	2	1	Strongly disagree	15	3,47	4
	13,33%	46,67%	20%	13,33%	6,67%				
Total	2	7	3	2	1		15	3,47	4

Heart failure
Intensivist 1: Sometimes yes
Intensivist 2:CPAP, nitro, pain medication and ASA
Number of responders: 15

	5	4	3	2	1		Total	Average	Median
Strongly agree	1	9	2	2	1	Strongly disagree	15	3,47	4
	6,67%	60%	13,33%	13,33%	6,67%				
Total	1	9	2	2	1		15	3,47	4

Pneumothorax
Intensivist 1: Rarely
Intensivist 2:CPAP, nitro, pain medication and ASA
Number of responders: 15

	5	4	3	2	1		Total	Average	Median
Strongly agree	4	6	1	3	1	Strongly disagree	15	3,6	4
	26,66%	40%	6,67%	20%	6,67%				
Total	4	6	1	3	1		15	3,6	4

Administration of EMS (paramedic) level drugs
Intensivist 1: Sometimes
Intensivist 2:ASA in STEMI, bronchodilators in atshma/copd, pain medication in stemi or other situation, mild sedation in emotial stress or delirium.
Number of responders: 15

	5	4	3	2	1		Total	Average	Median
Strongly agree	1	3	4	7	0	Strongly disagree	15	2,87	3
	6,67%	20%	26,67%	46,66%	0%				
Total	1	3	4	7	0		15	2,87	3

Airway management excluding rapid sequence intubation (etc. suctioning, bag-masking, foreign object release, oxygen administration)
Intensivist 1: Sometimes yes
Intensivist 2:Resuscitation, foreign object in upper airways, neurological instability eg. GCS decrease. Ineffective breathing, opioid overdose. Desaturation of any cause excl. chronic pulmonary diseases or palliative patients.
Number of responders: 15

	5	4	3	2	1		Total	Average	Median
Strongly agree	4	7	4	0	0	Strongly disagree	15	4	4
	26,67%	46,66%	26,67%	0%	0%				
Total	4	7	4	0	0		15	4	4

Meningitis
Number of responders: 15

	5	4	3	2	1		Total	Average	Median
Strongly agree	2	7	3	2	1	Strongly disagree	15	3,47	4
	13,33%	46,67%	20%	13,33%	6,67%				
Total	2	7	3	2	1		15	3,47	4

Epilepsy
Number of responders: 15

	5	4	3	2	1		Total	Average	Median
Strongly agree	1	5	4	3	2	Strongly disagree	15	3	3
	6,67%	33,33%	26,67%	20%	13,33%				
Total	1	5	4	3	2		15	3	3

Anesthesia intubation
Pediatrician 1: As above; in children, mask or LMA may be sufficient more often than in adults
Pediatrician 2: if skilled person available,ABCDE approach,oxygenation, BM ventilation, medication, intubation, mechanical ventilation
Number of responders: 15

	5	4	3	2	1		Total	Average	Median
Strongly agree	8	4	0	2	1	Strongly disagree	15	4,07	5
	53,33%	26,67%	0%	13,33%	6,67%				
Total	8	4	0	2	1		15	4,07	5

Treatment of hypoglycemia induced seizures
Pediatrician 1: ABCDE approach, oxygenation, BM/SGD ventilation of unconscious patient, treatment of hypoglycemia (10% gluc) and convulsions (benzodiat), sedation, iv/io fluids
Pediatrician 2: yes
Number of responders: 15

	5	4	3	2	1		Total	Average	Median
Strongly agree	6	6	1	2	0	Strongly disagree	15	4,07	4
	40%	40%	6,67%	13,33%	0%				
Total	6	6	1	2	0		15	4,07	4

Pediatric patient in status epilepticus
Pediatrician 1: ABCDE approach, oxygenation, BM/SGD ventilation of unconscious patient, treatment of convulsions (benzodiat), sedation if needed, iv/io fluids
Pediatrician 2: yes
Number of responders: 15

	5	4	3	2	1		Total	Average	Median
Strongly agree	10	3	2	0	0	Strongly disagree	15	4,53	5
	66,67%	20%	13,33%	0%	0%				
Total	10	3	2	0	0		15	4,53	5

Elevated ICP
Pediatrician 1: ABCDE approach, oxygenation, intubation and mechanical ventilation of unconscious patient, treatment of convulsions and fever (benzodiat), sedation, iv/io fluids, vasoactive treatment
Pediatrician 2: yes
Number of responders: 15

	5	4	3	2	1		Total	Average	Median
Strongly agree	8	7	0	0	0	Strongly disagree	15	4,53	5
	53,33%	46,67%	0%	0%	0%				
Total	8	7	0	0	0		15	4,53	5

Surfactant administration
Pediatrician 1: Neonatology and neonatology nurse is taken along to the sceneSurfactant administration of prematures is performed by the expert after intubation
Pediatrician 2: no
Number of responders: 15

	5	4	3	2	1		Total	Average	Median
Strongly agree	2	2	4	4	3	Strongly disagree	15	2,73	3
	13,33%	13,33%	26,67%	26,67%	20%				
Total	2	2	4	4	3		15	2,73	3

Pre-hospital complicated childbirth
Number of responders: 15

	5	4	3	2	1		Total	Average	Median
Strongly agree	6	4	1	4	0	Strongly disagree	15	3,8	4
	40%	26,66%	6,67%	26,67%	0%				
Total	6	4	1	4	0		15	3,8	4

Pre-eclampsia and eclampsia treatments
Number of responders: 14

	5	4	3	2	1		Total	Average	Median
Strongly agree	4	5	3	2	0	Strongly disagree	14	3,79	4
	28,57%	35,71%	21,43%	14,29%	0%				
Total	4	5	3	2	0		14	3,79	4

Resuscitation of a newborn
Pediatrician 1: Neonatology and neonatology nurse is taken along to the scene,Resuscitation is performed by the expert ,If experts are not available, EMS physician/staff will resuscitate the newborn
Pediatrician 2: yes
Number of responders: 15

	5	4	3	2	1		Total	Average	Median
Strongly agree	7	3	2	1	2	Strongly disagree	15	3,8	4
	46,67%	20%	13,33%	6,67%	13,33%				
Total	7	3	2	1	2		15	3,8	4

Haemorrhage controlling maneuvers
Number of responders: 15

	5	4	3	2	1		Total	Average	Median
Strongly agree	6	6	2	1	0	Strongly disagree	15	4,13	4
	40%	40%	13,33%	6,67%	0%				
Total	6	6	2	1	0		15	4,13	4

Heat loss reduction
Pediatrician 1: Vital importance, also in neonates requiring no other measures.
Pediatrician 2: yes
Number of responders: 15

	5	4	3	2	1		Total	Average	Median
Strongly agree	6	3	2	3	1	Strongly disagree	15	3,67	4
	40%	20%	13,33%	20%	6,67%				
Total	6	3	2	3	1		15	3,67	4

Treatment of maternal breathing difficulties
Number of responders: 15

	5	4	3	2	1		Total	Average	Median
Strongly agree	1	5	4	4	1	Strongly disagree	15	3,07	3
	6,67%	33,33%	26,66%	26,67%	6,67%				
Total	1	5	4	4	1		15	3,07	3

Pre-hospital normal delivery
Pediatrician 1: See above (ABC check, hypothermia prevention, transport to hospital)
Number of responders: 15

	5	4	3	2	1		Total	Average	Median
Strongly agree	0	5	5	0	5	Strongly disagree	15	2,67	3
	0%	33,34%	33,33%	0%	33,33%				
Total	0	5	5	0	5		15	2,67	3

Oxitosine administration
Number of responders: 15

	5	4	3	2	1		Total	Average	Median
Strongly agree	0	7	2	2	4	Strongly disagree	15	2,8	3
	0%	46,67%	13,33%	13,33%	26,67%				
Total	0	7	2	2	4		15	2,8	3

Rapid transport
Number of responders: 15

	5	4	3	2	1		Total	Average	Median
Strongly agree	2	5	1	5	2	Strongly disagree	15	3	3
	13,34%	33,33%	6,67%	33,33%	13,33%				
Total	2	5	1	5	2		15	3	3

Asphyxia
Pediatrician 1: Neonatology and neonatology nurse are taken along to the sceneResuscitation, BV-ventilation and intubation is performed by the expert If experts are not available, EMS physician/staff will perform resuscitation, BV-ventilation and intubation of the newborn
Number of responders: 15

	5	4	3	2	1		Total	Average	Median
Strongly agree	6	6	2	0	1	Strongly disagree	15	4,07	4
	40%	40%	13,33%	0%	6,67%				
Total	6	6	2	0	1		15	4,07	4

Hypoglycemia
Pediatrician 1: Neonatology and neonatology nurse are taken along to the sceneResuscitation, BV-ventilation and intubation is performed by the expert If experts are not available, EMS physician/staff will perform resuscitation, BV-ventilation and intubation of the newborn
Number of responders: 15

	5	4	3	2	1		Total	Average	Median
Strongly agree	4	7	1	2	1	Strongly disagree	15	3,73	4
	26,67%	46,66%	6,67%	13,33%	6,67%				
Total	4	7	1	2	1		15	3,73	4

Prolapsed cord and maternal positioning
Number of responders: 15

	5	4	3	2	1		Total	Average	Median
Strongly agree	7	5	1	1	1	Strongly disagree	15	4,07	4
	46,66%	33,33%	6,67%	6,67%	6,67%				
Total	7	5	1	1	1		15	4,07	4

Thrombolytic agents administration
Number of responders: 15

	5	4	3	2	1		Total	Average	Median
Strongly agree	1	4	4	4	2	Strongly disagree	15	2,87	3
	6,67%	26,66%	26,67%	26,67%	13,33%				
Total	1	4	4	4	2		15	2,87	3

Tocolysis
Number of responders: 15

	5	4	3	2	1		Total	Average	Median
Strongly agree	4	3	2	4	2	Strongly disagree	15	3,2	3
	26,67%	20%	13,33%	26,67%	13,33%				
Total	4	3	2	4	2		15	3,2	3

Invasive patient monitoring
Neurologist: Intra-arterial BP monitoring helps to treat hypotension which is especially detrimental for neurosurgical patients.
Number of responders: 15

	5	4	3	2	1		Total	Average	Median
Strongly agree	3	4	3	1	4	Strongly disagree	15	3,07	3
	20%	26,66%	20%	6,67%	26,67%				
Total	3	4	3	1	4		15	3,07	3

Neuroprotective drugs and maneuvers
Neurologist: There are no properly established neuroprotective drugs for neurosurgical patients

Number of responders: 15

	5	4	3	2	1		Total	Average	Median
Strongly agree	4	6	4	1	0	Strongly disagree	15	3,87	4
	26,66%	40%	26,67%	6,67%	0%				
Total	4	6	4	1	0		15	3,87	4

Seizure treatments

Neurologist: Important in level of benzodiazepines (adequate dose) and antiepileptics, if there is enough experince to identify seizure symptoms.

Number of responders: 15

	5	4	3	2	1		Total	Average	Median
Strongly agree	4	7	1	2	1	Strongly disagree	15	3,73	4
	26,67%	46,66%	6,67%	13,33%	6,67%				
Total	4	7	1	2	1		15	3,73	4

ECMO or intra-aortic balloon pump transportation

Cardiologist: In cardiogenic shock or refractary VF , the transfer as soon as possible is essential.

Number of responders: 15

	5	4	3	2	1		Total	Average	Median
Strongly agree	5	3	2	3	2	Strongly disagree	15	3,4	4
	33,33%	20%	13,34%	20%	13,33%				
Total	5	3	2	3	2		15	3,4	4

Pulmonary embolism

Number of responders: 15

	5	4	3	2	1		Total	Average	Median
Strongly agree	3	7	0	4	1	Strongly disagree	15	3,47	4
	20%	46,66%	0%	26,67%	6,67%				
Total	3	7	0	4	1		15	3,47	4

AMI
Cardiologist:Prehospital ASA improves the prognosis.Oxygen is indicated in patients with hypoxaemia
Number of responders: 15

	5	4	3	2	1		Total	Average	Median
Strongly agree	7	1	2	4	1	Strongly disagree	15	3,6	4
	46,66%	6,67%	13,33%	26,67%	6,67%				
Total	7	1	2	4	1		15	3,6	4

Pneumothorax
Number of responders: 15

	5	4	3	2	1		Total	Average	Median
Strongly agree	3	7	1	3	1	Strongly disagree	15	3,53	4
	20%	46,66%	6,67%	20%	6,67%				
Total	3	7	1	3	1		15	3,53	4

UAP
Cardiologist: Prehospital ASA improves the prognosis.Oxygen is indicated in patients with hypoxaemia
Number of responders: 15

	5	4	3	2	1		Total	Average	Median
Strongly agree	2	3	5	4	1	Strongly disagree	15	3,07	3
	13,33%	20%	33,33%	26,67%	6,67%				
Total	2	3	5	4	1		15	3,07	3

Thrombolytic administration
Number of responders: 15

	5	4	3	2	1		Total	Average	Median
Strongly agree	0	8	3	3	1	Strongly disagree	15	3,2	4
	0%	53,33%	20%	20%	6,67%				
Total	0	8	3	3	1		15	3,2	4

External pacing or cardioversion
Number of responders: 15

	5	4	3	2	1		Total	Average	Median
Strongly agree	5	8	1	0	1	Strongly disagree	15	4,07	4
	33,33%	53,33%	6,67%	0%	6,67%				
Total	5	8	1	0	1		15	4,07	4

Thrombolysis due to long transportation time
Number of responders: 15

	5	4	3	2	1		Total	Average	Median
Strongly agree	5	7	2	0	1	Strongly disagree	15	4	4
	33,33%	46,67%	13,33%	0%	6,67%				
Total	5	7	2	0	1		15	4	4

Transportation to PCI
Number of responders: 15

	5	4	3	2	1		Total	Average	Median
Strongly agree	6	5	1	2	1	Strongly disagree	15	3,87	4
	40%	33,33%	6,67%	13,33%	6,67%				
Total	6	5	1	2	1		15	3,87	4

Lung edema
Number of responders: 15

	5	4	3	2	1		Total	Average	Median
Strongly agree	4	7	2	1	1	Strongly disagree	15	3,8	4
	26,67%	46,66%	13,33%	6,67%	6,67%				
Total	4	7	2	1	1		15	3,8	4

Overall resuscitation maneuvers (ALS, BLS) and Post-ROSC treatments
Cardiologist: Post ROSC diagnosis of STEMI is essential and in case of stemi, rapid consultation anf transportation to angiolab is beneficial for patient. It the trasfer is nor possible within 120min , thrombolysis on scene is recommended. Physician is needed for diagnosis and treatment decision on scene or via telephone.
Number of responders: 15

	5	4	3	2	1		Total	Average	Median
Strongly agree	5	6	1	1	2	Strongly disagree	15	3,73	4
	33,33%	40%	6,67%	6,67%	13,33%				
Total	5	6	1	1	2		15	3,73	4

Neonatal and pediatric cardiac arrest
Number of responders: 15

	5	4	3	2	1		Total	Average	Median
Strongly agree	6	5	0	1	3	Strongly disagree	15	3,67	4
	40%	33,33%	0%	6,67%	20%				
Total	6	5	0	1	3		15	3,67	4

Hypovolemic cardiac arrest (etc. need of blood products)
Number of responders: 15

	5	4	3	2	1		Total	Average	Median

Strongly agree	6	4	1	1	3	Strongly disagree	15	3,6	4
	40%	26,66%	6,67%	6,67%	20%				
Total	6	4	1	1	3		15	3,6	4

Cardiac arrest requiring transfer to ECMO or bypass, or ECMO on scene
Number of responders: 15

	5	4	3	2	1		Total	Average	Median
Strongly agree	6	3	2	3	1	Strongly disagree	15	3,67	4
	40%	20%	13,33%	20%	6,67%				
Total	6	3	2	3	1		15	3,67	4

Cardiac arrest and blocked airway
Number of responders: 15

	5	4	3	2	1		Total	Average	Median
Strongly agree	8	3	0	2	2	Strongly disagree	15	3,87	5
	53,34%	20%	0%	13,33%	13,33%				
Total	8	3	0	2	2		15	3,87	5

Cardiac arrest requiring thrombolysis
Number of responders: 15

	5	4	3	2	1		Total	Average	Median
Strongly agree	7	2	2	2	2	Strongly disagree	15	3,67	4
	46,67 %	13,34 %	13,33 %	13,33 %	13,33 %				
Total	7	2	2	2	2		15	3,67	4

Traumatic cardiac arrest
Number of responders: 15

	5	4	3	2	1		Total	Average	Median
Strongly agree	5	3	1	3	3	Strongly disagree	15	3,27	4
	33,33%	20%	6,67%	20%	20%				
Total	5	3	1	3	3		15	3,27	4

Transfer of a resuscitated patient within a time limit
Number of responders: 15

	5	4	3	2	1		Total	Average	Median
Strongly agree	3	6	1	3	2	Strongly disagree	15	3,33	4
	20%	40%	6,67%	20%	13,33%				
Total	3	6	1	3	2		15	3,33	4

Out of algorithm cardiac arrest
Number of responders: 15

	5	4	3	2	1		Total	Average	Median
Strongly agree	3	5	2	2	3	Strongly disagree	15	3,2	4
	20%	33,33%	13,34%	13,33%	20%				
Total	3	5	2	2	3		15	3,2	4

Cardiac arrest requiring anesthesiologist preformed intubation
Number of responders: 15

	5	4	3	2	1		Total	Average	Median
Strongly agree	7	1	0	3	4	Strongly disagree	15	3,27	4
	46,66%	6,67%	0%	20%	26,67%				
Total	7	1	0	3	4		15	3,27	4

Aspirin administration
Cardiologist: Aspirin in case of acute coronary syndrome improve prognosis. Because of good tolerability, ASA administration is suggested even in suspicion of ACS. Score 6
Number of responders: 15

	5	4	3	2	1		Total	Average	Median
Strongly agree	2	2	3	6	2	Strongly disagree	15	2,73	2
	13,34%	13,33%	20%	40%	13,33%				
Total	2	2	3	6	2		15	2,73	2

Drug administration by physician
Number of responders: 15

	5	4	3	2	1		Total	Average	Median
Strongly agree	3	3	3	4	2	Strongly disagree	15	3,07	3
	20%	20%	20%	26,67%	13,33%				
Total	3	3	3	4	2		15	3,07	3

Free comments on HBS 6
Number of responders: 3

**Responses**
Again a lot of unclear points; DC for what?, drug for what?...
"Administration of EMS (paramedic) level drugs": this item is too dependant on the EMS system as the available drugs or authonomy of paramedic may differ: this should not be part of the scale.<br><br>I did not agree on items regarding cardiac arrest for a level 6 as they should only apply to my point of view to level 7
Again, some examples will need a physician on scene

<u>HBS5</u>

Rapid sequence intubation and ventilation
Traumatologist: great benefit
Number of responders: 15

	**5**	**4**	**3**	**2**	**1**		**Total**	**Average**	**Median**
Strongly agree	5	2	3	1	4	Strongly disagree	15	3,2	3
	33,33%	13,33%	20%	6,67%	26,67%				
Total	5	2	3	1	4		15	3,2	3

Oxygen administration
Traumatologist: great benefit
Number of responders: 15

	**5**	**4**	**3**	**2**	**1**		**Total**	**Average**	**Median**
Strongly agree	1	3	4	3	4	Strongly disagree	15	2,6	3
	6,67%	20%	26,66%	20%	26,67%				
Total	1	3	4	3	4		15	2,6	3

Obstructed airway release
Traumatologist: minor benefit
Number of responders: 15

	**5**	**4**	**3**	**2**	**1**		**Total**	**Average**	**Median**
Strongly agree	5	5	1	1	3	Strongly disagree	15	3,53	4
	33,33%	33,33%	6,67%	6,67%	20%				
Total	5	5	1	1	3		15	3,53	4

Reduction and stabilization of fractures or luxations
Traumatologist: minor benefit
Number of responders: 14

	5	4	3	2	1		Total	Average	Median
Strongly agree	0	2	1	7	4	Strongly disagree	14	2,07	2
	0%	14,29%	7,14%	50%	28,57%				
Total	0	2	1	7	4		14	2,07	2

Hypovolemic patients
Traumatologist: minor benefit
Number of responders: 15

	5	4	3	2	1		Total	Average	Median
Strongly agree	1	3	5	2	4	Strongly disagree	15	2,67	3
	6,67%	20%	33,33%	13,33%	26,67%				
Total	1	3	5	2	4		15	2,67	3

Patients treated actively but withdrawl of care in prehospital setting or on arrival to hospital emergency room
Traumatologist: maybe benefit
Number of responders: 15

	5	4	3	2	1		Total	Average	Median
Strongly agree	4	6	3	1	1	Strongly disagree	15	3,73	4
	26,66%	40%	20%	6,67%	6,67%				
Total	4	6	3	1	1		15	3,73	4

Traumatic brain injury and
Traumatologist: great benefit
Number of responders: 15

	5	4	3	2	1		Total	Average	Median
Strongly agree	5	4	4	0	2	Strongly disagree	15	3,67	4
	33,33%	26,67%	26,67%	0%	13,33%				
Total	5	4	4	0	2		15	3,67	4

Severe underlying diseases or conditions or severe head trauma
Traumatologist: great benefit
Number of responders: 15

	5	4	3	2	1		Total	Average	Median
Strongly agree	7	7	0	0	1	Strongly disagree	15	4,27	4
	46,66%	46,67%	0%	0%	6,67%				
Total	7	7	0	0	1		15	4,27	4

Severe trauma or traumatic cardiac arrest
Traumatologist: maybe benefit
Number of responders: 15

	5	4	3	2	1		Total	Average	Median
Strongly agree	3	8	1	1	2	Strongly disagree	15	3,6	4
	20%	53,33%	6,67%	6,67%	13,33%				
Total	3	8	1	1	2		15	3,6	4

Severe hypoxic insult
Intensivist 1: Sometimes yes
Intensivist 2: Long-lasting severe hypoxemia, severe brain injury or mof.
Number of responders: 15

	5	4	3	2	1		Total	Average	Median
Strongly agree	6	6	1	1	1	Strongly disagree	15	4	4
	40%	40%	6,66%	6,67%	6,67%				
Total	6	6	1	1	1		15	4	4

Severe cardiac arrhythmias
Intensivist 1: Sometimes yes
Intensivist 2: Long-lasting severe hypoxemia, severe brain injury or mof.
Number of responders: 15

	5	4	3	2	1		Total	Average	Median
Strongly agree	3	4	3	3	2	Strongly disagree	15	3,2	3
	20%	26,67%	20%	20%	13,33%				
Total	3	4	3	3	2		15	3,2	3

Severe organic phosphate poisoning
Intensivist 1: Very rarely
Intensivist 2: MOF developed.
Number of responders: 15

	5	4	3	2	1		Total	Average	Median
Strongly agree	2	2	5	4	2	Strongly disagree	15	2,87	3
	13,34 %	13,33 %	33,33 %	26,67 %	13,33 %				
Total	2	2	5	4	2		15	2,87	3

Severe strangulation injury
Intensivist 1: Sometimes yes
Intensivist 2:Severe brain injury developed, longlasting strangulation
Number of responders: 15

	5	4	3	2	1		Total	Average	Median
Strongly agree	5	5	2	2	1	Strongly disagree	15	3,73	4
	33,33%	33,33%	13,34%	13,33%	6,67%				
Total	5	5	2	2	1		15	3,73	4

Patients with severe comorbidities
Intensivist 1: Sometimes yes
Intensivist 2:Terminal disease and terminal incident.
Number of responders: 15

	5	4	3	2	1		Total	Average	Median
Strongly agree	2	6	2	3	2	Strongly disagree	15	3,2	4
	13,34%	40%	13,33%	20%	13,33%				
Total	2	6	2	3	2		15	3,2	4

On-scene management/leadership that requires physician-staffed EMS unit
Intensivist 1: Sometimes yes
Intensivist 2:Car crash with multiple minor casualties. Minor burning and inhalation injuries, several casualties.
Number of responders: 15

	5	4	3	2	1		Total	Average	Median
Strongly agree	3	3	2	4	3	Strongly disagree	15	2,93	3
	20%	20%	13,33%	26,67%	20%				
Total	3	3	2	4	3		15	2,93	3

Severe meningitis
Intensivist 1: Very rarely
Intensivist 2:Terminal disease and terminal incident.
Number of responders: 15

	5	4	3	2	1		Total	Average	Median
Strongly agree	3	0	4	4	4	Strongly disagree	15	2,6	2
	20%	0%	26,66%	26,67%	26,67%				
Total	3	0	4	4	4		15	2,6	2

Severe septic shock
Intensivist 1: rarely
Intensivist 2:Severe comorbidities and severe sepsis
Number of responders: 14

	5	4	3	2	1		Total	Average	Median
Strongly agree	3	2	5	1	3	Strongly disagree	14	3,07	3
	21,43%	14,29%	35,71%	7,14%	21,43%				
Total	3	2	5	1	3		14	3,07	3

Patient with severe co-morbidities
Intensivist 1: rarely
Intensivist 2:Severe comorbidities and severe sepsis
Number of responders: 15

	5	4	3	2	1		Total	Average	Median
Strongly agree	4	5	3	2	1	Strongly disagree	15	3,6	4
	26,67%	33,33%	20%	13,33%	6,67%				
Total	4	5	3	2	1		15	3,6	4

Multi-organ failure
Intensivist 1: rarely
Intensivist 2:MOF of any reason in patients with severe co-morbidities.
Number of responders: 15

	5	4	3	2	1		Total	Average	Median
Strongly agree	2	7	2	2	2	Strongly disagree	15	3,33	4
	13,34 %	46,67 %	13,33 %	13,33 %	13,33 %				
Total	2	7	2	2	2		15	3,33	4

Sepsis resulting in cardiac arrest
Intensivist 1: rarely
Intensivist 2:Long ROSC, severe instability after ROSC, severe lactatemia not responding to treatment.
Number of responders: 15

	5	4	3	2	1		Total	Average	Median
Strongly agree	5	6	2	1	1	Strongly disagree	15	3,87	4
	33,33%	40%	13,33%	6,67%	6,67%				
Total	5	6	2	1	1		15	3,87	4

Patient with hypoxic brain injury treated by EMS or physician-staffed EMS –unit
Intensivist 1: Sometimes yes
Intensivist 2:Non-shockable rhythms and long ROSC or unwitnessed or no lay-person cpr.
Number of responders: 15

	5	4	3	2	1		Total	Average	Median
Strongly agree	5	7	1	1	1	Strongly disagree	15	3,93	4
	33,33%	46,66%	6,67%	6,67%	6,67%				
Total	5	7	1	1	1		15	3,93	4

Severe underlying disease with poor prognosis causing breathing difficulties and treated pre-hospitally
Intensivist 1: Sometimes yes
Intensivist 2:Malignancies in palliative care, exacerbation of severe copd (home oxygen or niv), severe neurological disorders (ALS, advanced myastenia etc), severe pulmonary fibrosis.
Number of responders: 15

	5	4	3	2	1		Total	Average	Median
Strongly agree	5	6	2	1	1	Strongly disagree	15	3,87	4
	33,33%	40%	13,33%	6,67%	6,67%				
Total	5	6	2	1	1		15	3,87	4

Airway management
Intensivist 1: Sometimes yes
Intensivist 2:Resuscitation of patients with poor prognosis. Any patient with palliative care. Serious brain damage of any reason (donor candidate, so important anyway)
Number of responders: 15

	5	4	3	2	1		Total	Average	Median
Strongly agree	3	5	2	1	4	Strongly disagree	15	3,13	4
	20%	33,33%	13,33%	6,67%	26,67%				
Total	3	5	2	1	4		15	3,13	4

Trauma with massive haemothorax
Intensivist 1: Sometimes yes
Intensivist 2:Severe damage to heart, lung or large vessels in thorax and polytrauma (head trauma).
Number of responders: 15

	5	4	3	2	1		Total	Average	Median
Strongly agree	1	8	2	1	3	Strongly disagree	15	3,2	4
	6,67%	53,33%	13,33%	6,67%	20%				
Total	1	8	2	1	3		15	3,2	4

Cardiogenic shock and lung edema
Intensivist 1: Sometimes yes
Intensivist 2:Severe heart failure before shock, no chances for revascularization. Severe inactivity and incapability in activities of daily life.
Number of responders: 15

	5	4	3	2	1		Total	Average	Median
Strongly agree	3	7	2	1	2	Strongly disagree	15	3,53	4
	20%	46,67%	13,33%	6,67%	13,33%				
Total	3	7	2	1	2		15	3,53	4

Meningitis
Neurologist: diagnosis rarely possible before hospital
Number of responders: 15

	5	4	3	2	1		Total	Average	Median
Strongly agree	3	2	4	2	4	Strongly disagree	15	2,87	3
	20%	13,33%	26,67%	13,33%	26,67%				
Total	3	2	4	2	4		15	2,87	3

Status Epilepticus
Neurologist: I and II phase treatments
Number of responders: 15

	5	4	3	2	1		Total	Average	Median
Strongly agree	1	3	3	3	5	Strongly disagree	15	2,47	2
	6,67%	20%	20%	20%	33,33%				
Total	1	3	3	3	5		15	2,47	2

Intracerebral tumors
Neurologist: Undiagnosed intracranial tumors can cause a medical emergency by provoking a seizure or by obstructing CSF flow resulting in hydrocephalus (mainly posterior fossa tumors). Intratumor hemorrhage can also produce acute symptoms by increasing the overall mass effect. Intracranial tumors cannot be validly even suspected in the pre-hospital setting as the clinical presentation of the disease can by very variable. The EMS treatment should be steered by the signs/symptoms (level of consciousness, seizures etc.). Quite seldomly, patients with a known (already diagnosed) intracranial tumor present to the EMS with an acute pre-hospital treatment-demanding problem.
Number of responders: 15

	5	4	3	2	1		Total	Average	Median
Strongly agree	1	4	4	4	2	Strongly disagree	15	2,87	3
	6,67%	26,66%	26,67%	26,67%	13,33%				
Total	1	4	4	4	2		15	2,87	3

Terminally ill patients

Neurologist: Patients with malignant brain tumors (mainly glioblastoma) that are already delineated outside neurosurgical treatment most likely would not benefit from intensive pre-hospital or emergency treatment.

Number of responders: 15

	5	4	3	2	1		Total	Average	Median
Strongly agree	5	4	1	3	2	Strongly disagree	15	3,47	4
	33,33%	26,67%	6,67%	20%	13,33%				
Total	5	4	1	3	2		15	3,47	4

Hypoglycemia treatments

Number of responders: 15

	5	4	3	2	1		Total	Average	Median
Strongly agree	0	2	2	1	10	Strongly disagree	15	1,73	1
	0%	13,33%	13,33%	6,67%	66,67%				
Total	0	2	2	1	10		15	1,73	1

Pre-hospital hysterectomy

Gynaecologist: cannot imagine when this would be needed

Number of responders: 15

	5	4	3	2	1		Total	Average	Median
Strongly agree	1	1	4	0	9	Strongly disagree	15	2	1
	6,67%	6,67%	26,66%	0%	60%				
Total	1	1	4	0	9		15	2	1

Severe hypoxia and/or asphyxia of a newborn

Gynaecologist: prehospital treatment improves the prognosis

Number of responders: 15

	5	4	3	2	1		Total	Average	Median
Strongly agree	3	6	1	1	4	Strongly disagree	15	3,2	4
	20%	40%	6,67%	6,67%	26,66%				
Total	3	6	1	1	4		15	3,2	4

Extremely pre-term childbirth (<24 gw) born pre-hospitally
Gynaecologist: prehospital treatment improves the prognosis (from 23 gw on)
Number of responders: 15

	5	4	3	2	1		Total	Average	Median
Strongly agree	5	2	3	2	3	Strongly disagree	15	3,27	3
	33,33%	13,34%	20%	13,33%	20%				
Total	5	2	3	2	3		15	3,27	3

Severe maternal fluid (or other) embolism
Gynaecologist:prompt effective resuscitation improves the prognosis
Number of responders: 15

	5	4	3	2	1		Total	Average	Median
Strongly agree	2	2	2	3	6	Strongly disagree	15	2,4	2
	13,34%	13,33%	13,33%	20%	40%				
Total	2	2	2	3	6		15	2,4	2

Pro-longed resuscitation of a newborn
Gynaecologist:agree
Number of responders: 15

	5	4	3	2	1		Total	Average	Median
Strongly agree	7	5	1	2	0	Strongly disagree	15	4,13	4
	46,67%	33,33%	6,67%	13,33%	0%				
Total	7	5	1	2	0		15	4,13	4

Resuscitation
Gynaecologist: treatment outside hospital definitely improves prognosis, which may be good
Number of responders: 15

	5	4	3	2	1		Total	Average	Median
Strongly agree	5	2	1	3	4	Strongly disagree	15	3,07	3
	33,33%	13,33%	6,67%	20%	26,67%				
Total	5	2	1	3	4		15	3,07	3

Cardiac arrest of a pregnant patient
Gynaecologist: treatment outside hospital definitely improves prognosis, which may be good
Number of responders: 15

	5	4	3	2	1		Total	Average	Median
Strongly agree	5	3	1	0	6	Strongly disagree	15	3,07	4
	33,33%	20%	6,67%	0%	40%				
Total	5	3	1	0	6		15	3,07	4

Patient treated but due severe symptoms and/or underlying diseases has a poor prognosis

Neurologist: If patient is already a mRS >3 (Modified Rankin Scale for Neurologic Disability) before the prehospital incident, and in need of assistance for everyday-life or in institutional care, then should not be transported that often
Number of responders: 15

	5	4	3	2	1		Total	Average	Median
Strongly agree	6	5	3	1	0	Strongly disagree	15	4,07	4
	40%	33,33%	20%	6,67%	0%				
Total	6	5	3	1	0		15	4,07	4

Some haemorhagic strokes
Number of responders: 15

	5	4	3	2	1		Total	Average	Median
Strongly agree	4	7	2	2	0	Strongly disagree	15	3,87	4
	26,67%	46,67%	13,33%	13,33%	0%				
Total	4	7	2	2	0		15	3,87	4

Basic ventilator support
Number of responders: 15

	5	4	3	2	1		Total	Average	Median
Strongly agree	1	3	4	2	5	Strongly disagree	15	2,53	3
	6,67%	20%	26,67%	13,33%	33,33%				
Total	1	3	4	2	5		15	2,53	3

Massive pulmonary embolism
Cardiologist: thrombolysis improves the prognosis
Number of responders: 15

	5	4	3	2	1		Total	Average	Median
Strongly agree	2	6	1	3	3	Strongly disagree	15	3,07	4
	13,33%	40%	6,67%	20%	20%				
Total	2	6	1	3	3		15	3,07	4

Massive AMI

Cardiologist: Diagnosis, ASA, rapid transfer to PCI center. Oxygen. Treatment of arrhythmias

Number of responders: 15

	5	4	3	2	1		Total	Average	Median
Strongly agree	3	4	2	1	5	Strongly disagree	15	2,93	3
	20%	26,67%	13,33%	6,67%	33,33%				
Total	3	4	2	1	5		15	2,93	3

Antiarrhytmic therapy

Number of responders: 15

	5	4	3	2	1		Total	Average	Median
Strongly agree	2	2	3	2	6	Strongly disagree	15	2,47	2
	13,34%	13,33%	20%	13,33%	40%				
Total	2	2	3	2	6		15	2,47	2

Thoracic aneurysm

Number of responders: 15

	5	4	3	2	1		Total	Average	Median
Strongly agree	2	4	4	3	2	Strongly disagree	15	3,07	3
	13,33%	26,67%	26,67%	20%	13,33%				
Total	2	4	4	3	2		15	3,07	3

Cardiac arrest due to severe traumatic brain injury or subarachnoid haemorrhage

Number of responders: 15

	5	4	3	2	1		Total	Average	Median
Strongly agree	9	2	3	1	0	Strongly disagree	15	4,27	5
	60%	13,33%	20%	6,67%	0%				
Total	9	2	3	1	0		15	4,27	5

Cardiac arrest that received no bystander-CPR prior EMS unit performed BLS/ALS (basic or advanced life support)

Cardiologist: Prognosis of patient with cardiac arrest and no bystander-CPR is poor if no-flow time is over 10 minutes and resuscitation efforts may be futile.

Number of responders: 15

	5	4	3	2	1		Total	Average	Median
Strongly agree	3	5	4	2	1	Strongly disagree	15	3,47	4
	20%	33,33%	26,67%	13,33%	6,67%				
Total	3	5	4	2	1		15	3,47	4

Withdraw of treatments during CPR or post-ROSC

Number of responders: 15

	5	4	3	2	1		Total	Average	Median
Strongly agree	4	4	1	1	5	Strongly disagree	15	3,07	4
	26,66%	26,67%	6,67%	6,67%	33,33%				
Total	4	4	1	1	5		15	3,07	4

Poor prognosis of patient post-ROSC (due to prolonged resuscitation or underlying diseases)
Number of responders: 14

	5	4	3	2	1		Total	Average	Median
Strongly agree	7	5	1	1	0	Strongly disagree	14	4,29	4,5
	50%	35,72%	7,14%	7,14%	0%				
Total	7	5	1	1	0		14	4,29	4,5

Free comments on HBS 5
Number of responders: 2

Responses
"112. Traumatic brain injury and ": incomplete question
In my opinion, the prognosis should not be evaluated on-scene, if the patient is anyhow actively treated. It is almost impossible e.g. after cardiac arrest. I have resuscitated young patients with short delay to ROSC who have died anyway, but controversally older ones with ROSC >45 min surviving very well to home. Same problem with head trauma.

HBS4

Analgesia
Traumatologist: maybe benefit
Number of responders: 15

	5	4	3	2	1		Total	Average	Median
Strongly agree	4	4	3	2	2	Strongly disagree	15	3,4	4
	26,67%	26,67%	20%	13,33%	13,33%				
Total	4	4	3	2	2		15	3,4	4

Minor injuries
Traumatologist: no benefit
Number of responders: 15

	5	4	3	2	1		Total	Average	Median
Strongly agree	2	7	3	2	1	Strongly disagree	15	3,47	4
	13,33%	46,67%	20%	13,33%	6,67%				
Total	2	7	3	2	1		15	3,47	4

Traumatic cardiac arrest without ROSC
Traumatologist: no benefit
Number of responders: 15

	5	4	3	2	1		Total	Average	Median
Strongly agree	4	3	4	0	4	Strongly disagree	15	3,2	3
	26,66%	20%	26,67%	0%	26,67%				
Total	4	3	4	0	4		15	3,2	3

Antibiotic administration
Traumatologist: minimal benefit
Number of responders: 15

	5	4	3	2	1		Total	Average	Median
Strongly agree	4	5	3	2	1	Strongly disagree	15	3,6	4
	26,67%	33,33%	20%	13,33%	6,67%				
Total	4	5	3	2	1		15	3,6	4

Reduction and stabilization of fractures (minor)
Traumatologist: no benefit
Number of responders: 15

	5	4	3	2	1		Total	Average	Median
Strongly agree	0	8	0	6	1	Strongly disagree	15	3	4
	0%	53,33%	0%	40%	6,67%				
Total	0	8	0	6	1		15	3	4

IV fluid administration
Traumatologist: minimal benefit
Number of responders: 15

	5	4	3	2	1		Total	Average	Median
Strongly agree	2	6	1	5	1	Strongly disagree	15	3,2	4
	13,33%	40%	6,67%	33,33%	6,67%				
Total	2	6	1	5	1		15	3,2	4

Tranexamic acid administration
Traumatologist: minimal benefit
Neurologist: Pre-hospitally admistered TXA seem benefial for TBI patients.
Number of responders: 15

	5	4	3	2	1		Total	Average	Median
Strongly agree	3	9	1	1	1	Strongly disagree	15	3,8	4
	20%	60%	6,66%	6,67%	6,67%				
Total	3	9	1	1	1		15	3,8	4

Trauma patient immobilisation (etc. cervical collar, back board)
Traumatologist: minimal benefit
Number of responders: 15

	5	4	3	2	1		Total	Average	Median
Strongly agree	3	8	2	1	1	Strongly disagree	15	3,73	4
	20%	53,33%	13,33%	6,67%	6,67%				
Total	3	8	2	1	1		15	3,73	4

Severe underlying diseases or conditions, or severe head trauma and poor prognosis
Traumatologist: minor benefit
Number of responders: 15

	5	4	3	2	1		Total	Average	Median
Strongly agree	2	3	4	2	4	Strongly disagree	15	2,8	3
	13,33%	20%	26,67%	13,33%	26,67%				
Total	2	3	4	2	4		15	2,8	3

Mannitol administration
Traumatologist: no benefit
Number of responders: 15

	5	4	3	2	1		Total	Average	Median
Strongly agree	2	3	3	5	2	Strongly disagree	15	2,87	3
	13,34%	20%	20%	33,33%	13,33%				
Total	2	3	3	5	2		15	2,87	3

Rapid sequence intubation in trauma patients
Traumatologist: minor benefit
Number of responders: 15

	5	4	3	2	1		Total	Average	Median
Strongly agree	3	2	3	2	5	Strongly disagree	15	2,73	3
	20%	13,34%	20%	13,33%	33,33%				
Total	3	2	3	2	5		15	2,73	3

Triage and patient selection and transportation to dedicated center
Traumatologist: great benefit
Number of responders: 15

	5	4	3	2	1		Total	Average	Median
Strongly agree	3	5	2	4	1	Strongly disagree	15	3,33	4
	20%	33,33%	13,33%	26,67%	6,67%				
Total	3	5	2	4	1		15	3,33	4

Antidotes
Intensivist 1: Sometimes yes
Intensivist 2: Can usually be given in the hospital
Number of responders: 15

	5	4	3	2	1		Total	Average	Median
Strongly agree	2	5	2	5	1	Strongly disagree	15	3,13	3
	13,34%	33,33%	13,33%	33,33%	6,67%				
Total	2	5	2	5	1		15	3,13	3

Minor symptoms
Intensivist 1: Sometimes yes
Intensivist 2: Headache and pain medication
Pediatrician 1: in children, may be very important to treat minor symptoms such as pain or dehydration in order to be able to evaluate the situation correctly
Pediatrician 2: Analgesia etc.when needed
Number of responders: 15

	5	4	3	2	1		Total	Average	Median
Strongly agree	1	8	1	3	2	Strongly disagree	15	3,2	4
	6,67%	53,33%	6,67%	20%	13,33%				
Total	1	8	1	3	2		15	3,2	4

Charcoal administration
Intensivist 1: Often yes
Intensivist 2: Possible intoxication.
Number of responders: 15

	5	4	3	2	1		Total	Average	Median
Strongly agree	1	4	3	3	4	Strongly disagree	15	2,67	3
	6,67%	26,66%	20%	20%	26,67%				
Total	1	4	3	3	4		15	2,67	3

Calcium channel blocker intoxication
Intensivist 1: Sometimes yes
Intensivist 2: Charcoal administration, rapid transportation.
Number of responders: 15

	5	4	3	2	1		Total	Average	Median
Strongly agree	0	6	3	3	3	Strongly disagree	15	2,8	3
	0%	40%	20%	20%	20%				
Total	0	6	3	3	3		15	2,8	3

Corticoids for allergic reaction
Intensivist 1: Sometimes yes
Intensivist 2: Any non-minor allergic reaction.
Number of responders: 15

	5	4	3	2	1		Total	Average	Median
Strongly agree	2	6	2	4	1	Strongly disagree	15	3,27	4
	13,33%	40%	13,33%	26,67%	6,67%				
Total	2	6	2	4	1		15	3,27	4

Antihistamin for allergic reaction
Intensivist 1: Sometimes yes
Intensivist 2: Any allergic reaction.
Number of responders: 15

	5	4	3	2	1		Total	Average	Median
Strongly agree	0	8	2	1	4	Strongly disagree	15	2,93	4
	0%	53,33%	13,33%	6,67%	26,67%				
Total	0	8	2	1	4		15	2,93	4

Antibiotics (excluding sepsis or meningitis)
Intensivist 1: Sometimes yes
Intensivist 2: Not applicable.
Number of responders: 15

	5	4	3	2	1		Total	Average	Median
Strongly agree	3	7	2	1	2	Strongly disagree	15	3,53	4
	20%	46,67%	13,33%	6,67%	13,33%				
Total	3	7	2	1	2		15	3,53	4

Fluid administration
Intensivist 1: Quite often
Intensivist 2: Dehydration or mild hypovolemia, severe emesis or diarrhea.
Number of responders: 15

	5	4	3	2	1		Total	Average	Median
Strongly agree	0	10	3	1	1	Strongly disagree	15	3,47	4
	0%	66,66%	20%	6,67%	6,67%				
Total	0	10	3	1	1		15	3,47	4

Long transportation
Intensivist 1: Sometimes yes
Intensivist 2: Transportation to a tertiary hospital with unknown TBI.
Number of responders: 15

	5	4	3	2	1		Total	Average	Median
Strongly agree	3	5	4	1	2	Strongly disagree	15	3,4	4
	20%	33,33%	26,67%	6,67%	13,33%				
Total	3	5	4	1	2		15	3,4	4

Blood cultures
Intensivist 1: Sometimes yes
Intensivist 2: It should be routine in the hospitals to take blood cultures before antibiotics, so this is not applicable.
Number of responders: 15

	5	4	3	2	1		Total	Average	Median
Strongly agree	2	3	5	1	4	Strongly disagree	15	2,87	3
	13,33%	20%	33,33%	6,67%	26,67%				
Total	2	3	5	1	4		15	2,87	3

Oxygen administration
Intensivist 1: Often yes
Intensivist 2: Patients with chest pain of unknown origin. Mild hypoxemia from any cause. Trauma patients with mild hypoxemia.
Number of responders: 15

	5	4	3	2	1		Total	Average	Median
Strongly agree	2	7	2	3	1	Strongly disagree	15	3,4	4
	13,33%	46,67%	13,33%	20%	6,67%				
Total	2	7	2	3	1		15	3,4	4

Asthma or COPD
Intensivist 1: Often yes
Intensivist 2: Acute asthma or copd exacerbation, bronchodilatators as treatment. Severe asthma with severe obstruction and exhaustion not included.
Number of responders: 15

	5	4	3	2	1		Total	Average	Median
Strongly agree	3	6	3	1	2	Strongly disagree	15	3,47	4
	20%	40%	20%	6,67%	13,33%				
Total	3	6	3	1	2		15	3,47	4

Prehospital non-invasive ventilation for COPD
Intensivist 1: Often yes
Intensivist 2: Mild exacerbation
Number of responders: 15

	5	4	3	2	1		Total	Average	Median
Strongly agree	5	2	3	3	2	Strongly disagree	15	3,33	3
	33,33%	13,34%	20%	20%	13,33%				
Total	5	2	3	3	2		15	3,33	3

Prehospital inhalational therapy for COLD/pneumonia
Intensivist 1: Often yes
Intensivist 2: Mild exacerbation, mild obstruction, mild breathing difficulties
Number of responders: 15

	5	4	3	2	1		Total	Average	Median
Strongly agree	3	8	4	0	0	Strongly disagree	15	3,93	4
	20%	53,33%	26,67%	0%	0%				
Total	3	8	4	0	0		15	3,93	4

Moderate breathing difficulty treated by EMS unit
Intensivist 1: Often yes
Intensivist 2: Cardiogenic edema in patients with previously diagnosed CHF. Chronical neurological disorders, pulmonary fibrosis exacerbation.
Number of responders: 15

	5	4	3	2	1		Total	Average	Median
Strongly agree	3	8	1	3	0	Strongly disagree	15	3,73	4
	20%	53,33%	6,67%	20%	0%				
Total	3	8	1	3	0		15	3,73	4

Nebulizer treatment
Intensivist 1: Often yes
Intensivist 2: Cardiogenic edema in patients with previously diagnosed CHF. Chronical neurological disorders, pulmonary fibrosis exacerbation.
Number of responders: 15

	5	4	3	2	1		Total	Average	Median
Strongly agree	1	9	2	3	0	Strongly disagree	15	3,53	4
	6,67%	60%	13,33%	20%	0%				
Total	1	9	2	3	0		15	3,53	4

Rectal, intranasal or sublingual drug administration
Pediatrician 1: May often be needed in children as iv access can be tricky
Pediatrician 2: analgesia/treatemtn of convulsiones
Number of responders: 15

	**5**	**4**	**3**	**2**	**1**		**Total**	**Average**	**Median**
Strongly agree	2	4	5	2	2	Strongly disagree	15	3,13	3
	13,34 %	26,67 %	33,33 %	13,33 %	13,33 %				
Total	2	4	5	2	2		15	3,13	3

Treatment of hypertensive crisis
Pediatrician 1: rare in children
Pediatrician 2: ABCDE approach, antihypertensive medication, BP measurement, transport to the hospital
Number of responders: 15

	**5**	**4**	**3**	**2**	**1**		**Total**	**Average**	**Median**
Strongly agree	2	3	4	3	3	Strongly disagree	15	2,87	3
	13,33%	20%	26,67%	20%	20%				
Total	2	3	4	3	3		15	2,87	3

I.v. cannulation
Pediatrician 1: As important in children as in adults - should not overtly delay transport. I.n. and p.o. often feasible for medication, intraosseal approach should also be encouraged
Pediatrician 2: if fluids/medication needed before hospital treatment
Number of responders: 15

	**5**	**4**	**3**	**2**	**1**		**Total**	**Average**	**Median**
Strongly agree	2	7	2	2	2	Strongly disagree	15	3,33	4
	13,34 %	46,67 %	13,33 %	13,33 %	13,33 %				
Total	2	7	2	2	2		15	3,33	4

Treatment of patient with GCS <13
Pediatrician: as in adults: repetitive evaluation, prompt transport, airway securisation
Number of responders: 15

	**5**	**4**	**3**	**2**	**1**		**Total**	**Average**	**Median**
Strongly agree	5	4	2	2	2	Strongly disagree	15	3,53	4
	33,33 %	26,67 %	13,34 %	13,33 %	13,33 %				
Total	5	4	2	2	2		15	3,53	4

Treatment of short seizures
Pediatrician 1: Essential to prevent prolonged seizures
Pediatrician 2: Benzodiatzepines if more than one short (<5 min) seizure, transport to the hospital, if several short seizures or first time
Number of responders: 15

	5	4	3	2	1		Total	Average	Median
Strongly agree	4	7	2	2	0	Strongly disagree	15	3,87	4
	26,67%	46,67%	13,33%	13,33%	0%				
Total	4	7	2	2	0		15	3,87	4

Pre-hospital normal childbirth
Pediatrician: Very important . EMS have an important role in evaluating if the newborn is OK (ABC) and in preventing hypothermia.
Number of responders: 15

	5	4	3	2	1		Total	Average	Median
Strongly agree	5	6	2	2	0	Strongly disagree	15	3,93	4
	33,33%	40%	13,34%	13,33%	0%				
Total	5	6	2	2	0		15	3,93	4

Pre-hospital maternal or newborn death
Pediatrician: EMS evaluation and treatment potentilally very important for the family ("everything was being done"),
Number of responders: 15

	5	4	3	2	1		Total	Average	Median
Strongly agree	4	4	3	0	4	Strongly disagree	15	3,27	4
	26,66%	26,67%	20%	0%	26,67%				
Total	4	4	3	0	4		15	3,27	4

Maternal heat loss prevention

Number of responders: 15

	5	4	3	2	1		Total	Average	Median
Strongly agree	1	8	3	2	1	Strongly disagree	15	3,4	4
	6,67%	53,33%	20%	13,33%	6,67%				
Total	1	8	3	2	1		15	3,4	4

Tocolysis

Number of responders: 15

	5	4	3	2	1		Total	Average	Median
Strongly agree	4	3	4	0	4	Strongly disagree	15	3,2	3
	26,66%	20%	26,67%	0%	26,67%				
Total	4	3	4	0	4		15	3,2	3

Maternal positioning

Number of responders: 15

	5	4	3	2	1		Total	Average	Median
Strongly agree	3	5	1	4	2	Strongly disagree	15	3,2	4
	20%	33,33%	6,67%	26,67%	13,33%				
Total	3	5	1	4	2		15	3,2	4

Oxitosine administration

Number of responders: 15

	5	4	3	2	1		Total	Average	Median
Strongly agree	3	4	2	3	3	Strongly disagree	15	3,07	3
	20%	26,67%	13,33%	20%	20%				
Total	3	4	2	3	3		15	3,07	3

Steroids for lung maturation

Pediatrican: Normally not needed unless very long transport tie (> 2-3 h)

Number of responders: 15

	5	4	3	2	1		Total	Average	Median
Strongly agree	1	3	3	1	7	Strongly disagree	15	2,33	2
	6,67%	20%	20%	6,67%	46,66%				
Total	1	3	3	1	7		15	2,33	2

Temperature control
Neurologist:There is no solid evidence that hypothermia is neuroprotective, although some center use hypothermia for intractable ICP.Normothermia is the goal in treatment.
Number of responders: 15

	5	4	3	2	1		Total	Average	Median
Strongly agree	0	8	2	3	2	Strongly disagree	15	3,07	4
	0%	53,34%	13,33%	20%	13,33%				
Total	0	8	2	3	2		15	3,07	4

Hyperclycemia treatments

Number of responders: 14

	5	4	3	2	1		Total	Average	Median
Strongly agree	2	6	1	3	2	Strongly disagree	14	3,21	4
	14,28%	42,86%	7,14%	21,43%	14,29%				
Total	2	6	1	3	2		14	3,21	4

Any pre-hospital treatments with stroke patients
Neurologist: Essential is to measure and take care over 38 celsius body temperature, blood glucose level and nausea, although the evidence could be slightly thin and over 220mmhg systolic blood pressure.
Number of responders: 15

	5	4	3	2	1		Total	Average	Median
Strongly agree	2	8	0	3	2	Strongly disagree	15	3,33	4
	13,33%	53,34%	0%	20%	13,33%				
Total	2	8	0	3	2		15	3,33	4

Advanced circulatory support

Number of responders: 15

	5	4	3	2	1		Total	Average	Median
Strongly agree	3	6	2	2	2	Strongly disagree	15	3,4	4
	20%	40%	13,34%	13,33%	13,33%				
Total	3	6	2	2	2		15	3,4	4

Basic ABCD measurements and diagnosis
Neurologist: Is relevant
Number of responders: 15

	5	4	3	2	1		Total	Average	Median
Strongly agree	4	6	0	3	2	Strongly disagree	15	3,47	4
	26,67%	40%	0%	20%	13,33%				
Total	4	6	0	3	2		15	3,47	4

Non-STEMI or UAP treatments

Number of responders: 15

	5	4	3	2	1		Total	Average	Median
Strongly agree	2	7	2	3	1	Strongly disagree	15	3,4	4
	13,33%	46,67%	13,33%	20%	6,67%				
Total	2	7	2	3	1		15	3,4	4

Angina pectoris

Number of responders: 15

	5	4	3	2	1		Total	Average	Median
Strongly agree	2	6	3	3	1	Strongly disagree	15	3,33	4
	13,33%	40%	20%	20%	6,67%				
Total	2	6	3	3	1		15	3,33	4

Medical treatment on-scene

Number of responders: 15

	5	4	3	2	1		Total	Average	Median
Strongly agree	3	4	4	2	2	Strongly disagree	15	3,27	3
	20%	26,67%	26,67%	13,33%	13,33%				
Total	3	4	4	2	2		15	3,27	3

Defibrillation during cardiac arrest

Number of responders: 15

	5	4	3	2	1		Total	Average	Median
Strongly agree	3	3	0	3	6	Strongly disagree	15	2,6	2
	20%	20%	0%	20%	40%				
Total	3	3	0	3	6		15	2,6	2

Cardiac arrest without ROSC

Cardiologist: Every patient with VF should be evaluated by protocol, whether the patient is candidate for ECPR (echo assisted resuscitation) which might be life saving incase of no ROSC with conventional resuscitation. Physician is needed for decision.

Number of responders: 15

	5	4	3	2	1		Total	Average	Median
Strongly agree	5	4	2	0	4	Strongly disagree	15	3,4	4
	33,33%	26,67%	13,33%	0%	26,67%				
Total	5	4	2	0	4		15	3,4	4

Signs of death and no indication for resuscitation maneuvers

Number of responders: 15

	5	4	3	2	1		Total	Average	Median
Strongly agree	1	4	2	2	6	Strongly disagree	15	2,47	2
	6,67%	26,67%	13,33%	13,33%	40%				
Total	1	4	2	2	6		15	2,47	2

Prolonged resuscitation with ROSC

Number of responders: 15

	5	4	3	2	1		Total	Average	Median
Strongly agree	4	3	2	3	3	Strongly disagree	15	3,13	3
	26,67%	20%	13,33%	20%	20%				
Total	4	3	2	3	3		15	3,13	3

Resuscitation medication

Number of responders: 15

	5	4	3	2	1		Total	Average	Median
Strongly agree	2	5	2	3	3	Strongly disagree	15	3	3
	13,34%	33,33%	13,33%	20%	20%				
Total	2	5	2	3	3		15	3	3

Intubation as a primary airway management

Number of responders: 15

	5	4	3	2	1		Total	Average	Median
Strongly agree	3	4	3	1	4	Strongly disagree	15	3,07	3
	20%	26,66%	20%	6,67%	26,67%				
Total	3	4	3	1	4		15	3,07	3

Cardiac arrest and hypothermia

Number of responders: 14

	5	4	3	2	1		Total	Average	Median
Strongly agree	3	2	4	3	2	Strongly disagree	14	3,07	3
	21,43 %	14,28 %	28,57 %	21,43 %	14,29 %				
Total	3	2	4	3	2		14	3,07	3

Known cardiac history, and chest pain without ST-elevation

Cardiologist: Acute coronary syndrome should be suspected and aspirin as well as heparin (or lmwh) given because these have effect to prognosis.. Also pain medication, treatment of high blood pressure might benefit the patient.

Number of responders: 15

	5	4	3	2	1		Total	Average	Median
Strongly agree	3	7	2	3	0	Strongly disagree	15	3,67	4
	20%	46,67%	13,33%	20%	0%				
Total	3	7	2	3	0		15	3,67	4

Bystander CPR resulting in ROSC prior EMS arrival

Number of responders: 14

	5	4	3	2	1		Total	Average	Median
Strongly agree	3	2	3	1	5	Strongly disagree	14	2,79	3
	21,43%	14,29%	21,43%	7,14%	35,71%				
Total	3	2	3	1	5		14	2,79	3

Free comments on HBS 4
Number of responders: 1

Responses
There are way to many statements that does not make any sense; e.g. # 1 or #54 - analgesia for what? to whoom? intubation of whoom? by whoom? for what???

HBS3

Analgesia
Traumatologist: minor benefit
Intensivist 1: Often Yes
Intensivist 2: Any pain.
Vastaajien määrä: 15

	5	4	3	2	1		Total	Average	Median
Strongly agree	8	7	0	0	0	Strongly disagree	15	4,53	5
	53,33%	46,67%	0%	0%	0%				
Total	8	7	0	0	0		15	4,53	5

Minor injuries
Traumatologist: no benefit
Vastaajien määrä: 15

	5	4	3	2	1		Total	Average	Median
Strongly agree	3	4	5	3	0	Strongly disagree	15	3,47	3
	20%	26,67%	33,33%	20%	0%				
Total	3	4	5	3	0		15	3,47	3

Reassurance, and information for relatives/by-standers/etc.
Traumatologist: minor benefit
Vastaajien määrä: 15

	5	4	3	2	1		Total	Average	Median
Strongly agree	7	3	3	1	1	Strongly disagree	15	3,93	4
	46,66%	20%	20%	6,67%	6,67%				
Total	7	3	3	1	1		15	3,93	4

Reduction and stabilization of fractures (minor)
Traumatologist: no benefit
Vastaajien määrä: 15

	5	4	3	2	1		Total	Average	Median
Strongly agree	4	6	3	1	1	Strongly disagree	15	3,73	4
	26,66%	40%	20%	6,67%	6,67%				

Total	4	6	3	1	1		15	3,73	4

Patients who die pre-hospitally
Intensivist 1: Rarely
Intensivist 2: Terminal disease, pain medication.
Vastaajien määrä: 14

	5	4	3	2	1		Total	Average	Median
Strongly agree	4	3	2	0	5	Strongly disagree	14	3,07	3,5
	28,57%	21,43%	14,29%	0%	35,71%				
Total	4	3	2	0	5		14	3,07	3,5

Patients with minor symptoms
Intensivist 1: Sometimes yes
Intensivist 2: Any relief to the symptoms
Vastaajien määrä: 15

	5	4	3	2	1		Total	Average	Median
Strongly agree	0	5	3	2	5	Strongly disagree	15	2,53	3
	0%	33,33%	20%	13,34%	33,33%				
Total	0	5	3	2	5		15	2,53	3

Sedation
Intensivist 1: Sometimes yes
Intensivist 2: Hyperventilation
Vastaajien määrä: 15

	5	4	3	2	1		Total	Average	Median
Strongly agree	1	8	4	1	1	Strongly disagree	15	3,47	4
	6,67%	53,33%	26,66%	6,67%	6,67%				
Total	1	8	4	1	1		15	3,47	4

Naloxone administration
Intensivist 1: Quite often
Intensivist 2: Minor opioid abuse
Vastaajien määrä: 15

	5	4	3	2	1		Total	Average	Median
Strongly agree	2	3	2	3	5	Strongly disagree	15	2,6	2
	13,34%	20%	13,33%	20%	33,33%				
Total	2	3	2	3	5		15	2,6	2

Antihistamins for allergic reaction
Intensivist 1: Sometimes yes
Intensivist 2: Any allergic reaction
Vastaajien määrä: 15

	5	4	3	2	1		Total	Average	Median
Strongly agree	5	6	3	0	1	Strongly disagree	15	3,93	4
	33,33%	40%	20%	0%	6,67%				
Total	5	6	3	0	1		15	3,93	4

Agonal patient, or patient who died pre-hospitally
Intensivist 1: Sometimes yes
Intensivist 2: Palliative care
Vastaajien määrä: 14

	5	4	3	2	1		Total	Average	Median
Strongly agree	3	3	4	1	3	Strongly disagree	14	3,14	3
	21,43%	21,43%	28,57%	7,14%	21,43%				
Total	3	3	4	1	3		14	3,14	3

Oxygen administration with normal range saturation
Intensivist 1: Quite often
Intensivist 2: Smoke inhalation. Mild desaturation after pain medication/sedation.
Vastaajien määrä: 14

	5	4	3	2	1		Total	Average	Median
Strongly agree	1	1	3	4	5	Strongly disagree	14	2,21	2
	7,14%	7,14%	21,43%	28,57%	35,72%				
Total	1	1	3	4	5		14	2,21	2

Fluids administration in normovolemia and without hypotension
Intensivist 1: Quite often
Intensivist 2: Smoke inhalation. Mild desaturation after pain medication/sedation.
Vastaajien määrä: 15

	5	4	3	2	1		Total	Average	Median
Strongly agree	0	2	4	4	5	Strongly disagree	15	2,2	2
	0%	13,33%	26,67%	26,67%	33,33%				
Total	0	2	4	4	5		15	2,2	2

Antipyretics
Intensivist 1: Quite often
Intensivist 2: Febrile convulsions, children with high fever, brings comfort to the patients
Vastaajien määrä: 15

	5	4	3	2	1		Total	Average	Median
Strongly agree	4	4	2	4	1	Strongly disagree	15	3,4	4
	26,66%	26,67%	13,33%	26,67%	6,67%				
Total	4	4	2	4	1		15	3,4	4

Opioid administration in respiratory insufficiency
Intensivist 1: Sometimes yes
Intensivist 2: Any cause for difficult breathing, acute or chronic.
Vastaajien määrä: 15

	5	4	3	2	1		Total	Average	Median
Strongly agree	4	7	1	2	1	Strongly disagree	15	3,73	4
	26,67%	46,66%	6,67%	13,33%	6,67%				
Total	4	7	1	2	1		15	3,73	4

Adrenal inhalation in pseudocroup
Intensivist 1: Sometimes yes
Intensivist 2: Pseudocroup, otherwise ok.
Vastaajien määrä: 15

	5	4	3	2	1		Total	Average	Median
Strongly agree	5	4	1	3	2	Strongly disagree	15	3,47	4
	33,33%	26,67%	6,67%	20%	13,33%				
Total	5	4	1	3	2		15	3,47	4

Bentsodiazepines for anxiety
Intensivist 1: Sometimes yes
Intensivist 2: Mental anxiety of any cause, delirium tremens. Non-cardiac origin chest pain/feelings.
Vastaajien määrä: 15

	5	4	3	2	1		Total	Average	Median
Strongly agree	3	7	1	3	1	Strongly disagree	15	3,53	4
	20%	46,66%	6,67%	20%	6,67%				
Total	3	7	1	3	1		15	3,53	4

Inhalation administration
Intensivist 1: Sometimes yes
Intensivist 2: Smoke injuries. Asthma
Vastaajien määrä: 15

	5	4	3	2	1		Total	Average	Median
Strongly agree	0	9	2	3	1	Strongly disagree	15	3,27	4
	0%	60%	13,33%	20%	6,67%				
Total	0	9	2	3	1		15	3,27	4

Pneumonia
Intensivist 1: Sometimes yes
Intensivist 2: Non-severe pneumonia, but need for hospitalization (saturation ok, but general condition weakened).
Vastaajien määrä: 15

	5	4	3	2	1		Total	Average	Median
Strongly agree	0	4	6	2	3	Strongly disagree	15	2,73	3
	0%	26,67%	40%	13,33%	20%				
Total	0	4	6	2	3		15	2,73	3

Body positioning

Vastaajien määrä: 15

	5	4	3	2	1		Total	Average	Median
Strongly agree	1	5	4	1	4	Strongly disagree	15	2,87	3
	6,67%	33,33%	26,66%	6,67%	26,67%				
Total	1	5	4	1	4		15	2,87	3

Rectal/buccal/intranasal drug administration
Pediatrician 1: for analgesia or treatment of convulsions
Pediatrician 2: if needed
Vastaajien määrä: 15

	5	4	3	2	1		Total	Average	Median
Strongly agree	3	5	3	2	2	Strongly disagree	15	3,33	4
	20%	33,33%	20%	13,34%	13,33%				
Total	3	5	3	2	2		15	3,33	4

Treatment of postictal state
Pediatrician: support oxygenation
Vastaajien määrä: 15

	5	4	3	2	1		Total	Average	Median
Strongly agree	4	4	3	3	1	Strongly disagree	15	3,47	4
	26,66%	26,67%	20%	20%	6,67%				
Total	4	4	3	3	1		15	3,47	4

Pre-hospital normal childbirth
Pediatrician: Very important . EMS have an important role in evaluating if the newborn is OK (ABC) and in preventing hypothermia.
Vastaajien määrä: 15

	5	4	3	2	1		Total	Average	Median
Strongly agree	3	6	2	1	3	Strongly disagree	15	3,33	4
	20%	40%	13,33%	6,67%	20%				
Total	3	6	2	1	3		15	3,33	4

Blood glucose monitoring
Neurologist: Relevant if over 8
Vastaajien määrä: 15

	5	4	3	2	1		Total	Average	Median
Strongly agree	4	0	1	5	5	Strongly disagree	15	2,53	2
	26,67%	0%	6,67%	33,33%	33,33%				
Total	4	0	1	5	5		15	2,53	2

Patient outside therapeutic window (long delay)
Neurologist: Still good care will lessen damage
Vastaajien määrä: 14

	5	4	3	2	1		Total	Average	Median
Strongly agree	3	2	3	1	5	Strongly disagree	14	2,79	3
	21,43%	14,29%	21,43%	7,14%	35,71%				
Total	3	2	3	1	5		14	2,79	3

Antiemetic medication

Vastaajien määrä: 15

	5	4	3	2	1		Total	Average	Median
Strongly agree	4	7	1	2	1	Strongly disagree	15	3,73	4
	26,67%	46,66%	6,67%	13,33%	6,67%				
Total	4	7	1	2	1		15	3,73	4

Anxiety treatments

Vastaajien määrä: 15

	5	4	3	2	1		Total	Average	Median
Strongly agree	4	6	3	2	0	Strongly disagree	15	3,8	4
	26,67%	40%	20%	13,33%	0%				
Total	4	6	3	2	0		15	3,8	4

Minor pneumothorax

Vastaajien määrä: 15

	5	4	3	2	1		Total	Average	Median
Strongly agree	1	6	3	3	2	Strongly disagree	15	3,07	3
	6,67%	40%	20%	20%	13,33%				
Total	1	6	3	3	2		15	3,07	3

Broken ribs

Vastaajien määrä: 15

	5	4	3	2	1		Total	Average	Median
Strongly agree	1	7	2	2	3	Strongly disagree	15	3,07	4
	6,67%	46,67%	13,33%	13,33%	20%				
Total	1	7	2	2	3		15	3,07	4

DNAR (do-not attempt resuscitation) decision made on-scene

Vastaajien määrä: 14

	5	4	3	2	1		Total	Average	Median
Strongly agree	5	3	3	0	3	Strongly disagree	14	3,5	4
	35,71%	21,43%	21,43%	0%	21,43%				
Total	5	3	3	0	3		14	3,5	4

Resuscitation determined based on incomplete information of patient background

Vastaajien määrä: 15

	5	4	3	2	1		Total	Average	Median
Strongly agree	4	2	4	1	4	Strongly disagree	15	3,07	3
	26,66%	13,33%	26,67%	6,67%	26,67%				
Total	4	2	4	1	4		15	3,07	3

Free comments on HBS 3

Vastaajien määrä: 3

Responses
Again; many examples of illness or symptoms instead of interventions makes it really hard to score...
A bit difficult to assess, as some of the Qs represent diagnoses, and some dedidicated treatments with an evidence base.
In current HEMS benefit score, this class is underestimated. Why you can get almost twice as much points to save a life without prognosis (and to cause more suffering at the same time), but only 3 points, to relieve terrific pain?
