# Supplementary material for: The development of emergency medical services benefit score: a European Delphi study
Source: Scand J Trauma Resusc Emerg Med. 2021 Oct 16;29:151. doi: 10.1186/s13049-021-00966-3 (PMC8520267; doi:10.1186/s13049-021-00966-3)
Supplement: Supplementary file 2 — Additional file 2. The material of third Delphi round. [file 13049_2021_966_MOESM2_ESM.pdf]

Additional file 2. The third Delphi round

Total number of responders: 16

EBS CATEGORY 8

Thoracotomy or tamponade release with other maneuvers

Number of responders: 16

	n	Percent
Accept	15	93,75%
Delete	1	6,25%
Relocate to EBS category number:	0	0%

Suggested relocations

Option names	Category

Thoracostomy or pleural drainage followed by a relief of signs and symptoms

Number of responders: 16

	n	Percent
Accept	16	100%
Delete	0	0%
Relocate to EBS category number:	0	0%

Suggested relocations

Option names	Text

Administration of physician-staffed EMS level medication (medication not allowed in other units) followed by a relief of signs and symptoms

Number of responders: **16**

	n	Percent
Accept	11	68,75%
Delete	1	6,25%
Relocate to EBS category number:	4	25%

Suggested relocations

Option names	Text
Relocate to EBS category number:	6
Relocate to EBS category number:	7
Relocate to EBS category number:	6
Relocate to EBS category number:	6

Mass casualty incident leadership and triage

Number of responders: **16**

	n	Percent
Accept	10	62,5%
Delete	2	12,5%
Relocate to EBS category number:	4	25%

Suggested relocations

Option names	Text
Relocate to EBS category number:	7
Relocate to EBS category number:	6
Relocate to EBS category number:	7
Relocate to EBS category number:	4

Intubation and ventilation of a premature or a newborn baby

Number of responders: 16

	n	Percent
Accept	13	81,25%
Delete	1	6,25%
Relocate to EBS category number:	2	12,5%

Suggested relocations

Option names	Text
Relocate to EBS category number:	6
Relocate to EBS category number:	6

Prehospital caesarean section (resuscitative hysterectomy)

Number of responders: 16

	n	Percent
Accept	14	87,5%
Delete	2	12,5%
Relocate to EBS category number:	0	0%

Suggested relocations

Option names	Text

Resuscitation of a newborn by bag-mask ventilation or by more advanced procedures

Number of responders: 16

	n	Percent
Accept	12	75%
Delete	0	0%
Relocate to EBS category number:	4	25%

Suggested relocations

Option names	Text
Relocate to EBS category number:	7
Relocate to EBS category number:	7
Relocate to EBS category number:	6
Relocate to EBS category number:	7

Rapid sequence intubation or surgical airway management, and mechanical ventilation

Number of responders: 16

	n	Percent
Accept	13	81,25%
Delete	1	6,25%
Relocate to EBS category number:	2	12,5%

Suggested relocations

Option names	Text
Relocate to EBS category number:	6
Relocate to EBS category number:	6

Treatment and stabilizing of a multi-trauma patient in a shock by vasoactive medication and/or i.v. fluid administration

Number of responders: **16**

	n	Percent
Accept	11	68,75%
Delete	0	0%
Relocate to EBS category number:	5	31,25%

Suggested relocations

Option names	Text
Relocate to EBS category number:	7
Relocate to EBS category number:	6
Relocate to EBS category number:	7
Relocate to EBS category number:	6
Relocate to EBS category number:	6

Blood product transfusions

Number of responders: 16

	n	Percent
Accept	13	81,25%
Delete	1	6,25%
Relocate to EBS category number:	2	12,5%

Suggested relocations

Option names	Text
Relocate to EBS category number:	6
Relocate to EBS category number:	6

FREE COMMENTS ON EBS 8 (etc. interventions that should be included)

Number of responders: 6

Responses
Pediatric RSI
No further interventions recommended.<br>I suggest a wording change, if at all possible:<br>"Treatment and stabilizing of a multi-trauma patient in shock by i.v. fluid administration and/or vasoactive medication" instead of:<br>"Treatment and stabilizing of a multi-trauma patient in a shock by vasoactive medication and/or i.v. fluid administration"
There are some maneuvers that may be "possible" for some other units, but in my opinion, should not be, e.g. pleural drainage. Of course, anyone can learn the maneuver with a manikin, but safe procedure requires clinical knowledge.
Successful airway management after failed attempts by other providers.
Intubation per se does not improve prognosis in every patient. If located in cat. 8, should be defined by reason. Same is true in multitraumapatient with circulatory shock. Vasoactive drugs does not per se do not always improve prognosis, but may save lives until hospital.
Should include:<br>- ECPR initiated in prehospital phase<br>- airway management in case of seriously compromised airway and other personnel unable do secure it

EBS CATEGORY 7

Isolated severe trauma managed with simple maneuvers (etc. direct compression, tourniquet)

Number of responders: 15

	n	Percent
Accept	14	93,33%
Delete	0	0%
Relocate to EBS category number:	1	6,67%

Suggested relocations

Option names	Text
Relocate to EBS category number:	6

Needle thoracocentesis followed by a relief of signs or symptoms

Number of responders: 16

	n	Percent
Accept	15	93,75%
Delete	1	6,25%
Relocate to EBS category number:	0	0%

Suggested relocations

Option names	Text

Cardioversion or cardiac pacing

Number of responders: 16

	n	Percent
Accept	13	81,25%
Delete	1	6,25%
Relocate to EBS category number:	2	12,5%

Suggested relocations

Option names	Text
Relocate to EBS category number:	8
Relocate to EBS category number:	6

Medication (adrenalin/epinephrine) in anaphylactic shock

Number of responders: 16

	n	Percent
Accept	15	93,75%
Delete	0	0%
Relocate to EBS category number:	1	6,25%

Suggested relocations

Option names	Text
Relocate to EBS category number:	6

Medication for circulatory support (i.v. ephedrine, i.v. noradrenalin/norepinephrine etc.)

Number of responders: **16**

	n	Percent
Accept	9	56,25%
Delete	0	0%
Relocate to EBS category number:	7	43,75%

Suggested relocations

Option names	Text
Relocate to EBS category number:	6
Relocate to EBS category number:	8
Relocate to EBS category number:	5-6
Relocate to EBS category number:	6
Relocate to EBS category number:	8
Relocate to EBS category number:	6
Relocate to EBS category number:	6

Treatment of prolonged seizures by first, or second line i.v. medication (bentsodiazepines or phosphenytoin, etc.)

Number of responders: **16**

	n	Percent
Accept	10	62,5%
Delete	0	0%
Relocate to EBS category number:	6	37,5%

Suggested relocations

Option names	Text
Relocate to EBS category number:	6
Relocate to EBS category number:	6
Relocate to EBS category number:	6
Relocate to EBS category number:	6
Relocate to EBS category number:	5
Relocate to EBS category number:	6

Treatment of hypoglycemia induced coma or seizures by i.v. glucose or s.c./i.m. glucagon

Number of responders: **16**

	n	Percent
Accept	11	68,75%
Delete	0	0%
Relocate to EBS category number:	5	31,25%

Suggested relocations

Option names	Text
Relocate to EBS category number:	6
Relocate to EBS category number:	6
Relocate to EBS category number:	6
Relocate to EBS category number:	6
Relocate to EBS category number:	6

Management of complicated childbirth (shoulder dystocia, malposition, etc.)

Number of responders: **16**

	n	Percent
Accept	10	62,5%
Delete	1	6,25%
Relocate to EBS category number:	5	31,25%

Suggested relocations

Option names	Text
Relocate to EBS category number:	8
Relocate to EBS category number:	8
Relocate to EBS category number:	8
Relocate to EBS category number:	8
Relocate to EBS category number:	8

Successful resuscitation with reasonable prognosis

Number of responders: 16

	n	Percent
Accept	16	100%
Delete	0	0%
Relocate to EBS category number:	0	0%

Suggested relocations

Option names	Text

Transfer to ECMO or bypass while CPR

Number of responders: 16

	n	Percent
Accept	12	75%
Delete	1	6,25%
Relocate to EBS category number:	3	18,75%

Suggested relocations

Option names	Text
Relocate to EBS category number:	5
Relocate to EBS category number:	8

Relocate to EBS category number:	8

Manual opening of an obstructed airway and bag-mask ventilation

Number of responders: 16

	n	Percent
Accept	15	93,75%
Delete	0	0%
Relocate to EBS category number:	1	6,25%

Suggested relocations

Option names	Text
Relocate to EBS category number:	6

Use of a supraglottic device and bag-mask ventilation

Number of responders: **16**

	n	Percent
Accept	11	68,75%
Delete	2	12,5%
Relocate to EBS category number:	3	18,75%

Suggested relocations

Option names	Text
Relocate to EBS category number:	6
Relocate to EBS category number:	6
Relocate to EBS category number:	6

FREE COMMENTS ON EBS 7 (etc. interventions that should be included)

Number of responders: 3

Responses
- perhaps you should change in the definition "the patient would have died" to "the patient could have died". In many of the examples, such as anaphylaxic shock or cardioversion, the patient would often probably still have reached the hospital alive, even without any intervention. This is particularly true in systems with short transport times to hospitals, such as The Netherlands.<br>- Needle thoracocentesis is generally not recommended any longer by most protocols, e.g., ATLS, and should therefore not serve as an example.
Manouvers that do not influence on prognosis in long sight, should by my opinion be in cat 5 or 6. In 5, if patients prognosis is poor and in 6, if can not estimated.
The adequate HBS for "Use of a supraglottic device and bag-mask ventilation" depends on the indication and the status of spontaneous airway.

EBS CATEGORY 6

Reduction and stabilization of major fractures or luxations

Number of responders: 16

	n	Percent
Accept	15	93,75%
Delete	0	0%
Relocate to EBS category number:	1	6,25%

Suggested relocations

Option names	Text
Relocate to EBS category number:	4

Triage and patient selection to dedicated center and rapid transportation (major trauma, TBI, need of thrombectomy, need for re-implantation in traumatic amputation, etc.)

Number of responders: 16

	n	Percent
Accept	16	100%
Delete	0	0%
Relocate to EBS category number:	0	0%

Suggested relocations

Option names	Text

Treatment of opioid or benzodiazepine poisoning by antidotes

Number of responders: 16

	n	Percent
Accept	13	81,25%
Delete	0	0%
Relocate to EBS category number:	3	18,75%

Suggested relocations

Option names	Text
Relocate to EBS category number:	7
Relocate to EBS category number:	5
Relocate to EBS category number:	7

Treatment of hypoglycemia by i.v. glucose or s.c./i.m. glucagon when patient is disoriented, but not in coma

Number of responders: 16

	n	Percent
Accept	14	87,5%
Delete	0	0%
Relocate to EBS category number:	2	12,5%

Suggested relocations

Option names	Text
Relocate to EBS category number:	5
Relocate to EBS category number:	4

Maternal positioning in case of prolapsed umbilical cord

Number of responders: 16

	n	Percent
Accept	14	87,5%
Delete	0	0%
Relocate to EBS category number:	2	12,5%

Suggested relocations

Option names	Text
Relocate to EBS category number:	7
Relocate to EBS category number:	7

Thrombolysis for STEMI in case with a long transportation time

Number of responders: 16

	n	Percent
Accept	16	100%
Delete	0	0%

Relocate to EBS category number:	0	0%

Suggested relocations

Option names	Text

Rapid transportation to PCI

Number of responders: 16

	n	Percent
Accept	16	100%
Delete	0	0%
Relocate to EBS category number:	0	0%

Suggested relocations

Option names	Text

32. FREE COMMENTS ON EBS 6 (etc. interventions that should be included)

Number of responders: 0

Responses

EBS CATEGORY 5

Patient treated but due severe symptoms and/or underlying diseases has a poor prognosis (etc., severe trauma or traumatic cardiac arrest, severe hypoxic insult, pro-longed resuscitation, cardiac arrest due to severe traumatic brain injury or subarachnoid haemorrhage)

Number of responders: 16

	n	Percent
Accept	15	93,75%
Delete	1	6,25%
Relocate to EBS category number:	0	0%

Suggested relocations

Option names	Text

FREE COMMENTS ON EBS 5 (etc. interventions that should be included)

Number of responders: 2

Responses
Wording recommendation: "Patient treated, but due TO severe....."
This category is the most difficult one. Assessing the prognosis is very difficult. We all know that patients resuscitated from VF with relatively short delays may still die, and some with prolonged resusciation (>60 minutes) may be tertiary survivors.

EBS CATEGORY 4

Administration of tranexamic acid

Number of responders: 16

	n	Percent
Accept	12	75%
Delete	1	6,25%
Relocate to EBS category number:	3	18,75%

Suggested relocations

Option names	Text
Relocate to EBS category number:	6
Relocate to EBS category number:	6
Relocate to EBS category number:	6

Trauma patient immobilization (cervical collar, back board, etc.)

Number of responders: 16

	n	Percent
Accept	14	87,5%
Delete	0	0%
Relocate to EBS category number:	2	12,5%

Suggested relocations

Option names	Text

Relocate to EBS category number:	6
Relocate to EBS category number:	3

Administration of inhaled bronchodilators for COPD or pneumonia

Number of responders: 16

	n	Percent
Accept	14	87,5%
Delete	0	0%
Relocate to EBS category number:	2	12,5%

Suggested relocations

Option names	Text
Relocate to EBS category number:	6
Relocate to EBS category number:	6

Administration of oxygenation in moderate breathing difficulty

Number of responders: 16

	n	Percent
Accept	13	81,25%
Delete	0	0%
Relocate to EBS category number:	3	18,75%

Suggested relocations

Option names	Text
Relocate to EBS category number:	6
Relocate to EBS category number:	6
Relocate to EBS category number:	3

FREE COMMENTS ON EBS 4 (etc. interventions that should be included)

Number of responders: 0

Responses

EBS CATEGORY 3

Administration of analgesics

Number of responders: 15

	n	Percent
Accept	14	93,33%
Delete	0	0%
Relocate to EBS category number:	1	6,67%

Suggested relocations

Option names	Text
Relocate to EBS category number:	4

Administration of antihistamines to treat an allergic reaction

Number of responders: 16

	n	Percent
Accept	14	87,5%
Delete	1	6,25%
Relocate to EBS category number:	1	6,25%

Suggested relocations

Option names	Text
Relocate to EBS category number:	4

Administration of opioids in respiratory insufficiency

Number of responders: 16

	n	Percent
Accept	9	56,25%
Delete	5	31,25%
Relocate to EBS category number:	2	12,5%

Suggested relocations

Option names	Text
Relocate to EBS category number:	4
Relocate to EBS category number:	4

Antiemetic medication

Number of responders: 16

	n	Percent
Accept	14	87,5%
Delete	1	6,25%
Relocate to EBS category number:	1	6,25%

Suggested relocations

Option names	Text
Relocate to EBS category number:	2

FREE COMMENTS ON EBS 3 (etc. interventions that should be included)

Number of responders: 3

Responses
In my opinion the patients who die before reaching the hospital but who have benefitted from the care e.g. analgesia should be included in this category.
Opioids for resp insufficency? Not an indication in Norway? Is the purpose anxiolytic?
Opioids in breathing insufficiency is not standard treatment. Should be just be used to calm the patient and increase approval of CPAP/BiPAP
